# Identification of hotspots of crop wild relatives in Germany to promote their in situ conservation in a network of genetic reserves

**DOI:** 10.1186/s40529-025-00473-z

**Published:** 2025-09-02

**Authors:** Maria Bönisch, Vera Senße, Thomas Engst, Alica Sander, Diethart Matthies, Eckhard Jedicke, Nadine Bernhardt

**Affiliations:** 1 Institute for Resistance Research and Stress Tolerance, Julius Kühn-Institut (JKI) - Federal Research Centre for Cultivated Plants, Quedlinburg, Germany; 2 Department of Agriculture, Ecotrophology and Landscape Development, Anhalt University of Applied Sciences, Bernburg, Germany; 3 Plant Ecology and Geobotany, Department of Biology, Philipps-Universität Marburg, Marburg, Germany; 4 Department of Landscape Planning & Nature Conservation, Hochschule Geisenheim University, Geisenheim, Germany

**Keywords:** Genetic resources, Species inventory, Hotspot analysis, Plants for food and agriculture

## Abstract

**Background:**

Crop wild relatives (CWR) represent a valuable resource for ensuring food security. Although they are negatively affected by the loss of habitats due to climate change and land use change, they are underrepresented in conservation programmes. The establishment of genetic reserve (GR) networks has been put forward as an approach to protect CWR. The objective of this study was to identify CWR hotspots in Germany, which are suitable candidates for the establishment of GRs. CWR observation data were collected on a national scale from various sources and a hotspot analysis was performed to identify potential GR sites. A number of these sites were visited and the plant species occurring were recorded.

**Results:**

76 GR candidate sites were identified. The sites are distributed across the biogeographic regions of Germany and could conserve 73% of all CWR and 85% of the CWR that were assigned a conservation priority for Germany. Our on-site surveys for 27 GR candidates found discrepancies between the species records available and the species currently occurring.

**Conclusions:**

We propose five hectares as an appropriate size for GRs, as such an area can be monitored and the number of stakeholders involved is usually low. The discrepancies concerning species records highlight the need for more frequent, regular nationwide CWR monitoring as a prerequisite for their conservation. Our results further suggest that GRs should also be set up outside of protected areas to protect CWR efficiently.

**Supplementary Information:**

The online version contains supplementary material available at 10.1186/s40529-025-00473-z.

## Introduction

In the face of ongoing climatic change, it is necessary to develop stress-resilient crops to ensure food security (Kole 2011; Eckardt et al. 2023). Crop wild relatives (CWR) and their intraspecific genetic diversity represent an important genetic resource for the improvement of crops because they can hold or develop traits that allow crops to cope with specific biotic and abiotic stressors (Gerdemann-Knörck et al. 1994; Mammadov et al. 2018; Gong et al. 2022).

Due to the effects of climate change and changes in land use, populations of CWR are declining (e.g. Jain 1975; Maxted et al. 1997; Meilleur and Hodgkin 2004; Maxted et al. 2007; Aguirre-Gutiérrez et al. 2017; Khoury et al. 2020; van Treuren et al. 2020; Raggi et al. 2024). The gradual decline in the number and size of populations results in a loss of intraspecific genetic diversity (Leigh et al. 2019; Stange et al. 2021). This can endanger the potential of taxa to adapt to changing environmental conditions (Kéry et al. 2000; Weigel 2012) and thus increase their risk of extinction (Bilz et al. 2011; Goettsch et al. 2021; Wambugu and Henry 2022). Compared to crops, CWR are still underrepresented in genebanks (Castañeda-Álvarez et al. 2011; Wambugu and Henry 2022), although some progress has been made in recent years (CGRFA 2023). The in situ conservation of CWR has been recognized as an essential approach to safeguarding genetic resources (Maxted et al. 1997). For example, the International Treaty on Plant Genetic Resources for Food and Agriculture (ITPGRFA) demands to conserve CWR in situ and to re-establish viable populations in protected areas (FAO 2001). In addition, the Aichi Biodiversity Target 13 of the Convention on Biological Diversity (CBD 2010) demands the safeguarding of the genetic diversity of wild relatives of cultivated plants.

Hawkes et al. (1997) described the genetic reserve (GR) conservation technique, which aims at conserving a set of evolving populations that represent the intraspecific diversity of a target taxon. The monitoring of intraspecific diversity and the preservation of seeds ex situ are also components of this technique (Maxted and Kell 2009; Maxted et al. 2015). Quality standards for GRs and their networking have been proposed (Iriondo et al. 2012; Maxted et al. 2016). GRs should represent different natural habitats to cover local adaptations (Whitlock et al. 2016). The local stakeholders should be willing to cooperate in establishing GRs and their subsequent management. Sites located in existing protected areas should be preferentially selected since greater sustainability and efficacy can be assumed. However, lately, it has increasingly been proposed to also include sites outside protected areas to conserve CWR because a significant part of the genetic diversity of CWR is located in non-protected areas (Jarvis et al. 2015; Vincent et al. 2022; Kägi et al. 2023; Petitpierre et al. 2023; Mewis et al. 2024).

A growing number of studies has been conducted in various countries with the objective of protecting CWR (Fielder et al. 2015; Frese et al. 2018; Khoury et al. 2020; van Treuren et al. 2020; Magos Brehm et al. 2022; Kägi et al. 2023; Mewis et al. 2024; Raggi et al. 2024). To protect the CWR in a country, the first step is to carry out a national inventory that can be used to prioritise efforts to conserve CWR (Taylor et al. 2017; Bönisch and Thormann 2021; El Mokni et al. 2022; Petitpierre et al. 2023). Various approaches, and e.g. an online toolkit, have already been developed to identify areas for in situ conservation, including qualitative analyses, spatial statistics, and modelling (Magos Brehm et al. 2017; Sussman et al. 2019; Iannella et al. 2020).

In Germany, 3,651 vascular plant species occur (Metzing et al. 2018). There are 2,877 CWR taxa listed in the National Inventory of Plant Genetic Resources (PGRDEU). Some other wild plant species, although not related to a crop, have also been included, as they may also be valuable for food and agriculture (BLE 2019). They may become crops through neo-domestication (e.g. *Urtica dioica* L., Eckardt et al. 2023). A quarter of the CWR are considered endangered (Metzing 2020). To prioritise conservation efforts, a draft list of priority CWR has been compiled (BEKO 2019; Bönisch and Thormann 2021). The list takes into account both, the economic relevance and the global conservation responsibility of Germany for the individual CWR (Ludwig et al. 2007).

Several studies were conducted in Germany aiming at the in situ conservation of CWR, taking into account their intraspecific diversity, e.g. on *Vitis vinifera* L. ssp. *sylvestris* C. C. Gmel. (Ledesma-Krist et al. 2013), on CWR in species-rich grasslands (Gradl et al. 2022; Lehmair et al. 2022) and on the CWR of celery (Frese et al. 2018; Bönisch et al. 2020; Herden et al. 2020; Mewis et al. 2024). Some GRs have already been established, e.g. for celery CWR (Frese et al. 2018; Bönisch et al. 2020). As an overarching framework, the German Network of Genetic Reserves was initiated (Bönisch and Thormann 2021).

To date, most studies have investigated potential GRs for single or a few taxa in Germany. The objective of this study was to identify CWR hotspots in Germany to facilitate efficient and comprehensive conservation of the genetic diversity of (priority) CWR and contribute to the further growth of the German Network of Genetic Reserves. We collected German-wide data on species occurrences and conducted a hotspot analysis. We then selected GR candidates in each biogeographic region and considered different vegetation types to cover a large number of CWR. In addition, we made an on-site inventory of the species occurring at 27 of the GR candidate sites.

The data were used to address the following questions: (1) Do the currently available data allow the reliable identification of CWR hotspots in Germany? (2) Where are the most important hotspots of (priority) CWR located? (3) Can the area of a GR be limited in size to simplify its establishment while still conserving a large number of CWR species? (4) Do CWR hotspots tend to be located in protected areas?

## Materials and methods

### Study area

The analyses were conducted in Germany. Northern Germany is rather flat, whereas central and southern Germany exhibit a diverse relief, with hills and mountain ranges reaching elevations of up to 1,500 m, while mountains of an altitude of up to 2,962 m exist in the Bavarian Alps. The climate is temperate, with regions in the southwest generally experiencing warmer conditions, and those in the east are characterised by greater aridity. Germany can be divided into eight biogeographic regions (following Finck et al. 2017). The biogeographic regions are divided into Atlantic, continental, Alpine, and marine areas. We omitted the marine areas as no priority CWR occur there.

### Target species

At the initiation of the GR network for CWR hotspots, we aim to conserve a maximum of diverse priority CWR species, ideally by maintaining several populations. The National Inventory of Plant Genetic Resources (PGRDEU, BLE 2019) and especially the national list of priority CWR (BEKO 2019; Bönisch and Thormann 2021) were used as the starting point for this study. A digital list of all vascular plants of Germany was provided by the Federal Agency for Nature Conservation of Germany (BfN) for which Metzing (2020) assigned CWR and priority CWR status (BEKO 2019; BLE 2019) to the taxa (Metzing et al. 2018) using the German vascular plant list (Buttler et al. 2018) for taxonomic alignment. The resulting CWR LIST (Online Resource SI1-Table 1; Metzing 2019, pers. communication) contained 3,398 taxa. Of these, 473 were priority CWR (Table 1). Many taxa in the list were subspecies, segregates of species aggregates, etc. Using data at the lowest taxonomic level would have led to a more frequent selection of hotspots with many microspecies and subspecies. With regard to the management of the targeted GR hotspots, it must be taken into account that the delineation of these taxonomic levels necessitates expertise. It is improbable that such experts could be generally hired for field surveys. Therefore, taxa groups, which are split into microspecies and subspecies (e.g. *Festuca ovina* agg. and *Rubius* sections), should be subsumed and analysed as a section or aggregate. To ensure an unambiguous counting of species (or in rare cases section or an aggregate) and because different synonymous names were used in the datasets, the CWR LIST was linked to the list of vascular plants of Germany (Buttler et al. 2018) via the genus and species name. Consequently, each taxon was assigned a unique Name ID. Finally, taxa with the same ID were considered one entity, which resulted in 1,696 CWR species, including 111 priority CWR (Online Resource SI1-Table 2). In this work, plant nomenclature is based on the CWR LIST.

**Table 1:** Overview of priority CWR in Germany

***Achillea*** *millefolium* L.^*^, *Ach. ptarmica* L. ***Allium*** *angulosum* L., *A*. *carinatum* L., *A*. *lusitanicum* Lam., *A*. *schoenoprasum* L., *A*. *scorodoprasum* L., *A*. *sphaerocephalon* L. ^*^, *A*. *strictum* Schrad., *A*. *suaveolens* Jacq., *A*. *ursinum* L.^*^, *A*. *victorialis* L. ***Apium*** *graveolens* L. ***Arnica*** *montana* L. ***Arum*** *maculatum* L. ***Asparagus*** *officinalis* L.^*^ ***Atriplex*** *calotheca* (Rafn) Fr. ***Avena*** *fatua* L. ***Beta*** *vulgaris* subsp. *maritima* (L.) Arcang. ***Brassica*** spp. ***Carex*** *randalpina* B. Walln., *C*. *arenaria* L., *C*. *pseudobrizoides* Clavaud, *C*. *brizoides* L., *C*. *trinervis* Degl. ***Carum*** *carvi* L. ***Cochlearia*** *anglica* L. ***Crataegus*** *laevigata* (Poir.) DC. ***Dactylis*** *glomerata* L.^*^ ***Daucus*** *carota* L.^*^ ***Deschampsia*** *wibeliana* (Sond.) Parl., *D*. *setacea* (Huds.) Hack.	***Festuca*** *arundinacea* Schreb.^*^, *F*. *heterophylla* Lam., *F*. *ovina* agg., *F*. *pratensis* Huds., *F*. *nigrescens* Lam.^*^, *F*. *rubra* L.^*^, *F*. *trichophylla* (Gaudin) K. Richt.^*^ ***Fragaria*** spp.^*^ ***Helosciadium*** spp. ***Hippophae*** *rhamnoides* L.^*^ ***Hordeum*** *marinum* Huds., *H*. *murinum* L.^*^, *H*. *secalinum* Schreb. ***Humulus*** *lupulus* L. ***Hypericum*** *elodes* L., *H*. *hirsutum* L., *H*. *humifusum* L., *H*. *perforatum* L. ^*^ ***Isatis*** *tinctoria* L.^*^ ***Juncus*** *anceps* Laharpe ***Lactuca*** spp.^*^ ***Lolium*** *perenne* L., *L*. *temulentum* L. ***Malus*** *sylvestris* (L.) Mill. ***Medicago*** *falcata* L. ***Mentha*** *aquatica* L., *M*. *arvensis* L., *M*. *pulegium* L., *M*. *longifolia* (L.) Huds. ***Oenanthe*** *conioides* Lange ***Origanum*** *vulgare* L. ^*^ ***Phleum*** *pratense* L. ***Poa*** *chaixii* Vill., *P*. *pratensis* L.	***Prunus*** *avium* (L.) L., *P*. *fruticosa* Pall., *P*. *mahaleb* L., *P*. *spinosa* agg. ***Pyrus*** *pyraster* (L.) Burgsd. ***Ribes*** *alpinum* L., *R*. *nigrum* L., *R*. *petraeum* Wulfen, *R*. *spicatum* E. Robson^*^, *R*. *uva*-*crispa* L. ***Rosa*** *arvensis* Huds., *R*. *sherardii* Davies ***Rubus*** *chamaemorus* L., *R*. *saxatilis* L., *R*. *idaeus* L., *R*. *caesius* L., *R*. sect. *Rubus* ***Sinapis*** *arvensis* L.^*^ ***Thymus*** *pulegioides* L.^*^, *Th*. *praecox* Opiz^*^, *Th*. *serpyllum* L.^*^ ***Trifolium*** *arvense* L.^*^, *T*. *campestre* Schreb., *T*. *ornithopodioides* L., *T*. *pratense* L.^*^, *T*. *repens* L.^*^ ***Urtica*** *dioica* L. ***Valeriana*** *officinalis* L. ***Valerianella*** spp.^*^ ***Vicia*** *cracca* L., *V*. *tenuifolia* Roth^*^, *V*. *lathyroides* L., *V*. *angustifolia* L., *V*. *sepium* L., *V*. *tetrasperma* (L.) Schreb. ***Vitis*** *gmelinii* Buttler

^*^ incl. subspecies

### Data collection and preparation

Data available on the distribution of CWR occurrences were compiled to identify CWR hotspots. Data were provided by the nature conservation authorities of 14 federal states, which collected the data, e.g. for the inventory of protected areas, habitats, environmental impact assessments, or for specific species surveys. Some datasets only contained observations on certain species (e.g. priority CWR, regional target species), on habitat types with typically many priority CWR, or on certain counties. Some datasets lacked observations on sensitive species (i.e. rare or relict taxa). The Global Biodiversity Information Facility (GBIF) was also searched for CWR data with a maximum uncertainty of one kilometre. It provided access to standardised, nationwide presence data of the Citizen Science (CS) portals i.e. *Naturgucker.de*, *iNaturalist.org*, *Oberservation.org* as well as from several projects (GBIF.org 2020a, 2020b, 2020c, 2020d, 2020e). The CS data at GBIF are supposed to have been checked for plausibility by users or experts (Kaufmann and Lindner 2021). The project GE-Sell (Bönisch et al. 2020) and the platform “Vegetweb - Vegetation Portal for Germany” (Jansen et al. 2015; Online Resource SI2-Annex 1) provided additional observations.

The datasets analysed (Online Resource SI2-Annex 2) were based on different survey methods, and had different aims (e.g. surveys of biotopes or individual plants, vegetation inventories). The raw geodata were filtered with ArcGIS Pro v. 2.8.3 (ESRI Inc.) (Online Resource SI2-Annex 3). Geometries consisting of lines or polygons were reduced to a central point. Thus, each population of a species that had been observed was spatially represented by one point coordinate. Finally, PostgreSQL (version 15.2) with PostGIS extension (version 3.3.2) via PGAdmin4 (version 6.21) and QGIS (version 3.22.16) were used to merge all datasets to a shapefile for the subsequent hotspot analysis (Online Resource SI3).

### Hotspot analysis

Hotspots were identified based on the number of CWR and priority CWR in individual grid cells of approximately 3 × 3 km each (45,370 grid cells in Germany, Online resource SI4). Each grid cell is equivalent to a sixteenth of a sheet of the German 1:25,000 topographic map (BKG 2020). The (priority) CWR were counted per grid cell by intersecting the shape of the occurrence points with the grid. This assigned the local species inventory and resulting species number to each grid cell.

In addition, the number of priority CWR typical for the vegetation types (VT) nitrophilous herbaceous perennial vegetation (VT7), semi-wet meadows and pasturages (VT16), and dry and semi-dry grasslands (VT18) was calculated for each grid cell. These are three vegetation types (Korneck and Sukopp 1988) with which priority CWR are mainly associated (Online Resource SI1-Table 1). The vegetation types represent different species communities and their use serves as a proxy for site heterogeneity. This is intended to increase the number of target species covered by a GR network.

### Identification of a pool of potential GRs

In each biogeographical region, grid cells with the highest number of species were chosen to form a nationwide pool of potential GRs of approximately 250 cells for each of the four categories (1,000 cells in total): priority CWR (1) total overall vegetation types, (2) typical for VT7, (3) VT16, and (4) for VT18 (Online Resource SI1-Table 3). The number of cells to be selected per biogeographic region and category was proportional to the size of the biogeographic region. In this way, sites were selected containing populations adapted to different large-scale ecological characteristics.

### Refinement of the pool of potential GRs

In order to designate a pilot set of GRs, the pool of potential GRs was reduced to a number that can be coordinated by a national office. Therefore, approximately half of the grid cells with the highest number of priority CWR and the ones typical for the three vegetation types of each biogeographic region were selected from the 1,000 potential GR pool (Online Resource SI1-Table 3). In regions, in which potential GRs clustered, the cell with the highest total number of priority CWR was selected. However, cells were excluded or replaced by nearby cells if further information indicated that they were unsuitable for the establishment of GRs (e.g. were part of a park or military area, Online Resource SI1-Table 3). This allows for flexibility and takes the practical challenges of GR establishment into account.

### Defining GRs, on-site surveys, and data comparison

We wanted to investigate if it is possible to limit the size of GRs to make it easier to establish them, while still conserving a high number of (priority) CWR. For 27 hotspot grid cells (Online Resource SI1-Table 3) a set of hotspot biotopes was identified as GR candidate areas using ArcGIS Pro. Firstly, observation data in a grid cell were used to determine the number of priority CWR in each georeferenced biotope with a size smaller than 5 ha. In the second step, the biotope with the highest number of species in a grid cell was identified. Then, this site was supplemented by additional small biotopes until the area had a total size of approximately 5 ha. Therefore, biotopes with the highest number of complementary priority CWR were chosen. If several biotopes were suitable, preference was given to biotopes of different habitat types to increase the variety of growing conditions. To facilitate the future management of GR sites, the following additional criteria were applied to select biotopes: (1) the patches of which a GR candidate consists should preferably not be spread over different counties and their distance from each other should be less than one kilometre, (2) the site and its CWR populations should be suitable for the in situ conservation (e.g. no horticultural use) and the probability that seed mixtures have been used should be low, and (3) the number of landowners or plots should be low (preferable not more than ten). Thus, patches that contained the highest number of priority CWR and had a total size of ca. 5 ha were selected. This size is less than 1% of a grid cell and was predefined as mapping was feasible given the available resources and because it represents the minimum size for a nature reserve in Germany (BfN 2024a).

We investigated the priority CWR inventory from database records and tested whether the number of priority CWR in GR candidates increases with the size of the sites and their overlap with protected areas. Therefore, the proportion of Special Areas of Conservation (SAC), as defined in the European Union’s Habitats Directive (92/43/EEC; EEC (1992; EEA 2022), and nature reserves (IUCN category IV; BfN 2024b) were analysed. The 27 GR candidates were visited and the plant species occurring were recorded between May and September of 2021, 2022, or 2024. We then compared the list of recorded priority CWR with the data used to select the sites.

## Results

### Database setup

The plant observation data from different sources (e.g. conservation authorities, public databases) were filtered. The resulting database consisted of 100 datasets (including polygon (*n* = 37), line (8), and point (48) geodatasets; Online Resource SI2-Annex 2) with approximately 30 million species observations. Of the observations 80% concerned CWR, of which 14% referred to priority CWR (Table 2). The database contained records for 1,661 CWR, 110 of these were priority CWR.

**Table 2:** Number of observations of vascular plants, CWR, and priority CWR used

Number	Polygons	Lines	Points
Vascular plants	20,918,798	555,912	8,440,724
CWR	16,787,454	467,103	6,532,539
Priority CWR	2,305,823	64,361	925,103

### Species number and hotspots

**Fig. 1:**
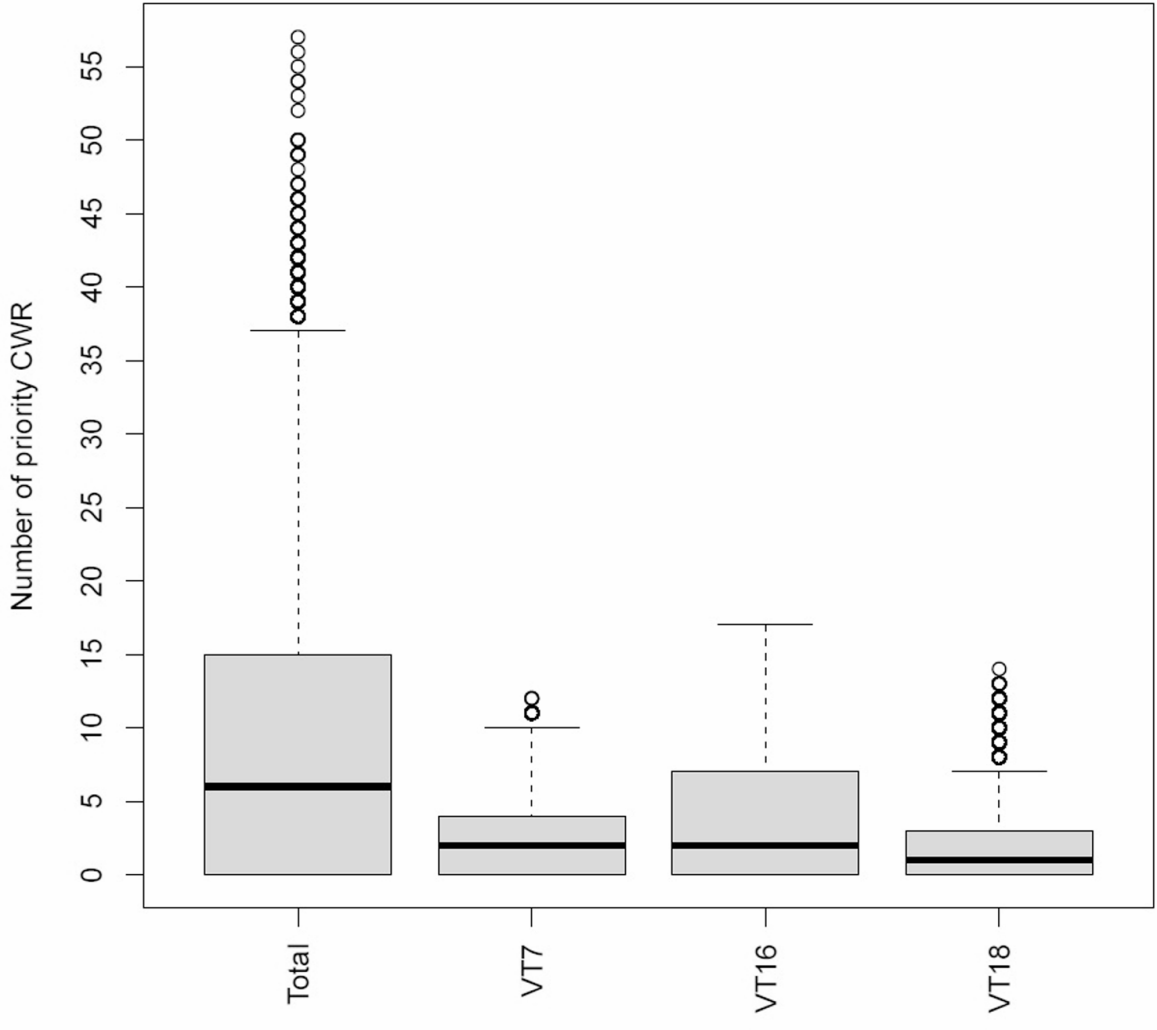
Boxplots showing the number of total priority CWR, and priority CWR typical for nitrophilous herbaceous perennial vegetation (VT7), semi-wet meadows and pasturages (VT16), and dry and semi-dry grasslands (VT18) per grid cell

**Fig. 2:**
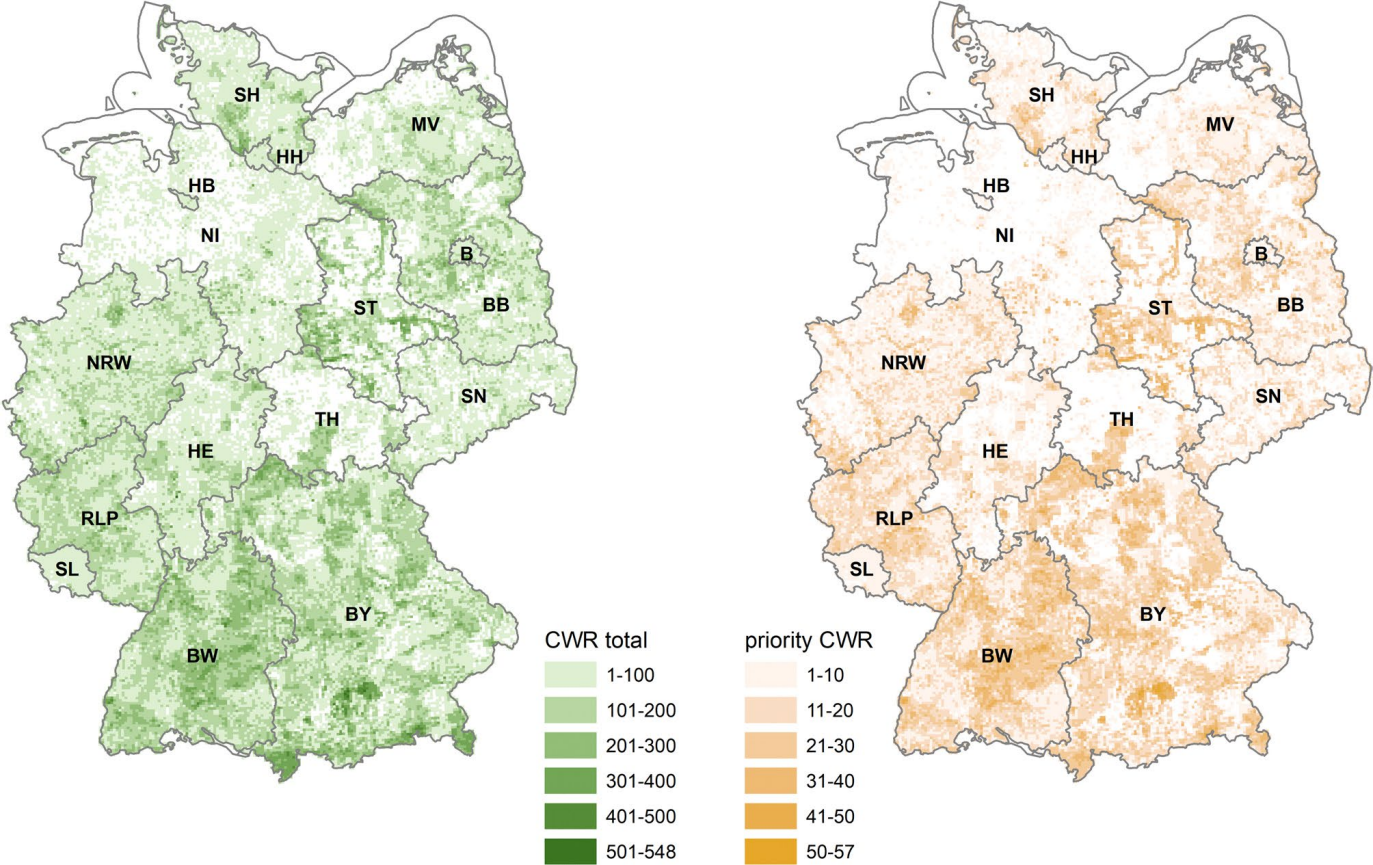
Comparison of Taxon richness: Number of CWR (left) and priority CWR (right) per grid cell (approximately 3 × 3 km). Abbreviations for the names of the federal states are explained in Online Resource SI1-Table 3. Federal state borders by © GeoBasis-DE/BKG 2016

We assigned the local species inventory and the resulting number of (priority) CWR species to each grid cell by intersecting the shape of occurrence with the grid. We also focused on three vegetation types with which many priority CWR are associated (Online resource SI4). At least 200 and up to 548 CWR were present in 9% of the cells. More than 30 priority CWR were detected in 3% of the cells and 21 to 30 in 12% (Online Resource SI1-Table 4). The count also showed that at least ten priority CWR typical for nitrophilous herbaceous perennial vegetation (VT7), semi-wet meadows and pasturages (VT16), and dry and semi-dry grasslands (VT18) occurred in 0.8%, 15%, and 0.6% of the cells, respectively. The highest number of priority CWR in a cell was 12 for VT7, 17 for VT16, and 14 for VT18 (Fig. 1, Online Resource SI1-Table 4). In 20% and 31% of cells, respectively, there was no information on the occurrence of CWR and priority CWR. These cells were scattered across Germany, but clustered in Lower Saxony (NI) and regions of Mecklenburg-Western Pomerania (MV), Saxony (SN), and Thuringia (TH), for which less observation data was available (Fig. 2).

On average, 11.4% of the CWR in a cell were priority species. The number of priority CWR per grid cell was strongly correlated with the total number of CWR (*r* = 0.96). The 58 grid cells with the highest number of CWR (more than 400 CWR) were mainly located in continental and Alpine regions in southern Bavaria (BY, 39 cells) and, more rarely, in the central and southern federal states Baden-Wuerttemberg (BW), Hesse (HE), North Rhine-Westphalia (NRW), and in Saxony-Anhalt (ST) along the river Elbe (Fig. 2). Grid cells with 301 to 400 CWR occurred as clusters in BY, BW and ST and as single cells in Berlin (B), Brandenburg (BB), NI, Rhineland-Palatinate (RLP) and Schleswig-Holstein (SH). Only a few CWR were reported for the federal states Bremen (HB), Hamburg (HH), and MV (Fig. 2).

All grid cells with more than 50 priority CWR and 31% of the cells with 41 to 50 species were located near Munich (BY). In contrast to the many cells with high CWR numbers in the Alps of BY, there were only four grid cells with more than 40 priority CWR in this biogeographic region. Several cells with 41 to 50 priority CWR occurred in BW, ST, and other regions of BY and sporadically in HE, NI, RLP, SH, and TH. Clusters of priority CWR with 31 to 40 taxa were found in BW, BY, and ST. In the remaining federal states, there were no or only very few grid cells with 31 to 40 priority CWR (e.g. HB, HH, and SL) (Fig. 2).

Grid cells with the highest number of priority CWR typical for VT7 and VT16 were most common in southern Germany and ST. VT16 priority CWR occurred in large clusters in the uplands of the Rhön (BY), the Thuringian Forest (TH), and the Eifel, or were distributed over Germany. In total 106 grid cells had at least 11 typical priority CWR for VT18. The cells were scattered or formed small clusters (i.e. several cells were located in close proximity) over different biogeographic regions (Online Resource SI2-Annex 4). Overall, cells with a high number of species were mainly located in the southern regions of Germany.

### Identification and refinement of the pool of potential GRs

**Fig. 3:**
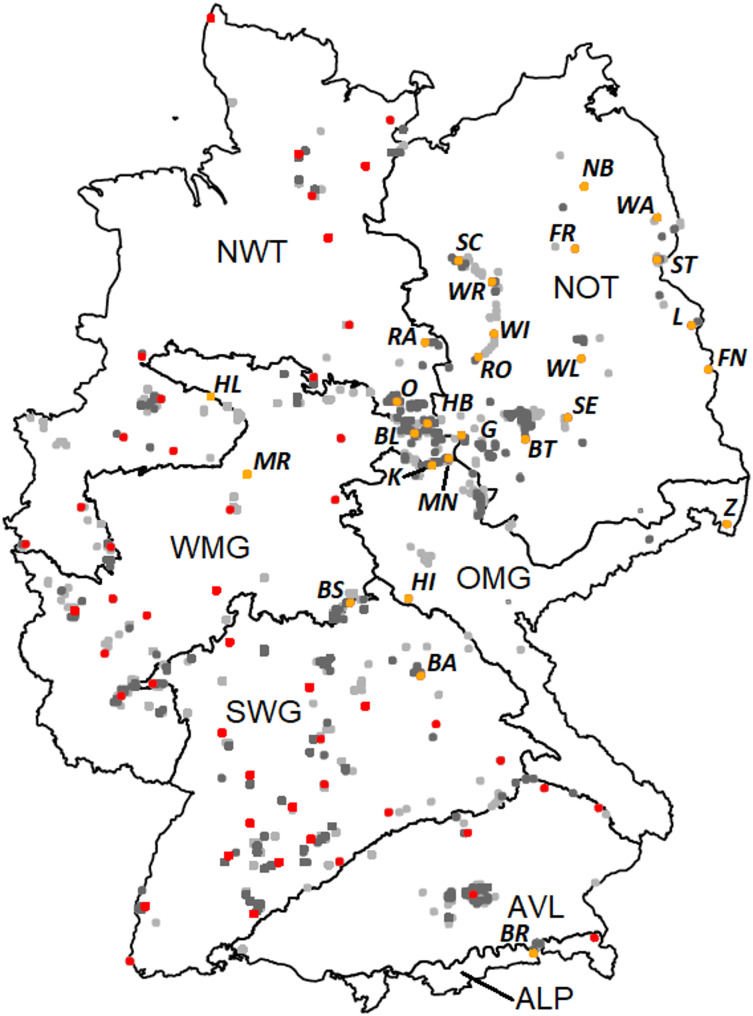
Map showing the 1,000 hotspot grid cells with the highest number of priority CWR across the Atlantic (North-West Lowlands (NWT)), continental (North-East Lowlands (NOT), Western Uplands (WMG), South-Western Uplands (SWG), Eastern Uplands (OMG), Prealps (AVL)), and Alpine (ALP) biogeographic regions (Finck et al. 2017) of Germany. 414 of the 1,000 cells are identical because the map shows the results of the analyses for priority CWR overall and for the four different vegetation types as an overlap. Red and Orange: 76 hotspot cells as a pilot set for a GR network, Orange: 27 hotspot cells with a delineated GR candidate site, where on-site visits have been conducted. Grey: the further hotspot grid cells of the pool of potential GR. Dark grey: approximately half of the 1,000 hotspot cells with the highest number of priority CWR in total and typical for the three vegetation types of each biogeographic region. Visited GR candidates are abbreviated using italic capital letters (Online Resource SI1-Table 3)

Grid cells were identified, which had the highest number of priority CWR overall (253 cells), and those typical for VT7 (247), VT16 (247), and VT18 (253). These 1,000 grid cells, distributed across the biogeographical regions, were selected as potential GRs (Online Resource SI1-Table 3 & SI2-Annex 5). Some hotspot grid cells occurred in clusters (e.g. in BY and ST). The pool of 1,000 hotspot grid cells was reduced to 520 to designate a pilot set of GRs. Of these cells, 331 had unique locations (Fig. 3), as cells were identical, when the analyses for priority CWR overall and for the three different vegetation types resulted in the same cells returned as their hotspots cells. For cell clusters, the cell with the highest number of priority CWR was selected. Several cells were unsuitable for GR establishments (e.g. they were part of military areas or parks) and, therefore, excluded or replaced by hotspot cells nearby. These cells in the vicinity were used, if information for assessing its potential as a GR was available, e.g., contacts were established with local stakeholders. The rationale behind this is that a slightly smaller number of CWR species is preferable to failing to establish a GR at this location. Contact with and approval from local stakeholders frequently act as the primary impediment to the establishment of an otherwise suitable GR candidate.

Overall, 76 hotspot grid cells were selected to form the pilot set to establish a GR network (Fig. 3, Online Resource SI1-Table 3). These cells were distributed across all federal states except for SL, B, HB, and HH. Each grid cell contained between 160 and 527 CWR (of which 24 to 57 were priority CWR; Online Resource SI1-Table 3). Overall, 1,245 CWR (73% of the total 1,696 species) occurred in the 76 hotspot grid cells. Of these, 94 species were priority CWR (85% of the total 111). Some priority CWR, e.g. *Dactylis glomerata* agg., *Hypericum perforatum* and *Urtica dioica* were very common and recorded in all cells. However, 21 extremely rare species (e.g. the priority CWR *Deschampsia setacea* and *Ribes petraeum*) and a further 82 very rare species (e.g. the priority CWR *Hypericum elodes* and *Juncus anceps*) also occurred in the set of cells. In addition, 15 of the occurring CWR were considered to be threatened with extinction in Germany (Metzing et al. 2018).

### Assessment of GR candidates

We selected 27 hotspot grid cells (Fig. 3, Online Resource SI5) of the 76 and delineated putative GR sites based on available biotope information. The area of the candidate sites ranged from 1.7 to 6.1 ha. They were distributed across seven federal states and six biogeographic regions. Based on biotope data, the GR sites often consisted of grasslands, which differed in nutrient level and soil moisture. In 12 GR candidates, the grassland included scattered woody vegetation (e.g. orchards or copses; Online Resource SI1-Table 3).

**Fig. 4:**
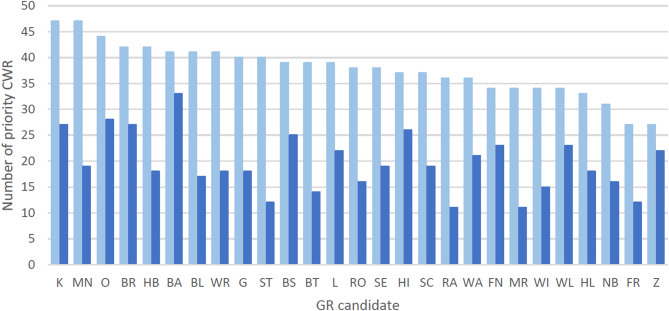
Number of priority CWR based on observation data in the genetic reserve (GR) candidates (dark blue) and the respective entire grid cells (light blue). Explanations of the abbreviations of GR candidates can be found in Fig. 3 and in Online Resource SI1-Table 3

Based on the observation data of the years 2000 to 2020 (Online Resource SI2-Annex 2), 11 to 33 priority CWR had been recorded in each GR candidate site. These represent 30 to 81% (on average 52%) of the priority CWR of the entire respective grid cell (Fig. 4). In total, the candidates contained 62 of the 111 priority CWR. Thus, these 27 GR sites could conserve 56% of the priority CWR. 36 of the priority CWR occurred in at least five GR candidates (Online Resource SI1-Table 5). The common priority CWR *Achillea millefolium* agg. and *Dactylis glomerata* agg. had occurrences in 24 sites. Rare priority CWR (Metzing et al. 2018) were recorded in at least one site: *Allium angulosum* (3 sites), *A. schoenoprasum* (1*)*,* Festuca pallens* (1), *F. psammophila* (2) and *Thymus praecox* (3). Moreover, the following priority CWR for which Germany has global conservation responsibility (Ludwig et al. 2007) had occurrences in at least one GR candidate site: *Arnica montana* (1), *Crataegus laevigata* (6*)*, *Festuca brevipila* (8) and *Vicia lathyroides* (3). Although only one GR candidate had been selected in each of the biogeographical regions North-West Lowlands, the Alps, and the South-Western Uplands, these could already maintain 19, 28, and 32 priority CWR with one population. For the Eastern and Western Uplands, three and four GR candidates were chosen, respectively. They contained 37 and 35 priority CWR, mostly with one population each. 17 of the 27 GR candidates were assigned to the North-East Lowlands. In these, 56 priority CWR could be preserved, half of them with at least three populations (Online Resource SI1-Table 5).

**Fig. 5:**
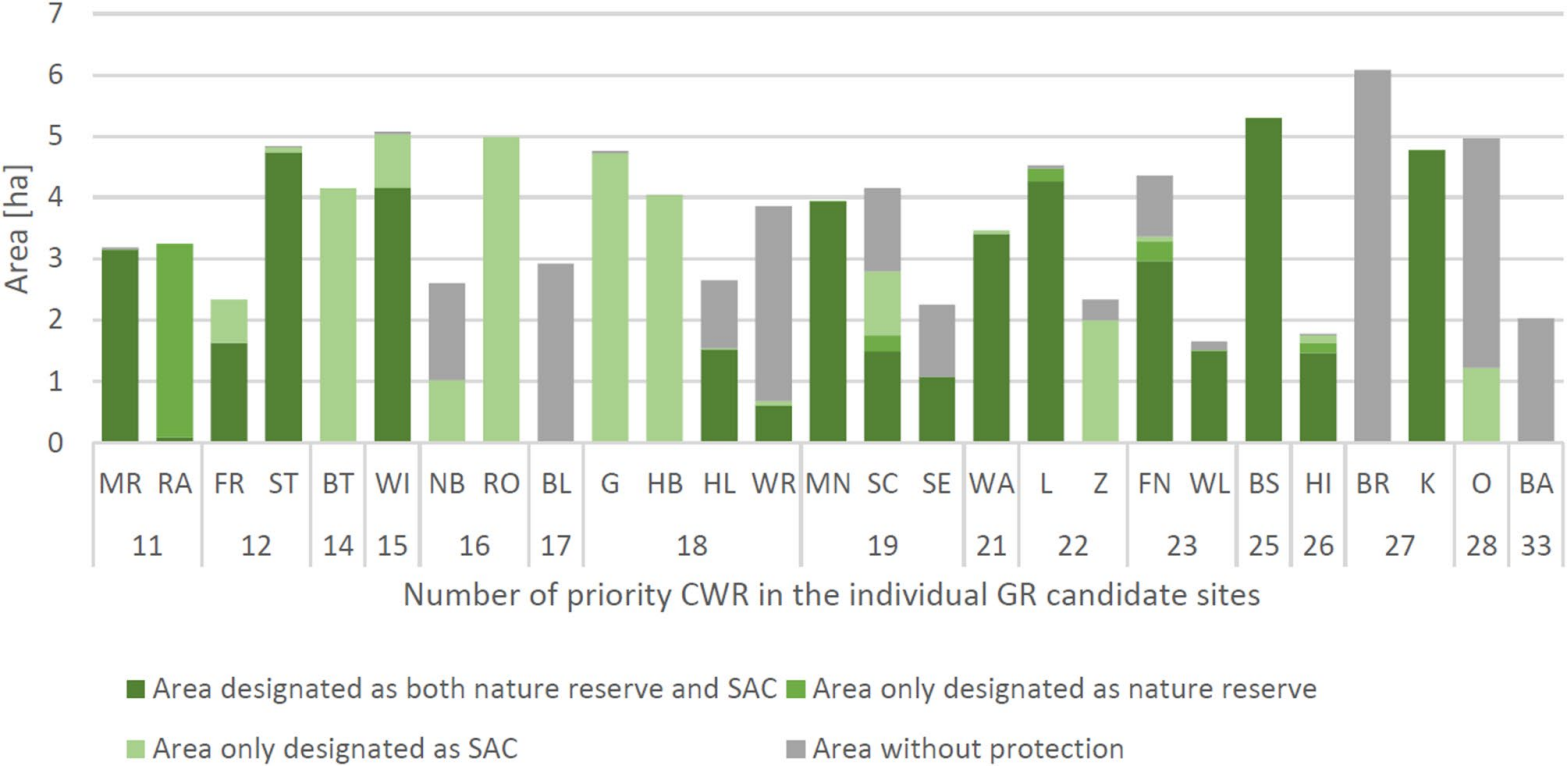
Overview of the size of the 27 genetic reserve (GR) candidates and the overlap with a protected area. Special Areas of Conservation (SAC) are protected areas of the Habitat Directive (EEC 1992). The number of priority CWR is given below. GR candidates are abbreviated as in Online Resource SI1-Table 3

An analysis was conducted of the size of the GR candidate sites, their overlap with protected areas, and the number of priority CWR. The data showed that for the majority of the 27 GR candidates, a large proportion of the site was located within a nature reserve or a Special Area of Conservation (SAC). Only three GR candidates were located entirely outside of a protected area and for three candidates the proportion of protected area was less than 50%. Neither the size of the candidate sites, which ranged from 1.7 to 6.1 ha, nor the overlap of the candidate sites with a protected area were reliable indicators of the number of priority CWR at a site (Fig. 5).

**Fig. 6:**
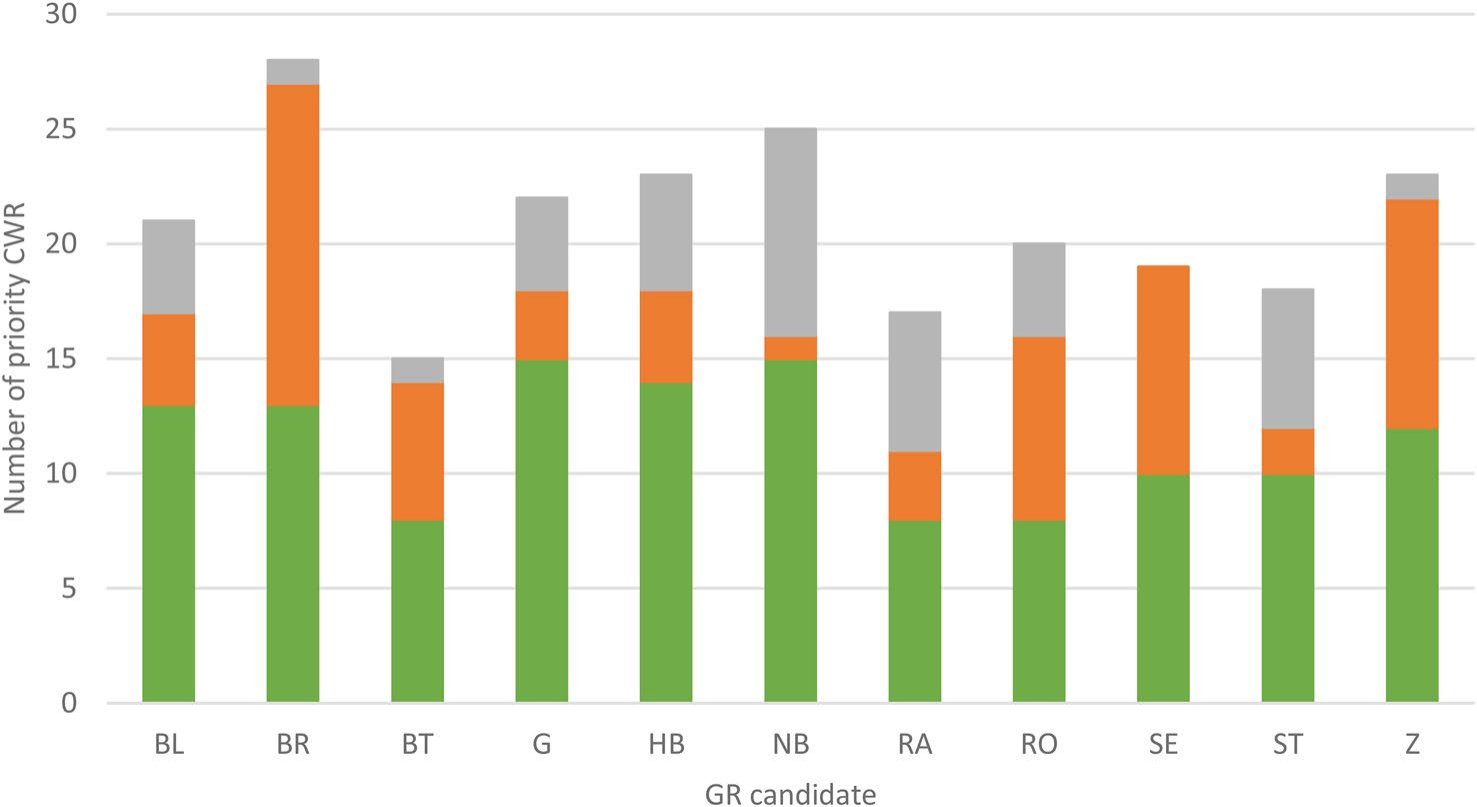
Number of priority CWR verified (green), not verified (orange), and newly surveyed (grey) at each of the 11 fully surveyed genetic reserve (GR) candidate sites. The location of GR candidates is displayed in Fig. 3

On-site surveys were planned for 27 GR candidates, but only eleven could be adequately assessed. Sixteen of the delineated GR sites could not be fully investigated due to ongoing land use or restrictions of access. For example, it was difficult to identify the CWR in sites, that were mown or grazed, or the owners did not give their consent for the survey (Online Resource SI1-Table 3). For the remaining eleven GR candidates, 30–94% of the taxa listed in the database could be confirmed (mean 68%; Fig. 6; Online Resource SI1-Table 3). However, at some sites, priority CWR were observed that had not been listed, most frequently *Lactuca serriola*, *Trifolium repens*, and *Vica angustifolia*. Each of these was newly recorded at four sites.

## Discussion

This study identified CWR hotspots with the potential to become sites for a GR network in Germany. Therefore, CWR observation data were collected on a national scale from various sources and a hotspot analysis was performed. Field surveys for recording the current CWR inventory were conducted for 27 sites.

### Database and CWR hotspot identification

The database for the hotspot identification was compiled using data provided by 14 federal states of Germany, from GBIF, and from research projects. Our study identified grid cells with the highest number of CWR and priority CWR mainly in southern Germany. There they cover larger contiguous areas. The centres with high CWR numbers reflect the distribution of phytodiversity hotspots in Germany, which are mainly due to climate, geomorphology, and migration corridors (e.g. Schmitt and Haeupler 2009). Overall, the centres of high CWR and priority CWR density are more or less identical across Germany (Fig. 2). This indicates that priority CWR hotspots can be used as proxies for hotspots of CWR in general. It can therefore be concluded that future identifications of further GR could be based on data on priority CWR. However, this may not apply to rare CWR or CWR with specific habitat requirements, such as the wild relatives of celery (Frese et al. 2018).

Assessing species diversity on a grid cell basis is a common method for hotspot analyses (e.g. Cañadas et al. 2014; Castilho Silva et al. 2024; Fielder et al. 2015; Khaki Mponya et al. 2021; Schouten et al. 2010; Sussman et al. 2019). This method allows the entire area of Germany to be considered in a standardised procedure when identifying hotspots. The number of species per grid cell was based on harmonised location information (site coordinate or biotope reduced to a centroid). For polygons, only observations whose location uncertainty was at a maximum equal to that of the grid cell were considered (cf. Cañadas et al. 2014). The use of data with a higher spatial resolution would drastically decrease the number of suitable observation data and unintentionally fragment larger biotope complexes so that existing hotspots might not be recognised. In contrast, using a lower resolution grid would hamper the identification of GR candidates of practicable size. The selected resolution allowed the inclusion of a large part of the observation data (cf. Schouten et al. 2010).

However, the comparison of the number of CWR was affected by several factors: [1] The quality of the data varied considerably among regions due to differences in data collection methods, inventory completion, and area coverage (Jansen et al. 2020; Borgmann et al. 2023). For example, in TH, extensive data was only available for some districts, and in SL and SN, the number of species per grid cell was generally lower. However, in SL, observations were only available for certain priority CWR, and in SN only for certain types of biotopes. The low number of species in northern Germany (Fig. 2) has probably been the result of only partially collected data and poor data availability (especially in NI, MV, and TH). Cells with a low number can also be attributed to the effects of intensive agricultural practices, large field sizes, and reduced grassland habitats (Leuschner et al. 2013; Hötker and Leuschner 2014; Krause et al. 2014), as grasslands, hedges, field margins, and road verges are habitats where CWR frequently occur (Jarvis et al. 2015). The collectivisation of farms and the centrally planned economy in the former German Democratic Republic resulted in agricultural structures tending to be larger and more widespread monocultures in East Germany compared to those in the West. As a result of this standardisation of land use, there has been a less local variety of habitats for decades. [2] The comparability of the number of species per grid cell was reduced by variation in the size of the grid cells: (i) For cells on the federal border, species observations are only available for part of the area located in Germany. Hence, the species richness may be lower in comparison to other grids, due to the reduced area that could be considered. This is affecting 3.9% percent of the grid cells. (ii) The size of cells increases slightly along the north-south gradient due to the earth’s curvature and species are counted in a larger area in the south. The smallest grid cell was 740 ha, and the largest was 877 ha. [3] For most datasets, no information was available on the extent to which a region was surveyed. It can be assumed that certain habitat types (e.g. forests) are underrepresented. Moreover, there are often CWR that are not consistently listed in the inventory. They include taxa that were not the focus of the data collection at a site, or which are easily overlooked, e.g. because they are very tiny or have a short flowering period, or which can be identified only by experts. To overcome these limitations, regularly repeated, comprehensive, and standardised nationwide biodiversity analyses are required. Ideally, the data would then be compiled in a single database. With the shift to technologies using remote sensing and artificial intelligence, this is more likely to happen in the future (e.g. Reddy 2021; Lausch et al. 2024).

Modelling approaches, e.g. species distribution models (Parviainen et al. 2009), have the potential to reduce the incompleteness and heterogeneity of datasets. However, they are associated with uncertainties (Jansen et al. 2020). Because for a large part of the study area high spatial resolution data and a high number of observations were available, the use of true spatially explicit observations was considered more suitable for this study. Overall, these data have proven suitable for the hotspot analysis.

### Selection of GR candidates

Hotspots are often referred to as the five or ten per cent of the most species-rich count units (e.g. grid cells) or as the areas with a specified minimum percentage of the total species pool (Sfenthourakis and Legakis 2001). However, these thresholds are subjective (Harvey et al. 2017). In this study, the ca. 250 hotspot cells selected for the priority CWR in total and for each of the three vegetation types, respectively, corresponded to the 0.6% of cells in Germany with the highest number of priority CWR. This pool of potential GRs was distributed over the entire area of Germany. The Alpine, the Atlantic (North-West Lowlands), and the continental biogeographic regions Prealps and Eastern Uplands contained areas without potential GRs (Fig. 3). The reasons therefore are that hotspots were concentrated in certain areas within a biogeographic region, i.e. hotspot cells clustered, or a cell represented a hotspot for several vegetation types, or for at least one vegetation type and for the priority CWR altogether. Areas without hotspots were also caused by a lack of data.

In total, we identified 76 hotspot cells with the highest number of priority CWR (also for the three selected vegetation types) and with no obvious obstacles to the establishment of a GR (e.g. unsuitable sites, such as military areas). These hotspot cells are more or less evenly distributed across the biogeographic regions (Fig. 3). In the case of clusters of hotspots, we selected the cell with the highest number of priority CWR from each cluster. The other cells of the cluster remained in the pool as an alternative if a GR cannot be established.

In the 76 sites, 85% of the priority CWR species (73% of the total CWR) occurred. The GR network would thus conserve more than two-thirds of the CWR species, which is the proportion proposed for effective CWR conservation (Maxted et al. 2007). Other studies suggest covering this area with fewer, but larger GR candidates. For instance, ten protected areas, including national parks were proposed by Khaki Mponya et al. (2021) and Fitzgerald et al. (2023) or 17 hotspot grid cells of 100 km² in Maxted et al. (2007). Our study is based on a grid cell of approximately 8 km² and GR candidates of approximately 5 ha. Due to these differences in size, a comparison is not straightforward. This study has opted for smaller areas as limiting the size of a GR, and therefore reducing the number of stakeholders, may simplify its establishment.

The number of priority CWR species in the network of potential GR was increased by the inclusion of sites with many priority CWR typical for three vegetation types. Furthermore, considering different biogeographic regions promotes the selection of populations, whose eco-geography differs. Due to the spatial scattering of the 76 potential GRs, the sites are generally located at a considerable geographic distance from each other which may limit gene flow. Thus, their populations could already differ genetically. For this reason, we did not exclude species from the selection of further hotspots if they were already represented in a potential GR. Therefore, multiple populations of a species will be represented in the GR network. This increases the intraspecific diversity covered (Maxted et al. 2012). Conserving several populations will preserve a higher proportion of their intraspecific diversity and rare alleles (Whitlock et al. 2016). Brown and Briggs (1991) posited that a minimum of five populations per taxon should be conserved in situ. Whitlock et al. (2016) estimated for eight widespread but declining plant species in Great Britain that by conserving three populations 70% of their genetic diversity could be preserved, as demanded by the CBD (2010). However, five to 16 populations per species are needed to maintain rare alleles to the same extent.

For 27 candidate sites, the size of the GR candidate was set to approximately 5 ha in a hotspot grid cell. The delineation of the GR area was based on biotopes and not on the spatial extent of populations. Therefore, a GR candidate site may not cover the entire population of each priority CWR. The advantages of this approach are: (1) Information on the spatial extent of a plant population, which is often not available, is not necessary. (2) Sites are determined before on-site surveys take place, allowing permits to be obtained. (3) The size of a GR candidate remains small, as it is not inflated due to populations with a large spatial extent.

It was shown that a high proportion of the priority CWR species of the hotspot grid cells occurred within the GR candidate sites, whose size was less than 1% of the grid cells. Limiting the size of a GR may simplify its establishment and outweigh a minor loss of species coverage by reducing the area. According to the database, more than half of the priority CWR species could be conserved with at least one population within the 27 GR candidate sites, 36 of those species with a minimum five populations (Online Ressource SI1-Table 5). Considering that further GR candidates will be added, our approach appears well-suited to cover at least five populations for the majority of priority CWR.

Our approach to identifying GRs differs from the complementarity analysis (CA), in which hotspots with complement species inventories are identified for different combinations of ecogeographic land characteristics in an iterative procedure to cover each taxon at least once (Maxted et al. 2007). As the target species inventory of the sites is interdependent, the GR identified through CA may cover most of the target species if all of the selected GR candidates are actually established. We did not conduct the CA, since a more flexible approach was required with regard to the practical implementation of GRs. Based on our experience (Bönisch et al. 2020), we consider it improbable that the establishment of all the selected GR candidates will be realised. For instance, some sites, such as parks or military sites, will turn out unsuitable because access is not permitted, e.g. due to unexploded ordnance at military training grounds. Another obstacle for GR implementation is that the procurement of owner data is subject to data protection requirements and is regulated differently in the 16 federal states of Germany. This can unfortunately result in a rather lengthy process. The setup and management of a GR may fail if there is no support from local stakeholders (Bönisch et al. 2020; Leibenath and Schröder 2023). Hence, private landowners, farmers, shepherds, or other stakeholders have to be convinced to support the GR. Often, awareness about the importance of conserving CWR must be raised. So far, their involvement is not being recognised financially in Germany and they often fear land use restrictions in the future. Thus, for the successful establishment of a GR network, it is crucial to identify such potential obstacles as early as possible.

The CA was also found to affect GR identification when different regional scales are considered (Rusanen et al. 2021), as the choice of GR candidates is interdependent. In the context of the present study, the necessity would arise for the analysis to be updated in cases where GR candidates cannot be established. The objective of the update would be to ensure that the coverage of target species is restored. Moreover, a discrepancy was identified between the species observation records obtained from databases and the results of recent field surveys. This also means that a CA based on database records or recent field surveys probably would return different results.

Our method allows for the replacement of GR candidates with candidates nearby, although their target species inventories may vary. However, the goal is the actual establishment of GR, and therefore a lower number of taxa in the GR is preferable rather than a complete failure to establish a GR in this region. Additionally, we used data and tools, which are well-known by the German nature conservation authorities in Germany. For this reason, our method can be easily understood and applied by these stakeholders. Both are important criteria for involving the government and voluntary nature conservation actors in the conservation of CWR.

Nevertheless, we recommend using the CA after the establishment of the first GRs. Species lists based on field surveys could be examined to identify priority CWR that are not covered with sufficient probability within the existing GR network. Further sites for these species could be selected from the pool of potential GRs or by using CA. If a species does not occur in the hotspots, a species-specific GR network can be set up (Frese et al. 2018; Mewis et al. 2024). Subsequent genetic analyses may be helpful to identify gaps in the conservation of the genetic diversity for certain species (Gradl et al. 2022; Durka et al. 2024).

### Comparison of records from on-site surveys and database entries

Once potential GR candidates had been identified, CWR species were recorded on-site and the inventories were compared to the database for eleven candidate sites. The recording was deemed necessary as database species observations were up to 20 years old. On average, two-thirds of the priority CWR could be confirmed. At ten sites, new CWR were detected (Fig. 6). The species that were not confirmed did either no longer occur on the site, were not recorded due to an absence of above-ground plant parts (e.g. in case of dormancy), were potentially overlooked, or the initial observation record may have been incorrect. However, it is unlikely that data errors are the main cause of the discrepancies, as obviously incorrect data were excluded (Online Resource SI2-Annex 3), only plausibility-checked CS data were used, and the data providers were reliable sources. It seems more probable that the discrepancy with the dataset can be attributed to a temporary non-appearance or an actual change in the species occurring. A permanent loss could be due to extinctions of populations due to demographic and environmental stochasticity, or as a result of changes in habitat conditions caused by changes in land use, other human interventions, or climate change. For example, it is likely that *Thymus pulegioides*, *Trifolium campestre*, and *Vicia cracca* disappeared because sites were less regularly grazed or mown. In contrast, *Lactuca serriola* was frequently newly recorded, probably because habitats had been recently disturbed. However, it is also possible that newly recorded species had been overlooked in previous surveys. Taxa which are difficult to observe are particularly likely to be overlooked. For instance, given that plots were mostly only visited once, species that are inconspicuous or have short flowering periods may have been missed. For 16 candidates, a complete on-site survey was not possible, as they were mown, grazed, or not fully accessible. Nonetheless, based on the database recordings, they persist as GR candidates that ought to be taken into consideration for prospective field surveys. Overall, a robust and comprehensive assessment of the species inventory requires several surveys of a site, which should be timed to avoid survey restrictions. However, in practice, this may conflict with the interests of the local stakeholders.

The changes in the set of species detected through the site visits indicate that similar to Special Areas of Conservation (SAC; EEC 1992), regular monitoring and appropriate measures for target species are necessary to track the dynamics in GRs and maintain or restore the favourable conservation status. Such monitoring should be conducted, e.g. every six years, in alignment with EU Directive 92/43/EEC (EEC 1992). The loss of species results in a reduction in the efficiency of CWR conservation, meaning that fewer CWR will be protected by a GR than the database suggests. If a population is not found for several years, prompt action could prevent the loss of a species. For example, it has been shown that populations can recover from the seed bank in the soil if favourable habitat conditions are restored in time (Mewis et al. 2024).

### GR candidates and overlap with protected areas

The analysis of the 27 GR candidate sites revealed that they were mostly located within a protected area. The size of a GR candidate and the extent of the overlap with a protected area were found to be no predictors for the number of priority CWR covered (Fig. 5). For example, the GR candidate BA contained the highest number of priority CWR (i.e. 33). However, it is approximately only two hectares in size and has no overlap with a protected area. This finding is in agreement with previous studies (Coetzee et al. 2014; Gray et al. 2016). It can therefore be concluded that GRs outside of protected areas may strongly contribute to conserving priority CWR. The experiences of the Wild Celery Network demonstrate that the management of GRs outside protected areas is possible if conservation guidance is provided to local stakeholders (Bönisch et al. 2020). This is in line with the recent introduction of other effective area-based conservation measures (OECM; CBD 2018), which also ensure the long-term conservation of biodiversity outside of protected areas. These measures contribute to the achievement of the internationally agreed conservation targets under the CBD (e.g. Kopsieker and Disselhoff 2024).

Presently, the conservation of CWR is predominantly achieved passively within protected areas (Kägi et al. 2023), which have been primarily set up to preserve rare or endangered ecosystems and species regardless of their importance for food and agriculture. Whether a CWR was endangered was not taken into account when the priority list for CWR conservation was established in Germany. Consequently, even common species are considered to be priority CWR. This is important as common species can have isolated populations carrying unique genetic diversity that should be conserved (Hargreaves et al. 2010). Moreover, it has been demonstrated that semi-wet and wet grasslands in northern and central Germany have experienced a decline in the abundance of previously common plant species, with a reduction of up to 90% (BfN 2015). Also, this study recorded a putative loss of taxa in the GR candidates, irrespective of the overlap with a protected area (Figs. 5 and 6). It has been argued that widespread but declining species might be more at risk of genetic erosion of unique alleles than rare species with stable distributions, because their sudden decline from a formerly widespread distribution may lead to inbreeding depression through loss of habitat connectivity (Habel and Schmitt 2012). Whitlock et al. (2016) suggested that more stringent conservation targets for genetic diversity might be necessary for these widespread species, especially if rare alleles are to be conserved. Therefore, the establishment of a GR in a protected area offers an additional benefit, namely the opportunity to combine the monitoring of the target species of the GR and of the protected area and thereby identify potential increases in vulnerability. Nevertheless, monitoring should always take place in GRs, whether in or outside protected areas. Monitoring might be supported by citizen science surveys, especially outside protected areas. This approach is already being promoted for GRs of wild celery (see https://gonature.de).

## Conclusions and outlook

Even though CWR are experiencing a decline in their natural habitats, the necessary conservation measures are not being implemented with sufficient urgency. This study aimed to identify CWR hotspots that are candidates for the establishment of a genetic reserve (GR) network, thereby facilitating the efficient conservation of CWR in Germany.

Based on the total number of priority CWR and the focus on three different vegetation types in grid cells of approximately 3 × 3 km, 331 unique hotspots were identified. We considered vegetation types and biogeographic regions in our analysis to increase site heterogeneity and thereby maximise the number of CWR and intraspecific diversity. Finally, 76 hotspot cells were selected as promising pilot GRs. They have the potential to conserve 73% of the CWR and 85% of the German priority CWR. For regions currently lacking observation data, the method developed can be used to identify hotspots with high numbers of CWR, as soon as new data become available. The use of species distribution models could be an alternative method for addressing the challenges of incomplete data.

We delineated GRs within a cumulative area of 5 ha. This size simplifies the establishment, monitoring, and management of GRs due to a limited number of stakeholders. We found, that in an area of that size, it is possible to conserve on average 52% of the priority CWR of the entire hotspot cell. Most GR candidates overlapped at least partially with protected areas. However, some candidate sites outside of such areas also contained high numbers of priority CWR and should be considered for GR establishment. The information we provide on CWR locations could also be used to facilitate the organisation of ex situ collections of CWR seeds.

While one-third of the priority CWR species records from past surveys could not be confirmed at eleven candidate GRs, new taxa were also found. This may indicate the effects of changes in climate and habitat use over the last 20 years, but could also be the result of extinctions of populations. However, variation in the species found could also result from limitations of past and current data collections. To ensure that CWR are adequately protected, they should be regularly monitored and, if necessary, managed. A stronger integration of CWR conservation into nature conservation is the key to efficient monitoring and management, especially for GRs in protected areas. This would offer synergies between the otherwise often conflicting goals of agricultural production and the protection of natural resources.

The approach presented here is characterised by the fact that it is flexible and can react to practical obstacles in the establishment of GR. These include e.g. bureaucratic hurdles (data protection) and the willingness of local stakeholders to participate. Our approach can identify multiple suitable sites for target regions. We advocate an approach that allows GR candidates to be replaced with nearby sites. This will conserve the intraspecific diversity of most target species within a given region, without the need for additional analysis.

To enhance the protection of CWR in Germany, several measures are required, which must be implemented at various levels. Effective CWR conservation requires a targeted communication strategy that results in widespread awareness of their critical importance, engaging everyone from local land users to policymakers. Increasing visibility of the topic and garnering support at all levels of government are essential. The long-term conservation and sustainable use of CWR depend on integrated action across scientific, political, and societal domains. This includes fostering close collaboration between agricultural, environmental, and research institutions to align multifunctional conservation and other socio-political goals in the cultural landscape. A nationwide database of species observations with a high spatial resolution is a prerequisite for systematic research on the conservation of species diversity and their effective implementation in practice. Such a resource would not only support future scientific studies but also help identify gaps in the conservation of genetic diversity for certain species. Moreover, the establishment of dedicated funding mechanisms for GRs would significantly enhance the willingness of local stakeholders to engage in and contribute to conservation efforts. Since 2024, the funding scheme “Gemeinschaftsaufgabe Agrarstruktur und Küstenschutz” (GAK), which is a German implementation instrument of the Common Agricultural Policy (CAP), co-financed by the EU, the federal government, and the federal states, supports the funding of the establishment and management of GRs. However, to mobilise these funds, the scheme needs to be implemented at the federal state level. Therefore, a targeted policy dialogue is urgently needed to promote the uptake of funding instruments within the individual federal states. Providing financial incentives for the conservation could mark a turning point in the preservation of CWR in Germany.

## Supplementary Information

Below is the link to the electronic supplementary material.

Supplementary Material 1

Supplementary Material 2

Supplementary Material 3

Supplementary Material 4

Supplementary Material 5

## Data Availability

The CS datasets supporting the conclusions of this article can be accessed via the URL given in the references. The dataset of the project GE-Sell is available from the corresponding author upon reasonable request. Datasets of the platform “Vegetweb - Vegetation Portal for Germany” and nature conservation authorities of federal states cannot be published due to usage restrictions imposed by the providers. Restrictions are due to the originators’ intellectual property rights and nature conservation interests, e.g. regarding rare species. However, the data may be shared by the providers (listed in Supplementary Information 2) or authors if authorised by the providers regarding a reasonable request. All data generated during this study are included in this published article or its supplementary information files.

## Declarations

### Nature conservation approval

If CWR hotspots were located in nature reserves, permission to access the area was obtained from the relevant authority prior to the survey.

## Abbreviations

ALP: Alpine biogeographic region
AVL: Biogeographic region Prealps
B: Federal state Berlin
BB: Federal state Brandenburg
BW: Federal state Baden-Wuerttemberg
BY: Federal state Bavaria
CA: Complementarity analysis
CS: Citizen Science
CWR: Crop wild relative
GR: Genetic reserve
HB: Federal state Bremen
HE: Federal state Hesse
HH: Federal state Hamburg
MV: Federal state Mecklenburg-Western Pomerania
NI: Federal state Lower Saxony
NRW: Federal state North Rhine-Westphalia
NOT: Biogeographic region North-East Lowlands
NWT: Biogeographic region North-West Lowlands
OMG: Biogeographic region Eastern Uplands
RLP: Federal state Rhineland-Palatinate
SAC: Special Areas of Conservation
SH: Federal state Schleswig-Holstein
SL: Federal state Saarland
SN: Federal state Saxony
ST: Federal state Saxony-Anhalt
SWG: Biogeographic region South-Western Uplands
TH: Federal state Thuringia
VT: Vegetation type
VT17: Nitrophilous herbaceous perennial vegetation
VT16: Semi-wet meadows and pasturages
VT18: Dry and semi-dry grasslands
WMG: Biogeographic region Western Uplands
