# Supplementary material for: Identification of hotspots of crop wild relatives in Germany to promote their in situ conservation in a network of genetic reserves
Source: Bot Stud. 2025 Sep 2;66:27. doi: 10.1186/s40529-025-00473-z (PMC12405085; doi:10.1186/s40529-025-00473-z)

Supplementary information 2

This document includes supplementary information on the article

"Identification of hotspots of crop wild relatives in Germany to promote their *in situ* conservation in a network of genetic reserves"

Maria Bönisch[1], Vera Senße[2], Thomas Engst[2], Alica Sander[2], Diethart Matthies[3], Eckhard Jedicke[4], Nadine Bernhardt[1]

[1]Julius Kühn-Institut (JKI) - Federal Research Centre for Cultivated Plants, Institute for Resistance Research and Stress Tolerance, Quedlinburg, Germany

[2]Anhalt University of Applied Sciences, Department of Agriculture, Ecotrophology and Landscape Development, Bernburg, Germany

[3] Philipps-Universität Marburg, Plant Ecology and Geobotany, Marburg, Germany

[4] Hochschule Geisenheim University, Department of Landscape Planning & Nature Conservation, Geisenheim, Germany

Corresponding authors: Maria Bönisch (maria.boenisch@julius-kuehn.de), Nadine Bernhardt (nadine.bernhardt@julius-kuehn.de)

<u>Annex 1:</u> List of publications, assessed via Vegetweb – Vegetationsportal für Deutschland (https://www.vegetweb.de):

Abs, C. (2002): Lebensfähigkeitsanalyse der gefährdeten Quellflurarten Cochlearia bavarica VOGT und Cochlearia pyrenaica DC

Adler, A. (2001): Kronwald

Altenfelder, S. et al. (2014): Effects of water regime and agricultural land use on diversity and species composition of vascular plants inhabiting temporary ponds in northeastern Germany

Anonymus (2016)

Barnick, C. et al. (2017): Erfassung und Charakterisierung der Vegetationseinheiten auf den Fernen und Murchiner Wiesen im Jahr 2017 nach 10 Jahren Pflegemahd

Bässler, C. (2006): Höhentransekte im Nationalpark Bayerischer Wald

Becker, Th. (2005): Luzula divulgata Kirschner (Schlanke Hainsimse)? Verbreitung, Vergesellschaftung und Standort einer Art xerothermer Eichenwälder in Mitteldeutschland

Beer, A. et al. (2005): Vegetationskundliche Untersuchungen rezent streugenutzter Kiefernwälder auf Binnendünen des niederbayerischen Tertiärhügellandes

Beitz, A. (2004): Vegetations- und Standortswandel im NSG "Birkbuschwiesen" bei Neubrandenburg

Berg, Ch. (1997): Vegetationsaufnahmen Mecklenburg-Vorpommern

Bettinger, A. (2012): Zonale Waldgesellschaften in den Kernzonen des Biosphärenreservat Bliesgau

Birger, A. (2009): Vegetationsaufnahmen an der Elbe bei Klöden

Brandes, D. et al. (2007): Verbreitung, Ökologie und Soziologie von Ambrosia artemisiifolia L. in Mitteleuropa

Brinkmann, Ch. (2002): Landschaftsökologische Untersuchungen des NSG "Lanken" (Kreis Ost-Vorpommern)

Buhl, B. (2000): Vegetationskundliche Untersuchungen im Staatsforst Abtshagen (NSG "Wittenhagen")

Conradi, T. et al. (2013): Plant communities and environmental gradients in mires of the Ammergauer Alps (Bavaria, Germany)

Dengler, J. (2001): Die krautige Xerothermvegetation Nordostdeutschlands

Dengler, J. et al. (2006): Die grundwasserfernen Saumgesellschaften Nordostniedersachsens im europäischen Kontext - Teil I: Säume magerer Standorte (Trifolio-Geranietea sanguinei)

Dengler, J. et al. (2007): Die grundwasserfernen Saumgesellschaften Nordost-Niedersachsens im europäischen Kontext - Teil II: Säume nährstoffreicher Standorte (Artemisietea vulgaris) und vergleichende Betrachtung der Saumgesellschaften insgesamt

Diekmann, M. et al. (2012): Das Sumpf-Greiskraut (Senecio paludosus) in Deutschland - Ökologie und Vergesellschaftung

Dittmann, V. (2011): Vegetationsaufnahmen an der Elbe bei Coswig

Doing (1969)

Dörpinghaus, A. (2006)

Dörsing, M. (2004): Vegetationsökologische Untersuchungen und Biotoptypenkartierung des NSG "Haseniederung" (Kreis Steinfurt) zur Veränderung des Grünlandes und daraus resultierende Massnahmen des Naturschutzes

Ewald, J. (2008): Biomasse-Projekt im Nationalpark Bayerischer Wald (Proj. 712)

Ewald, J. et al. (2013): Kiefer am Scheideweg: Heidewälder in der nördlichen Münchener Ebene

Ewald, J. et al. (2016): Nationalpark Hainich in Thüringen

Fischer, A. et al. (2012): Dauerbeobachtungstransekte auf Windwurfflächen im Nationalpark Bayerischer Wald (Proj. 55/649/650)

Fleischer, K. et al. (2010): Zur Ökologie der Wiesen-Glockenblume (Campanula patula) und des Echten Tausendgüldenkrauts (Centaurium erythraea) im Magergrünland Nordwestdeutschlands

Fuchs, R. (2005): Erlen- und Birkenbruchwald-Gesellschaften im Ruhrgebiet

Gehlken, B. (2005): Zur synsystematischen Stellung von Eupatorium cannabinum-Gesellschaften

Gehlken, B. (2010): Beitrag zur Abgrenzung und Untergliederung des Filagini-Vulpietum myuros Oberd. 1938

Glunk, V. et al. (2010): Zur Vegetation von Kleinstrukturen im Mittleren Schwarzwald und deren Eignung als Refugium für Arten der Magerweiden

Gregor, T. et al. (2013): Epilobium brachycarpum: a fast-spreading neophyte in Germany

Hacker, F. (2002): Schlucht- und Hangwälder im Naturpark Mecklenburgische Schweiz und Kummerower See, im Nebeltal und auf Südostrügen

Haeberlein, L. (2017): Veränderungen in der Offenlandvegetation der Greifswalder Oie zwischen 1999 und 2016

Hahn, D. (2001): Die Dünen und Dünentäler im Einflussbereich der Grundwasserbewirtschaftung auf Norderney

Hallmann, S. (2002): Untersuchungen zur Auswirkung von Wildverbiss auf Flora und Vegetation des Luerwaldes bei Arnsberg Hochsauerlandkreis/Märkischer Kreis)

Hammes, V. et al. (2012): Untersuchungen zur längerfristigen Etablierung von Corynephorus canescens-Populationen in einem großflächigen Restitutionsgebiet einer Auenlandschaft Nordwestdeutschlands

Hauswirth, L. (2006): Jahresbericht über die Kontrolle der Bestände des Kriechenden Scheiberichs (Apium repens) im Jahre 2006 - Kreis Soest

Heemann, S. (2003): Vegetationskundliche Untersuchung der Artenvielfalt und Vegetationsstruktur in Sanddorngebüschen im Vergleich zu artenreichen Trockenrasen auf den ostfriesischen Inseln Norderney und Spiekeroog.

Heerde, A. et al. (2006): Verbreitung, Soziologie und Ökologie von Carex buekii Wimm. in Sachsen

Hetzel, I. et al. (2006): Bodensaure Buchenwälder im Übergang vom Bergischen Land zum Niederrheinischen Tiefland

Hinz, T. et al. (2006): Trophiekennzeichnung im anhydromorphen Grünland durch Nährstoffgehalte der oberirdischen Biomasse (Hinz) bzw. Vegetations- und Bodenuntersuchungen auf anhydromorphen Grünlandstandorten als Basis für die Weiterentwicklung des Wasserstufenkonz. (Goldberg)

Huwer, A. et al. (2012): Changes in the species composition of hedgerows

Isermann, M. (2004): Wacholder auf den Ostfriesischen Inseln.

Isermann, M. (2014): Vegetationsökologie der Dünendeiche auf Langeoog und Spiekeroog

Janiesch, P. (2003): Biomasseproduktion, Wasser- und Mineralstoffhaushalt der Empetrum-Heiden auf Norderney

Jörns, S. (2003): Vergleichende vegetationsökologische Untersuchungen von Ackerbrachen im Müritzgebiet

Jost, K. et al. (2004): Vergleichende Untersuchungen an Wald- und Offenlandsöllen im Bereich der Rosenthaler Staffel / nördliche Uckermark

Kaiser, V. (2018): Veränderungen der Vegetation der Karrendorfer Wiesen zwischen 2003 und 2016

Keller, L. (2002): Der Einfluß einer kurzfristigen Beweidung auf die Vegetation des Heideweiergebietes im NSG "Heiliges Meer" bei Hopsten

Kießlich, M. (2001): Vegetationskundliche Untersuchungen von Zweizahngesellschaften (Bidentetea tripartiti) in Mecklenburg-Vorpommern

Kindermann, R. (2016): Vegetationskundliche Erfassung und naturschutzfachliche Bewertung der Wiesen um Hinterhermsdorf (Sächsische Schweiz)

Klauck, E.-J. (2007): Geranium phaeum L. in Saumgesellschaften und Versaumung

Kleikamp, M. (1994): unveröffentlichte Vegetationsaufnahmen

Klos, D. et al. (2014): GlücksSpirale-Projekt Nr. 236/13: Erfassung, Sicherung und Renaturierung von naturnahen Quellstandorten, Schlussbericht. Kreisgruppe Memmingen-Unterallgäu Bund Naturschutz in Bayern e.V., 51 S. + Anhang, Stand 25.02.2014

Klußmann, M. (2004): Vegetationsökologische Untersuchungen zur Re-Etablierung von Salzgrasland am Beispiel der Karrendorfer Wiesen bei Greifswald (Ostsee)

Koska, I. et al. (2005): ALNUS-Projekt, AG 2 Standort, Indikation, GIS: Untersuchungen in Erlenwäldern in MV

Krämer, H. et al. (2007): Syntaxonomie und Ökologie der flussbegleitenden Pioniergesellschaften des Chaenopodion glauci (Klasse: Bidentetea tripartitae) an der unteren Oder

Krumbiegel, A. (2006): Bolboschoenus laticarpus-Röhrichte an der Mittelelbe, eine bisher verkannte Gesellschaft

Kuhbier, H. et al. (2003): Senecio inaequidens DC. als Bestandteil der natürlichen Dünenvegetation auf den Ostfriesischen Inseln.

Landesarbeitsgemeinschaft Vegetationskunde MV, (2000): Arbeitstreffen Elbtal

LANUV NRW (2006): LINFOS-Daten Landesamt für Natur, Umwelt und Verbraucherschutz NRW

Lemke, H. et al. (2000): Zwei Stipa-Arten im Uecker-Randow-Gebiet (M-V) wieder entdeckt - Mit Anmerkungen zum Stipa pennata-Aggregat in Nordostdeutschland

Lemke, T. et al. (2004): Die Vegetation salzbeeinflusster Überflutungsräume auf dem Gebiet der Hansestadt Rostock

Leyer, I. et al. (2007): Multivariate Statistik in der Ökologie

Lienenbecker, H. (2003): Ein Vorkommen des Hundszahngrases (Cynodon dactylon (L.) Pers.) auf Spiekeroog/Ostfriesland.

Linke, C. (2000)

Litza, K. et al. (2019): Hedgerow age affects the species richness of herbaceous forest plants

Litza, K. et al. (2020): The effect of hedgerow density on habitat quality distorts species-area relationships and the analysis of extinction debts in hedgerows

Lohberger, E. (2005): N2000-Kartierung im Nationalpark Bayerischer Wald (Proj. 638)

Metzing, D. et al. (2011): Crassula tillaea (Crassulaceae) auf Baltrum - Erstnachweis für Niedersachsen.

Mickel, R. (2005): Die Verlandungsvegetation des Naturschutzgebietes Galenbecker See / Vorpommern

Müller, A. (1996): Standort und Vegetation auf tiefentwässertem Niedermoor am Beispiel des Gartzer Bruchs

Neumann, A. (2003): Der Einfluss der Wiedervernässung auf Schwarzerlenwälder im Peenetal

Neumann, A. (2005): Die Verbreitung von Deschampsia flexuosa in den Grau- und Braundünen der ostfriesischen Insel Spiekeroog

Partzsch, M. (2009): Populationsstruktur und Vergesellschaftung von Dictamnus albus L. in thermophilen Säumen des unteren Unstruttals (Sachsen-Anhalt)

Peper, J. (2016): Biosphärenreservat Oberlausitzer Heide- und Teichlandschaft

Petersen. J. (2003)

Praktikum Wackerow (2001): Aufnahmen aus dem Vegetationsökologischen Praktikum ab 2001

Rebele, F. (2014): Artenzusammensetzung und Diversität von Calamagrostis epigejos-Dominanzbeständen auf Brachflächen und ehemaligen Rieselfeldern in Berlin

Rüther, C. et al. (2009): Verbreitung, Vergesellschaftung und Ökologie von Lathraea squamaria in Süddeutschland, mit einem Überblick zur Situation in Mitteleuropa

Schmitt, B. et al. (2006): Die Heidenelken-reichen Silikat-Magerrasen der Medebacher Bucht (Südwestfalen/Nordhessen): Ökologie, Syntaxonomie und Management

Schmitt, B. et al. (2010): Vergesellschaftung und Ökologie der Sumpf-Siegwurz (Gladiolus palustris) in Südbayern

Schrautzer, J. et al. (2009): Succession and management of calcareous dry grasslands in the Northern Franconian Jura, Germany

Schröder, C. (2008): Die Heidegebiete Hiddensees -Ein Überblick über die vergangenen 150 Jahre Kulturlandschaftsgeschichte

Schulte, U. (2015): Naturwaldzellen in Nordrhein-Westfalen

Schumann, M. (2004): Standörtliche Charakterisierung mesotropher Birken- und Erlenbruchwälder in der Uckermark

Schwabe-Kratochwil, A. et al. (2001): Vegetationsökologie Borkum.

Schwederski, M.-K. (2012): Die Renaturierung der Vegetation feuchter Dünentäler auf Wangerooge.

Spalke, J. (2008): Biomasse- und Nährstoffallokation von Dünen- und Salzwiesenpflanzen in Bezug zu ihrer Umwelt

Spalke, J. (2008): Biomasse- und Nährstoffallokation von Dünen- und Salzwiesenpflanzen in Bezug zu ihrer Umwelt

Süss, K. et al. (2010): Sukzessionslinien in basenreicher offener Sandvegetation des Binnenlandes: Ergebnisse aus Untersuchungen von Dauerbeobachtungsflächen

Sütering, C. (2003): Landschaftsökologische Untersuchungen in Schwarz-Erlen-Wäldern der Waldlewitz im Rahmen eines Wiedervernässungsprojektes

Tobias, A. (1994): Vegetationsökologische Untersuchungen des Hündfelder Moores unter besonderer Berücksichtigung der bisher erfolgten Optimierungsmaßnahmen

Voigtländer, U. (1994): Die Vegetation des Müritz- Nationalparks: Teil I - Übersichtskartierung der aktuellen Vegetation

Wagner, E. (2014): Naturwaldzellen in Brandenburg

Walentowski, H. et al. (2014): Naturwaldreservatsforschung in Bayern - Auswertung von Vegetationsdaten zur wald-ökologischen Dauerbeobachtung

Wenz, I. et al. (2006): Helio-thermophile Saumgesellschaften auf Xerothermstandorten des Nahe-Gebietes

Wittig, R. et al. (2009): Trittgesellschaften der nordrhein-westfälischen Dörfer

Wolbers, M. (2010): Junco-Caricetum extensae auf Spiekeroog. Standortsfaktoren und Kontaktgesellschaften.

Wulf, F. (2006): Pflanzengesellschaften des Wirtschaftsgrünlands im Altkreis Schmalkalden (Thüringer Wald/Rhön) und ihre Entwicklung zwischen 1960 und 2000

<u>Annex 2:</u> 100 data sets used for the hotspot analysis, of these 48 with point, 8 with line and 37 with polygon data

- Landesamt für Umwelt Brandenburg (2020): survey of habitat types of community interest (EU Habitats Directive 92/43/EEC) and biotopes
  - Excerpt of location data (point, line and polygon).
  - Excerpt of site information.
  - Excerpt of information on species.
  - Excerpt of information on vascular plant species.
- Landesanstalt für Umwelt Baden-Württemberg (2020): Species information system ARTIS
  - Excerpt of site information and location data (point) of Regierungsbezirk Freiburg of the years 1900 to 2020.
  - Excerpt of site information and location data (point) of Regierungsbezirk Karlsruhe of the years 1903 to 2020.
  - Excerpt of site information and location data (point) of Regierungsbezirk Stuttgart of the years 1900 to 2020.
  - Excerpt of site information and location data (point) of Regierungsbezirk Tübingen of the years 1900 to 2020.
- Bayerisches Landesamt für Umwelt (2020): Floristic records from survey studies of the Bayerisches Landesamt für Umwelt. Bayernflora. Point data. DWB export, processed and aggregated data. Records of the years 2000 to 2020. Version: 20190903. Data Publisher: Staatliche Naturwissenschaftliche Sammlungen Bayerns – SNSB IT Center, München. https://wiki.bayernflora.de/web/Floristic_records_from_survey_studies_of_the_Bayerisches_Landesamt_f%C3%BCr_Umwelt
- Bayerisches Landesamt für Umwelt (2020): survey of habitat types of community interest (EU Habitats Directive 92/43/EEC). www.lfu.bayern.de.
  - Excerpt of location data (polygon) of the years 1980 to 2019.
  - Excerpt of site information of the years 1920 to 2020.
- Bayerisches Landesamt für Umwelt (2020): survey of biotopes of the Bavarian lowlands. Geodata services, state 29.01.2020, www.lfu.bayern.de.
  - Excerpt of location data (polygon) of the years 1980 to 2018.
  - Excerpt of site information of the years 1975 to 2016.
- Bayerisches Landesamt für Umwelt (2020): Survey of biotopes of the Bavarian Alps. Geodata services, state 29.01.2020, www.lfu.bayern.de.
  - Excerpt of location data (polygon) of the years 1990 to 2018.
  - Excerpt of site information of the years 1908 to 2018.
- Bayerisches Landesamt für Umwelt (2020): Survey of biotopes of the Bavarian urban areas. Geodata services, state 29.01.2020, www.lfu.bayern.de.
  - Excerpt of location data (polygon) of the years 1979 to 2019.
  - Excerpt of site information of the years 1907 to 2018.
- Kraus D, Schuck A, Bebi P, Blaschke M, Bütler R, Flade M, Heintz W, Krumm F, Lachat T, Larrieu L, Lehnerova L, Levin M, Mergner U, Pach M, Paillet Y, Pyttel P, Rydkvist T, Santopuoli G, Sever K, Sturm K, Vandekerkhove K, Winter S, Witz M (2017). Spatially explicit database of tree related microhabitats (TreMs). Version 1.2. Integrate+ project. Version 1.6. Institut national de recherche pour l'agriculture, l'alimentation et l'environnement (INRAE). Excerpt of site information and location data (point) of the years 2014 to 2017. https://doi.org/10.15468/dl.ym95pw accessed via GBIF.org on 22.09.2020.

- de Vries H, Lemmens M. Observation.org, Nature data from around the World. Observation.org. Excerpt of site information and location data (point) of the years 2000 to 2020. https://doi.org/10.15468/dl.4v6rgd accessed via GBIF.org on 23.09.2020.
- Ueda K (2020). iNaturalist Research-grade Observations. iNaturalist.org. Excerpt of site information and location data (point) of the years 2003 to 2020. https://doi.org/10.15468/dl.83h4hx accessed via GBIF.org on 23.09.2020.
- Naturgucker.de (2020): Occurrence dataset of the years 2000 to 2020 (point). https://doi.org/10.15468/dl.pwva95 accessed via GBIF.org on 23.09.2020.
- Vegetweb.de (2020): species observations (point) of several sources as listed in annex 1
- Vegetweb.de (2020): species observations (polygon) of several sources as listed in annex 1
- Julius Kühn-Institut (2020): observations of wild celery. Excerpt of site information on wild celery species and location data (point) of the years 2012 to 2016 as part of the project "Genetic reserves for wild celery species (Apium and Helosciadium) as part of a German Network of genetic Reserves".
- Freie Hansestadt Bremen (2020): Excerpt of site information on the target species of Bremen and location data (point) of the years 2009 to 2019.
- Hessisches Landesamt für Naturschutz, Umwelt und Geologie (2020): survey of basis information on Special Areas of Conservation and data produced via a work contract. Excerpt of occurrence data (point) of Festuca ovina. Wiesbaden
- Hessisches Landesamt für Naturschutz, Umwelt und Geologie (2020): survey of basis information on Special Areas of Conservation, survey of biotopes, additional assessment and data produced via work contracts
    - Excerpt of occurrence data (point) on CWR. Part 1. Wiesbaden
    - Excerpt of occurrence data (point) on CWR. Part 2. Wiesbaden
    - Excerpt of occurrence data (point) on CWR. Part 3. Wiesbaden
- Landesamt für Umwelt, Naturschutz und Geologie Mecklenburg-Vorpommern (2020): Survey of biotopes
    - Excerpt of location data (polygon) of the years 1996 to 2013.
    - Excerpt of site information of the years 1991 to 2012.
- Niedersächsischer Landesbetrieb für Wasserwirtschaft, Küsten- und Naturschutz (2021): survey programme on plant species, state 11.11.2021
    - Excerpt of occurrence data on vascular plants, which are not threatened (not applied to the local Red List categories "0", "1", "2"), from the vascular plant database of the years 2010 to 2020.
    - Excerpt of location data (polygon) on vascular plants, which are not threatened (not applied to the local Red List categories "0", "1", "2"), of surveyed regions of the years 2010 to 2020.
- Landesamt für Natur, Umwelt und Verbraucherschutz Nordrhein-Westfalen (2020): collection of landscape information NRW (LINFOS): plant sites
    - Excerpt of site information on CWR and location data (polygon) of the years 2000 to 2019.
    - Excerpt of site information on CWR and location data (line) of the years 2000 to 2019.
    - Excerpt of site information on CWR and location data (point) of the years 2000 to 2019.
    - Excerpt of occurrence information on CWR of the years 2000 to 2019.
- Landesamt für Natur, Umwelt und Verbraucherschutz Nordrhein-Westfalen (2020): collection of landscape information NRW (LINFOS): vegetation surveys

    - Excerpt of information on priority CWR and location data (polygon) of the years 2003 to 2019.
    - Excerpt of information on priority CWR and location data (point) of the years 2000 to 2019.
    - Excerpt of information on non-priority CWR and location data (polygon) of the years 2003 to 2020.
    - Excerpt of information on non-priority CWR and location data (point) of the years 2001 to 2020.
- Landesamt für Natur, Umwelt und Verbraucherschutz Nordrhein-Westfalen (2020): collection of landscape information NRW (LINFOS): biotopes
    - Excerpt of information on priority CWR and location data (polygon) of the years 2000 to 2020.
    - Excerpt of information on priority CWR and location data (line) of the years 2000 to 2019.
    - Excerpt of information on priority CWR and location data (point) of the years 2001 to 2019.
    - Excerpt of information on non-priority CWR and location data (polygon) of the years 2000 to 2020. Part 1.
    - Excerpt of information on non-priority CWR and location data (polygon) of the years 2000 to 2020. Part 2.
    - Excerpt of information on non-priority CWR and location data (line) of the years 2000 to 2020.
    - Excerpt of information on non-priority CWR and location data (point) of the years 2000 to 2017.
- Landesamt für Umwelt Rheinland-Pfalz (2020): data information system on species
    - Excerpt of information on flowering plants and location data (polygon) of the years 2008 to 2012.
    - Excerpt of information on flowering plants and location data (line) until 2003.
    - Excerpt of information on flowering plants and location data (point) of the years 1993 to 2014.
- Landesamt für Umwelt Rheinland-Pfalz (2020): LANIS – geo data system of the nature conservation authority of Rhineland-Palatinate
    - Excerpt of information on biotopes and plants and location data (point, line and polygon) of the years 1968 to 2019.
    - Excerpt information on biotopes and location data (polygon).
    - Excerpt of information on habitat types of community interest (EU Habitats Directive 92/43/EEC) and location data (polygon).
- Sächsisches Landesamt für Umwelt, Landwirtschaft und Geologie (2020): survey of habitat types of community interest (EU Habitats Directive 92/43/EEC)
    - Site information on habitat types of community interest (EU Habitats Directive 92/43/EEC) typical for priority CWR of the years 2010 to 2020.
    - Location data (polygon) of the years 2002 to 2020. WFS service.
    - Location data (line). WFS service.
    - Location data (point) of the years 2004 to 2019. WFS service.
- Groom Q (2019). Vascular plants of Amrum 2007-2008. Version 7.2. Botanic Garden Meise. Occurrence dataset (points) https://doi.org/10.15468/kt1c2i accessed via GBIF.org on 06.10.2020.

- Landesamt für Landwirtschaft, Umwelt und ländliche Räume Schleswig-Holstein (2020): Survey of biotopes
    - Site information and location data (point) of the years 2014 to 2015.
    - Site information and location data (point) of the years 2016 to 2018.
- Landesamt für Landwirtschaft, Umwelt und ländliche Räume Schleswig-Holstein (2020): flora.sh (Stand 12.05.2020)
    - Site information and location data (point) of the years 2014 to 2020.
    - Site information and location data (point) of the years 2016 to 2020.
    - Site information and location data (point) of the years 2009 to 2020.
    - Site information and location data (point) of the year 2018.
- Ministerium für Umwelt und Verbraucherschutz Saarland - Zentrum für Biodokumentation (2020): Floristic survey
    - Excerpt of Site information on Allium scorodoprasum and location data (point) of the year 2001.
    - Excerpt of site information on Allium ursinum and location data (point) of the years 2005 to 2012.
    - Excerpt of site information on Arnica montana and location data (point) of the years 2004 to 2012.
    - Excerpt of site information on Carex brizoides and location data (point) of the year 2002.
    - Excerpt site information on Festuca pallens and location data (point) of the year 2005.
    - Excerpt of site information on Fragaria viridis and location data (point) of the years 2003 to 2010.
    - Excerpt of site information on Hordeum secalinum and location data (point) of the year 2013.
    - Excerpt of site information on Lactuca virosa and location data (point) of the year 2008.
    - Excerpt of site information on Medicago falcata and location data (point) of the year 2003.
    - Excerpt of site information on Poa chaixii and location data (point) of the years 2002 to 2004.
    - Excerpt of site information on Ribes alpinum and location data (point) of the years 2004 to 2012.
    - Excerpt of site information on Valerianella dentata and location data (point) of the years 2010 to 2012.
    - Excerpt of site information on Valerianella rimosa and location data (point) of the years 2010 to 2012.
    - Excerpt of site information on Vicia lathyroides and location data (point) of the year 2002.
    - Excerpt of site information on Vicia tenufolia and location data (point) of the years 2003 to 2006.
    - Excerpt of site information on Achillea ptarmica and location data (polygon) of the years 2012 to 2020.
    - Excerpt of site information on Arum maculatum and location data (polygon) of the years 2012 to 2020.
    - Excerpt of site information on Asparagus officinalis and location data (polygon) of the years 2012 to 2015.

    - Excerpt of site information on Carum carvi and location data (polygon) of the years 2012 to 2020.
    - Excerpt of site information on Festuca brevipila and location data (polygon) of the years 2012 to 2018.
    - Excerpt of site information on Festuca filiformis and location data (polygon) of the years 2012 to 2020.
    - Excerpt of site information on Festuca guestfalica and location data (polygon) of the years 2012 to 2018.
    - Excerpt of site information on Festuca pratensis and location data (polygon) of the years 2012 to 2020.
    - Excerpt of site information on Hypericum hirsutum and location data (polygon) of the years 2012 to 2020.
    - Excerpt of site information on Hypericum humifusum and location data (polygon) of the years 2012 to 2020.
    - Excerpt of site information on Hypericum perforatum and location data (polygon) of the years 2012 to 2020.
    - Excerpt of site information on Prunus maheleb and location data (polygon) of the years 2016.
    - Excerpt of site information on Pyrus pyraster and location data (polygon) of the years 2012 to 2020.
    - Excerpt of site information on Thymus praecox and location data (polygon) of the years 2012 to 2016.
    - Excerpt of site information on Trifolium campestre and location data (polygon) of the years 2012 to 2020.
- Landesamt für Umweltschutz Sachsen-Anhalt (2020): habitat types of community interest (EU Habitats Directive 92/43/EEC) in Saxony-Anhalt, including biotopes and use. Excerpt of site information and location data (point, line and polygon).
- Thüringer Landesamt für Umwelt, Bergbau und Naturschutz (2020): survey of habitat types of community interest (EU Habitats Directive 92/43/EEC)
    - Excerpt of location data (polygon) on habitat types of community interest (EU Habitats Directive 92/43/EEC) typical for priority CWR of the districts Greiz, Hildburghausen, Ilm-Kreis and Nordhausen of the years 2010 to 2020.
    - Excerpt of site information on habitat types of community interest (EU Habitats Directive 92/43/EEC) typical for priority CWR of the districts Greiz, Hildburghausen, Ilm-Kreis and Nordhausen of the years 2010 to 2020.
- Thüringer Landesamt für Umwelt, Bergbau und Naturschutz (2020): Survey of biotopes
    - Excerpt of location data (polygon) on biotope types typical for priority CWR of the districts Greiz, Hildburghausen, Ilm-Kreis and Nordhausen of the years 1998 to 2020.
    - Excerpt of site information on biotope types typical for priority CWR of the districts Greiz, Hildburghausen, Ilm-Kreis and Nordhausen of the years 1998 to 2020.

<u>Annex 3:</u> Methods: details

The processing and filtering of the raw geodata with ArcGIS Pro v. 2.8.3 (ESRI Inc.):

The datasets were imported into a FileGeodatabase and the spatial reference was standardised by transformation into the coordinate reference system ETRS 89 (EPSG code: 25832). Data were removed that i) were recorded before 1st January 2000, ii) had no correctly assigned spatial reference, iii) were located outside Germany (ArcGIS function "intersect" and shape of Germany's administrative boundaries (© GeoBasis-DE /BKG 2016)) or iv) were not sufficiently precise for the planned analyses (polygon area or line length more than 8,099 km² or 4,027.99 m respectively, which are the average size and diagonal length of the hereafter used grid cells). v) If CS observations: They are often located in residential areas and along infrastructure routes, which are unsuitable as GR. Therefore, CS data from observations with a soil sealing of more than 30 % (data set *Bodenversiegelung* as 100 x 100 m grid of the IÖR-Monitor © Leibniz Institute of Ecological Urban and Regional Development 2021) were removed. vi) CS data indicating uncertainties in observations (e.g. spatial reference or species identification) were also excluded.

Point data often had no information on location accuracy. Here, it was assumed that they were close to the site, as the data were regularly located in suitable areas and not clustered at e.g. specific institutions or mid-points of administrative units. Some data sets required linking the area geometries with the associated species lists via key fields ("Add Relate" function).

<u>Annex 4:</u> Number of priority CWR typical for VT7, VT16 and VT 18 in a grid cell based on the collected occurrence data (federal state borders © GeoBasis-DE /BKG 2016)

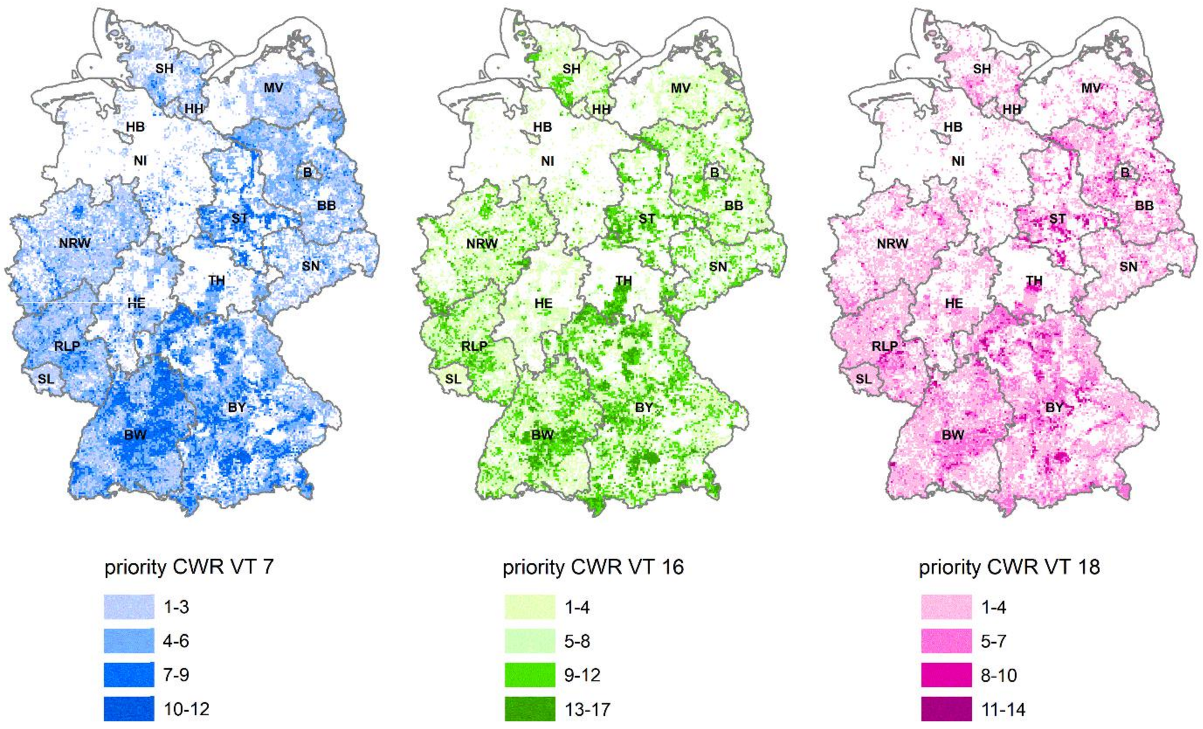

<u>Annex 5:</u> 1,000 hotspots cells for the potential pool of GR distributed across the 7 terrestrial BGR of Germany

253 hotspots of priority CWR in total, 247 hotspots of priority CWR typical for VT7, 247 hotspots of priority CWR typical for VT16 and 253 hotspots of priority CWR typical for VT18. 414 hotspot cells are in the same location and overlap each other.

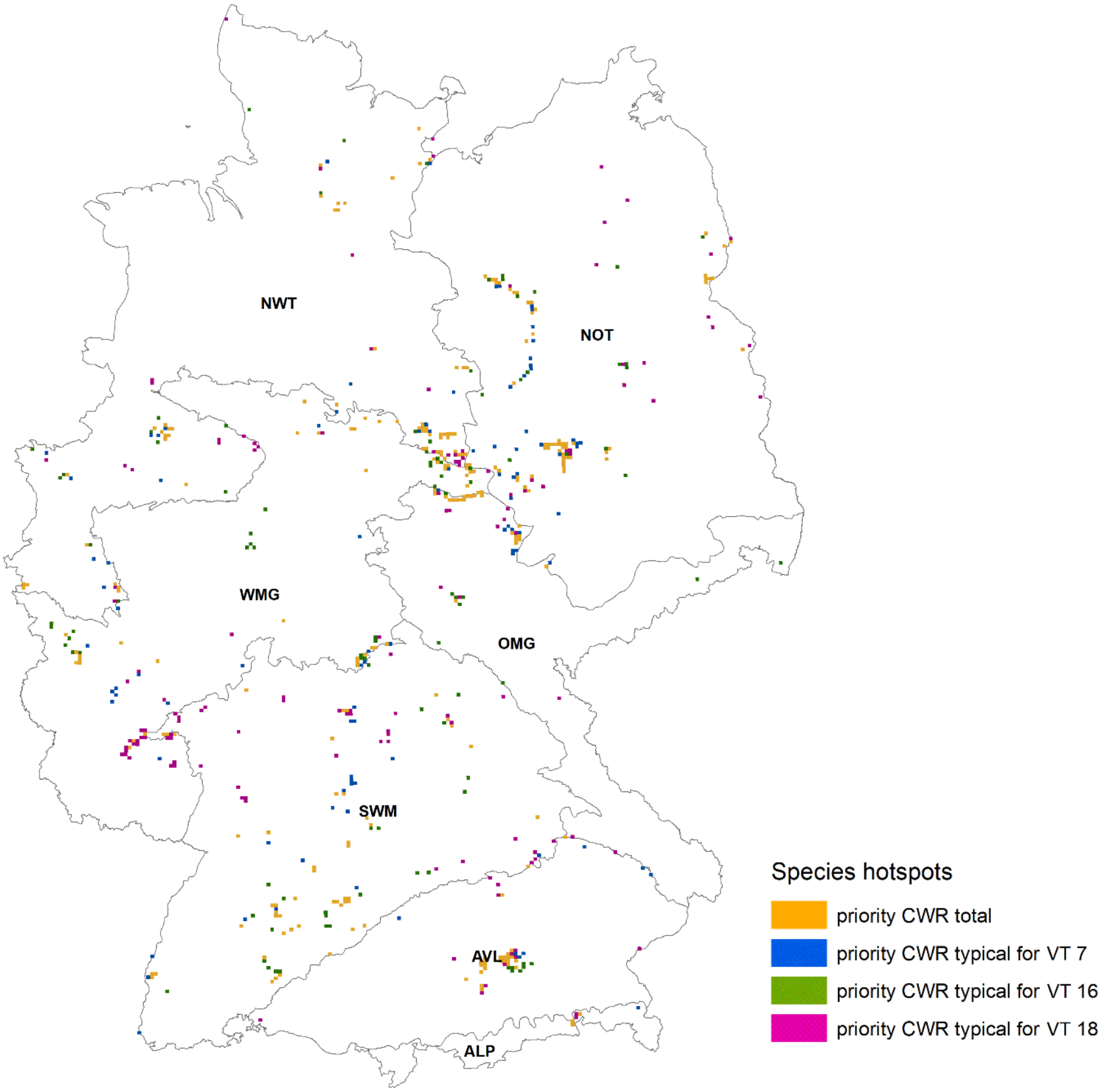

Supplement: Supplementary file 2 — Supplementary Material 2 [file 40529_2025_473_MOESM2_ESM.pdf]
