# Supplementary material for: Identification of hotspots of crop wild relatives in Germany to promote their in situ conservation in a network of genetic reserves
Source: Bot Stud. 2025 Sep 2;66:27. doi: 10.1186/s40529-025-00473-z (PMC12405085; doi:10.1186/s40529-025-00473-z)
Supplement: Supplementary file 3 — Supplementary Material 3 [file 40529_2025_473_MOESM3_ESM.pdf]

```sql
-- script PostgreSQL: calculation of CWR and priority CWR per grid cell

--A01: union all point data sets, harmonize the columns

create table hot_pt."01_tab_union_boden_less30"
AS
	select id, geom, species, cast (null as bigint) as taxnr, 'gbif_ampt' as quelle, cast (gbifid as varchar) as daten_id ,scientific as daten_art, cast (null as float8) hemerobie_, cast (null as float8) bodenversi
	from hot_pt.gbif_ampt
union all
	select id, geom, species, taxnr, quelle, daten_id, daten_art, hemerobie_, bodenversi
	from hot_pt.land_inaturalist_less30
union all
	select id, geom, species, taxnr, quelle, daten_id, daten_art, hemerobie_, bodenversi
	from hot_pt.gbif_naturgucker_less30
union all
	select id, geom, species, taxnr, quelle, daten_id, daten_art, hemerobie_, bodenversi
	from hot_pt.land_observation_less30
union all
	select id, geom, species, cast (null as bigint) taxnr, 'gbif_trept' as quelle, cast (gbifid as varchar) as daten_id,scientific as daten_art, cast (null as float8) hemerobie_, cast (null as float8) bodenversi
	from hot_pt.gbif_trept
union all
	select id, geom, spezies as species, cast (null as bigint) taxnr, 'gbif_gespt' as quelle, pop_id as daten_id , null as daten_art, cast (null as float8) hemerobie_, cast (null as float8) bodenversi
	from hot_pt.gbif_gespt
union all
	select id, geom, name as species, cast (null as bigint) taxnr, 'd_vegpt' as quelle, null as daten_id , null as daten_art, cast (null as float8) hemerobie_, cast (null as float8) bodenversi
	from hot_pt.d_vegpt
union all
	select id, geom, taxname as species, taxnr as taxnr, 'by_byflpt' as quelle, null as daten_id , null as daten_art, cast (null as float8) hemerobie_, cast (null as float8) bodenversi
	from hot_pt.by_byflpt
union all
	select id, geom, pfl_wis as species, cast (null as bigint) taxnr, 'nrw_btpriopt' as quelle, kennung as daten_id , null as daten_art, cast (null as float8) hemerobie_, cast (null as float8) bodenversi
	from hot_pt.nrw_btpriopt
union all
	select id, geom, wea_name as species, cast (null as bigint) taxnr, 'nrw_vapriopt' as quelle, kennung as daten_id , pfl_wis as daten_art, cast (null as float8) hemerobie_, cast (null as float8) bodenversi
	from hot_pt.nrw_vapriopt
union all
	select id, geom, wel_name as species, cast (null as bigint) taxnr, 'nrw_vawelpt' as quelle, kennung as daten_id , pfl_wis as daten_art, cast (null as float8) hemerobie_, cast (null as float8) bodenversi
	from hot_pt.nrw_vawelpt
union all
	select id, geom, art_wiss as species, cast (null as bigint) taxnr, 'rlp_pt' as quelle, null as daten_id , null as daten_art, cast (null as float8) hemerobie_, cast (null as float8) bodenversi
	from hot_pt.rlp_pt
union all
	select id, geom, akzart as species, bfntax as taxnr, 'sh_fl01pt' as quelle, cast (beoid as varchar) as daten_id , artnam as daten_art, cast (null as float8) hemerobie_, cast (null as float8) bodenversi
	from hot_pt.sh_fl01pt
union all
	select id, geom, akzart as species, bfntax as taxnr, 'sh_fl08pt' as quelle, cast (beoid as varchar) as daten_id , artnam as daten_art, cast (null as float8) hemerobie_, cast (null as float8) bodenversi
	from hot_pt.sh_fl08pt
union all
```

```
        select id, geom, akzart as species, bfntax as taxnr, 'sh_fl09pt' as quelle, cast (beoid as varchar) as daten_id , artnam as daten_art, cast (null as float8) hemerobie_, cast (null as float8) bodenversi
        from hot_pt.sh_fl09pt
union all
        select id, geom, akzart as species, bfntax as taxnr, 'sh_flgespt' as quelle, cast (beoid as varchar) as daten_id , artnam as daten_art, cast (null as float8) hemerobie_, cast (null as float8) bodenversi
        from hot_pt.sh_flgespt
union all
        select id, geom, art as species, cast (null as bigint) taxnr, 'sh_bkpt' as quelle, bez as daten_id , null as daten_art, cast (null as float8) hemerobie_, cast (null as float8) bodenversi
        from hot_pt.sh_bkpt
union all
        select id, geom, sippe as species, cast (null as bigint) taxnr, 'sl_allscopt' as quelle,  cast (fid_abds_2 as varchar) as daten_id , null as daten_art, cast (null as float8) hemerobie_, cast (null as float8) bodenversi
        from hot_pt.sl_allscopt
union all
        select id, geom, sippe as species, cast (null as bigint) taxnr, 'sl_allurspt' as quelle,  cast (fid_abds_2 as varchar) as daten_id , null as daten_art, cast (null as float8) hemerobie_, cast (null as float8) bodenversi
        from hot_pt.sl_allurspt
union all
        select id, geom, sippe as species, cast (null as bigint) taxnr, 'sl_arnmonpt' as quelle,  cast (fid_abds_2 as varchar) as daten_id , null as daten_art, cast (null as float8) hemerobie_, cast (null as float8) bodenversi
        from hot_pt.sl_arnmonpt
union all
        select id, geom, sippe as species, cast (null as bigint) taxnr, 'sl_carbript' as quelle,  cast (fid_abds_2 as varchar) as daten_id , null as daten_art, cast (null as float8) hemerobie_, cast (null as float8) bodenversi
        from hot_pt.sl_carbript
union all
        select id, geom, sippe as species, cast (null as bigint) taxnr, 'sl_fespalpt' as quelle,  cast (fid_abds_2 as varchar) as daten_id , null as daten_art, cast (null as float8) hemerobie_, cast (null as float8) bodenversi
        from hot_pt.sl_fespalpt
union all
        select id, geom, sippe as species, cast (null as bigint) taxnr, 'sl_fravirpt' as quelle,  cast (fid_abds_2 as varchar) as daten_id , null as daten_art, cast (null as float8) hemerobie_, cast (null as float8) bodenversi
        from hot_pt.sl_fravirpt
union all
        select id, geom, sippe as species, cast (null as bigint) taxnr, 'sl_horsecpt' as quelle,  cast (fid_abds_2 as varchar) as daten_id , null as daten_art, cast (null as float8) hemerobie_, cast (null as float8) bodenversi
        from hot_pt.sl_horsecpt
union all
        select id, geom, sippe as species, cast (null as bigint) taxnr, 'sl_lacvirpt' as quelle,  cast (fid_abds_2 as varchar) as daten_id , null as daten_art, cast (null as float8) hemerobie_, cast (null as float8) bodenversi
        from hot_pt.sl_lacvirpt
union all
        select id, geom, sippe as species, cast (null as bigint) taxnr, 'sl_medfalpt' as quelle,  cast (fid_abds_2 as varchar) as daten_id , null as daten_art, cast (null as float8) hemerobie_, cast (null as float8) bodenversi
        from hot_pt.sl_medfalpt
union all
        select id, geom, sippe as species, cast (null as bigint) taxnr, 'sl_poachapt' as quelle,  cast (fid_abds_2 as varchar) as daten_id , null as daten_art, cast (null as float8) hemerobie_, cast (null as float8) bodenversi
        from hot_pt.sl_poachapt
union all
        select id, geom, sippe as species, cast (null as bigint) taxnr, 'sl_ribalppt' as quelle,  cast (fid_abds_2 as varchar) as daten_id , null as daten_art, cast (null as float8) hemerobie_, cast (null as float8) bodenversi
        from hot_pt.sl_ribalppt
union all
        select id, geom, sippe as species, cast (null as bigint) taxnr, 'sl_valdenpt' as quelle,  cast (fid_abds_2 as varchar) as daten_id , null as daten_art, cast (null as float8) hemerobie_, cast (null as float8) bodenversi
```

```sql
        from hot_pt.sl_valdenpt
union all
        select id, geom, sippe as species, cast (null as bigint) taxnr, 'sl_valrimpt' as quelle,  cast (fid_abds_2 as varchar) as daten_id , null as daten_art, cast (null as float8) hemerobie_, cast (null as float8) bodenversi
        from hot_pt.sl_valrimpt
union all
        select id, geom, sippe as species, cast (null as bigint) taxnr, 'sl_viclatpt' as quelle,  cast (fid_abds_2 as varchar) as daten_id , null as daten_art, cast (null as float8) hemerobie_, cast (null as float8) bodenversi
        from hot_pt.sl_viclatpt
union all
        select id, geom, sippe as species, cast (null as bigint) taxnr, 'sl_victenpt' as quelle,  cast (fid_abds_2 as varchar) as daten_id , null as daten_art, cast (null as float8) hemerobie_, cast (null as float8) bodenversi
        from hot_pt.sl_victenpt
union all
        select id, geom, gueltiger_ as species, cast (null as bigint) taxnr, 'bw_artpt' as quelle,  null as daten_id , null as daten_art, cast (null as float8) hemerobie_, cast (null as float8) bodenversi
        from hot_pt.bw_artpt
union all
        select id, geom, gueltiger_ as species, cast (null as bigint) taxnr, 'bw_kapt' as quelle,  null as daten_id , null as daten_art, cast (null as float8) hemerobie_, cast (null as float8) bodenversi
        from hot_pt.bw_kapt
union all
        select id, geom, gueltiger_  as species, cast (null as bigint) taxnr, 'bw_spt' as quelle,  null as daten_id , null as daten_art, cast (null as float8) hemerobie_, cast (null as float8) bodenversi
        from hot_pt.bw_spt
union all
        select id, geom, gueltiger_ as species, cast (null as bigint) taxnr, 'bw_tuept' as quelle,  null as daten_id , null as daten_art, cast (null as float8) hemerobie_, cast (null as float8) bodenversi
        from hot_pt.bw_tuept
union all
        select id, geom, lat_name as species, cast (null as bigint) taxnr, 'hb_flpt' as quelle,  null as daten_id , vollname as daten_art, cast (null as float8) hemerobie_, cast (null as float8) bodenversi
        from hot_pt.hb_flpt
union all
        select distinct a.id, a.geom, b.art as species, cast (b.taxnr as bigint) as taxnr, 'st_pt' as quelle, null as daten_id ,null as daten_art, cast (null as float8) hemerobie_, cast (null as float8) bodenversi
        from hot_pt.st_pt a
        left join hot_pt.st_arten b on a.lrt_guid = b.ref_guid
        where b.art is not null
union all
        select distinct a.id, a.geom, c.art_wissenschaftl as species, cast (null as bigint) as taxnr, 'sa_pt' as quelle, cast (a.lff_id as varchar) as daten_id , null as daten_art , cast (null as float8) hemerobie_, cast (null as float8) bodenversi
        from hot_pt.sa_pt a
        left join hot_pt.sa_vak_arten b on cast (a.lff_id as varchar) = b.lff_id
        left join hot_pt.sa_arten c on b.vak_id = c.vak_id
        where c.art_wissenschaftl is not null
union all
        select distinct a.id, a.geom, b.pflanzenart as species, cast (null as bigint) as taxnr, 'rlp_lanispt' as quelle, a.kennung as daten_id ,null as daten_art , cast (null as float8) hemerobie_, cast (null as float8) bodenversi
        from hot_pt.rlp_lanispt a
        left join hot_pt.rlp_lanis_arten b on a.kennung = b.kennung
        where b.pflanzenart is not null
union all
        select id, geom, wel_name as species, cast (null as bigint) taxnr, 'nrw_btwelpt' as quelle, kennung as daten_id , null as daten_art, cast (null as float8) hemerobie_, cast (null as float8) bodenversi
        from hot_pt.nrw_btwelpt
union all
        select id, geom, wel_name as species, cast (null as bigint) taxnr, 'nrw_fpwelpt' as quelle, kennung as daten_id , null as daten_art, cast (null as float8) hemerobie_, cast (null as float8) bodenversi
```

```
        from hot_pt.nrw_fpwelpt
union all
        select id, geom, art as species, cast (null as bigint) taxnr, 'sh_bk1415pt' as quelle, bez as daten_id , null as daten_art, cast (null as float8) hemerobie_ , cast (null as float8) bodenversi
        from hot_pt.sh_bk1415pt
union all
        select distinct a.id as id, a.geom, c.name as species, cast (null as bigint) taxnr, 'bb_pt' as quelle, null as daten_id ,null as daten_art, cast (null as float8) hemerobie_, cast (null as float8) bodenversi
        from hot_pt.bb_pt a
        left join hot_pt.bb_dflora b on a.pk_ident = b.fk_ident
        left join hot_pt.bb_bbk_arten c on b.fk_arten = c.pk_artcode
        where c.name is not null
union all
        select id, geom, art_wiss as species, cast (null as bigint) as taxnr, 'he_1pt' as quelle, guid_daten as daten_id , null as daten_art, cast (null as float8) hemerobie_, cast (null as float8) bodenversi
        from hot_pt.he_1pt
union all
        select id, geom, art_wiss as species, cast (null as bigint) as taxnr, 'he_2pt' as quelle, guid_daten as daten_id , null as daten_art, cast (null as float8) hemerobie_, cast (null as float8) bodenversi
        from hot_pt.he_2pt
union all
        select id, geom, art_wiss as species, cast (null as bigint) as taxnr, 'he_fopt' as quelle, guid_daten as daten_id , null as daten_art, cast (null as float8) hemerobie_, cast (null as float8) bodenversi
        from hot_pt.he_fopt
union all
        select id, geom, art_wiss as species, cast (null as bigint) as taxnr, 'he_pt' as quelle, guid_daten as daten_id , null as daten_art, cast (null as float8) hemerobie_, cast (null as float8) bodenversi
        from hot_pt.he_pt;


--A02: separate the taxonomic name in genus and species name

CREATE OR REPLACE VIEW hot_pt."02_01_pos1_boden_less30"
 AS
  select a.*, "position"(a.species, ' ') AS pos1
  FROM hot_pt."01_tab_union_boden_less30" a;

CREATE OR REPLACE VIEW hot_pt."02_02_gat_boden_less30"
 AS
select a.*,
    "substring"(a.species::text, 1, a.pos1 - 1) AS gat,
    "substring"(a.species::text, a.pos1 + 1, 25) AS name_min_gat
  FROM hot_pt."02_01_pos1_boden_less30" a
  where pos1 <> '0';

CREATE OR REPLACE VIEW hot_pt."02_03_pos2_boden_less30"
 AS
 select a.*,
    "position"(a.name_min_gat, ' ') AS pos2
  FROM hot_pt."02_02_gat_boden_less30" a;

CREATE OR REPLACE VIEW hot_pt."02_04_gatart_boden_less30"
 AS
  select a.*,
        CASE
            WHEN a.pos2 = 0 THEN a.name_min_gat
            ELSE "substring"(a.name_min_gat, 1, a.pos2 - 1)
        END AS art
  FROM hot_pt."02_03_pos2_boden_less30" a;

create table hot_pt."02_union_gatart_boden_less30"
AS
select id, geom, species, taxnr, quelle, daten_id, daten_art, hemerobie_, bodenversi, gat, art
from hot_pt."02_04_gatart_boden_less30";

CREATE index "02_union_gatart_boden_less30_gist" ON hot_pt.
```

```
"02_union_gatart_boden_less30" using gist(geom);
CREATE index "02_union_gatart_boden_less30_btree" ON hot_pt.
"02_union_gatart_boden_less30" (gat, art);


--A03+04: join CWR information to the point shape using genus and species names as
joins fields

CREATE OR REPLACE VIEW hot_pt."03_union_taxwel_boden_less30"
 AS
select a.*, b.ak2, b.name as ak2_name, b.wel, b.welprio, b.form1, b.form2, b.form3, b.
form4, b.form5, b.form6, b.form7, b.form8, b.form9, b.form10,
b.form11, b.form12, b.form13, b.form14, b.form15, b.form16, b.form17, b.form18, b.
form19, b.form20,
b.form21, b.form22, b.form23, b.form24
from hot_pt."02_union_gatart_boden_less30" a
left join hot_pt.welliste b on (a.gat||' '||a.art) = (b.gat||' '||b.art)
where b.ak2 is not null;

create table hot_pt."04_tab_union_taxwel_boden_less30"
AS
select *
from hot_pt."03_union_taxwel_boden_less30"
order by ak2_name;

ALTER TABLE hot_pt."04_tab_union_taxwel_boden_less30"
    ALTER COLUMN geom TYPE geometry(MultiPoint,25832) USING ST_Multi(ST_Force2d(geom
    ));

create index "04_tab_union_taxwel_boden_less30_gist" on hot_pt.
"04_tab_union_taxwel_boden_less30" using gist (geom);


--A05: join grid cell shape to point shape

CREATE OR REPLACE VIEW hot_pt."05_union_mtb16_boden_less30"
AS
select a.*, b.id as id_mtb16, b.name as name_mtb16, b.geom as geom_mtb16
from hot_pt."04_tab_union_taxwel_boden_less30" a
left join hot_pt.mtb16_2d b on st_within(a.geom, b.geom)
where b.geom is not null;

create table hot_pt."05_tab_union_mtb16_ohne_boden_less30"
AS
select distinct a.id,
    a.geom,
    a.species,
--  a.taxnr,
    a.quelle,
    a.daten_id,
    a.daten_art,
--    a.hemerobie_,
--    a.bodenversi,
    a.gat,
    a.art,
    a.ak2,
    a.ak2_name,
    a.wel,
    a.welprio,
    a.form1,
    a.form2,
    a.form3,
    a.form4,
    a.form5,
    a.form6,
    a.form7,
    a.form8,
    a.form9,
    a.form10,
    a.form11,
    a.form12,
    a.form13,
    a.form14,
```

```sql
    a.form15,
    a.form16,
    a.form17,
    a.form18,
    a.form19,
    a.form20,
    a.form21,
    a.form22,
    a.form23,
    a.form24,
    a.id_mtb16,
    a.name_mtb16,
    a.geom_mtb16
from hot_pt."05_union_mtb16_boden_less30" a;

ALTER TABLE hot_pt."05_tab_union_mtb16_ohne boden_less30"
    ADD CONSTRAINT "pkey_05_tab_union_mtb16_ohne boden_less30" PRIMARY KEY (id, species, quelle, ak2, ak2_name, id_mtb16, name_mtb16);

create index "05_tab_union_mtb16_ohne boden_less30_btree" on hot_pt."05_tab_union_mtb16_ohne boden_less30"(name_mtb16,id_mtb16,ak2);


--A06: create species list including the assignment between species and grid cells (long version)

CREATE OR REPLACE VIEW hot_pt."06_union_arten_lang_ohne boden_less30"
 AS
select distinct a.*, c.count as anz_wel
from hot_pt."05_tab_union_mtb16_ohne boden_less30" a
right join (select b.name_mtb16, b.id_mtb16, count (distinct b.ak2) from hot_pt."05_tab_union_mtb16_ohne boden_less30" b group by b.name_mtb16, b.id_mtb16) c
on (a.name_mtb16||a.id_mtb16) = (c.name_mtb16||c.id_mtb16)
order by a.name_mtb16, a.id_mtb16, a.ak2_name;

create table hot_pt."06_tab_union_arten_lang_ohne boden_less30"
AS select * from hot_pt."06_union_arten_lang_ohne boden_less30";

create index "06_tab_union_arten_lang_ohne boden_less30_btree" on hot_pt."06_tab_union_arten_lang_ohne boden_less30"
(name_mtb16,
id_mtb16,
ak2,
ak2_name,
anz_wel,
quelle,
wel,
welprio);


--A07: short version of step A06 with distinct values per grid cell

CREATE OR REPLACE VIEW hot_pt."07_pt_arten_ohne boden_less30"
 AS
select distinct (b.name_mtb16||b.id_mtb16) as mtb_id, b.ak2, b.ak2_name, b.anz_wel, b.quelle, b.wel, b.welprio,
b.form1, b.form2, b.form3, b.form4, b.form5, b.form6, b.form7, b.form8, b.form9, b.form10,
b.form11, b.form12, b.form13, b.form14, b.form15, b.form16, b.form17, b.form18, b.form19, b.form20,
b.form21, b.form22, b.form23, b.form24
from hot_pt."06_tab_union_arten_lang_ohne boden_less30" b
order by mtb_id, ak2_name;

create table hot_pt."07_tab_pt_arten_ohne boden_less30"
AS select * from hot_pt."07_pt_arten_ohne boden_less30";

create index "07_tab_pt_arten_ohne boden_less30_btree" on hot_pt."07_tab_pt_arten_ohne boden_less30"
( mtb_id, ak2, ak2_name, anz_wel, quelle, wel, welprio);


--B01: union all polygon and line data sets, harmonize the columns
```

```sql

create table hot_fl."01_tab_union_all_t1"
AS
        select cast (id as varchar) as id, geom, name as species, null as species_joinfield, 'd_vegpg' as quelle, null as daten_id ,null as daten_art
        from hot_fl.d_vegpg
union all
        select distinct a.id, a.geom, b.artname1 as species, a.biotop as species_joinfield, 'by_alppg' as quelle, null as daten_id ,null as daten_art
        from hot_fl.by_alppg a
        left join hot_fl.by_alppg_arten b on a.biotop = b.biotop
        where b.artname1 is not null
union all
        select distinct a.id, a.geom, b.artname1 as species, a.biotop as species_joinfield, 'by_flapg' as quelle, null as daten_id ,null as daten_art
        from hot_fl.by_flapg a
        left join hot_fl.by_flapg_arten b on a.biotop = b.biotop
        where b.artname1 is not null
union all
        select distinct a.id, a.geom, b.artname1 as species, a.biotop as species_joinfield, 'by_stapg' as quelle, null as daten_id ,null as daten_art
        from hot_fl.by_stapg a
        left join hot_fl.by_stapg_arten b on a.biotop = b.biotop
        where b.artname1 is not null
union all
        select distinct a.id, a.geom, b.artname1 as species, a.biotop as species_joinfield, 'by_lrtpg' as quelle, null as daten_id ,null as daten_art
        from hot_fl.by_lrtpg a
        left join hot_fl.by_lrtpg_arten b on a.biotop = b.biotop
        where b.artname1 is not null
union all
        select distinct cast (a.id as varchar) as id, a.geom, c.artname as species, a.giscode as species_joinfield, 'mv_pg' as quelle, a.alt_lfd_nr as daten_id , null as daten_art
        from hot_fl.mv_pg a
        left join hot_fl.mv_pg_mvbio b on a.giscode = b.giscode
        left join hot_fl.mv_pg_mvbiopfl c on b.id = c.id_mvbio
        where c.artname is not null
union all
        select cast (id as varchar) as id, geom, wel_name as species, null as species_joinfield, 'nrw_fpln' as quelle, kennung as daten_id , pfl_wis as daten_art
        from hot_fl.nrw_fpln
union all
        select cast (id as varchar) as id, geom, wel_name as species, null as species_joinfield, 'nrw_btprioln' as quelle, kennung as daten_id , pfl_wis as daten_art
        from hot_fl.nrw_btprioln
union all
        select cast (id as varchar) as id, geom, wel_name as species, null as species_joinfield, 'nrw_vawelpg' as quelle, kennung as daten_id , pfl_wis as daten_art
        from hot_fl.nrw_vawelpg
union all
        select cast (id as varchar) as id, geom, wel_name as species, null as species_joinfield, 'nrw_vapriopg' as quelle, kennung as daten_id , pfl_wis as daten_art
        from hot_fl.nrw_vapriopg
union all
        select cast (id as varchar) as id, geom, wel_name as species, null as species_joinfield, 'nrw_fppg' as quelle, kennung as daten_id , pfl_wis as daten_art
        from hot_fl.nrw_fppg
union all
        select cast (id as varchar) as id, geom, wel_name as species, null as species_joinfield, 'nrw_btw2pg' as quelle, kennung as daten_id , pfl_wis as daten_art
        from hot_fl.nrw_btw2pg
union all
        select cast (id as varchar) as id, geom, wel_name as species, null as species_joinfield, 'nrw_btw1pg' as quelle, kennung as daten_id , pfl_wis as daten_art
        from hot_fl.nrw_btw1pg
```

```sql
union all
        select cast (id as varchar) as id, geom, wel_name as species, null as species_joinfield, 'nrw_btpriopg' as quelle, kennung as daten_id , pfl_wis as daten_art
        from hot_fl.nrw_btpriopg
union all
        select cast (id as varchar) as id, geom, art_wiss as species, null as species_joinfield, 'rlp_ln' as quelle, null as daten_id , null as daten_art
        from hot_fl.rlp_ln
union all
        select cast (id as varchar) as id, geom, art_wiss as species, null as species_joinfield, 'rlp_pg' as quelle, null as daten_id , null as daten_art
        from hot_fl.rlp_pg
union all
        select cast (id as varchar) as id, geom, 'Achillea ptarmica' as species, null as species_joinfield, 'sl_achptapg' as quelle, objektname as daten_id , null as daten_art
        from hot_fl.sl_achptapg
union all
        select cast (id as varchar) as id, geom, 'Arum maculatum' as species, null as species_joinfield, 'sl_arumacpg' as quelle, objektname as daten_id , null as daten_art
        from hot_fl.sl_arumacpg
union all
        select cast (id as varchar) as id, geom, 'Asparagus officinalis' as species, null as species_joinfield, 'sl_aspoffpg' as quelle, objektname as daten_id , null as daten_art
        from hot_fl.sl_aspoffpg
union all
        select cast (id as varchar) as id, geom, 'Carum carvi' as species, null as species_joinfield, 'sl_carcarpg' as quelle, objektname as daten_id , null as daten_art
        from hot_fl.sl_carcarpg
union all
        select cast (id as varchar) as id, geom, 'Festuca brevipila' as species, null as species_joinfield, 'sl_fesbrepg' as quelle, objektname as daten_id , null as daten_art
        from hot_fl.sl_fesbrepg
union all
        select cast (id as varchar) as id, geom, 'Festuca filiformis' as species, null as species_joinfield, 'sl_fesfilpg' as quelle, objektname as daten_id , null as daten_art
        from hot_fl.sl_fesfilpg
union all
        select cast (id as varchar) as id, geom, 'Festuca guestfalica' as species, null as species_joinfield, 'sl_fesguepg' as quelle, objektname as daten_id , null as daten_art
        from hot_fl.sl_fesguepg
union all
        select cast (id as varchar) as id, geom, 'Festuca pratensis' as species, null as species_joinfield, 'sl_fesprapg' as quelle, objektname as daten_id , null as daten_art
        from hot_fl.sl_fesprapg
union all
        select cast (id as varchar) as id, geom, 'Hypericum hirsutum' as species, null as species_joinfield, 'sl_hyphirpg' as quelle, objektname as daten_id , null as daten_art
        from hot_fl.sl_hyphirpg
union all
        select cast (id as varchar) as id, geom, 'Hypericum humifusum' as species, null as species_joinfield, 'sl_hyphumpg' as quelle, objektname as daten_id , null as daten_art
        from hot_fl.sl_hyphumpg
union all
        select cast (id as varchar) as id, geom, 'Hypericum perforatum' as species, null as species_joinfield, 'sl_hypperpg' as quelle, objektname as daten_id , null as daten_art
        from hot_fl.sl_hypperpg
union all
        select cast (id as varchar) as id, geom, 'Prunus mahaleb' as species, null as species_joinfield, 'sl_prumahpg' as quelle, objektname as daten_id , null as daten_art
        from hot_fl.sl_prumahpg
```

```
union all
        select cast (id as varchar) as id, geom, 'Pyrus pyraster' as species, null as
        species_joinfield, 'sl_pyrpyrpg' as quelle, objektname as daten_id , null as
        daten_art
        from hot_fl.sl_pyrpyrpg
union all
        select cast (id as varchar) as id, geom, 'Thymus praecox' as species, null as
        species_joinfield, 'sl_thyprapg' as quelle, objektname as daten_id , null as
        daten_art
        from hot_fl.sl_thyprapg
union all
        select cast (id as varchar) as id, geom, 'Trifolium campestre' as species,
        null as species_joinfield, 'sl_tricampg' as quelle, objektname as daten_id ,
        null as daten_art
        from hot_fl.sl_tricampg;

create table hot_fl."01_tab_union_all_t2"
as
        select distinct cast (id as varchar) as id, a.geom, b.art as species, a.
        lrt_guid as species_joinfield, 'st_flpg' as quelle, null as daten_id ,null as
        daten_art
        from hot_fl.st_flpg a
        left join hot_fl.st_arten b on a.lrt_guid = b.ref_guid
        where b.art is not null and b.taxnr <> '' and a.geom is not null
union all
        select distinct cast (id as varchar) as id, a.geom, b.art as species, a.
        lrt_guid as species_joinfield, 'st_lipg' as quelle, null as daten_id ,null as
        daten_art
        from hot_fl.st_lipg a
        left join hot_fl.st_arten b on a.lrt_guid = b.ref_guid
        where b.art is not null and b.taxnr <> '' and a.geom is not null
union all
        select distinct cast (a.id as varchar) as id, a.geom, c.name as species, a.
        pk_ident as species_joinfield, 'bb_flpg' as quelle, null as daten_id ,null as
        daten_art
        from hot_fl.bb_flpg a
        left join hot_fl.bb_dflora b on a.pk_ident = b.fk_ident
        left join hot_fl.bb_bbk_arten c on b.fk_arten = c.pk_artcode
        where c.name is not null
union all
        select distinct cast (a.id as varchar) as id, a.geom, c.name as species, a.
        pk_ident as species_joinfield, 'bb_lipg' as quelle, null as daten_id ,null as
        daten_art
        from hot_fl.bb_lipg a
        left join hot_fl.bb_dflora b on a.pk_ident = b.fk_ident
        left join hot_fl.bb_bbk_arten c on b.fk_arten = c.pk_artcode
        where c.name is not null
union all
        select distinct cast (a.id as varchar) as id, a.geom, c.art_wissenschaftl as
        species, null as species_joinfield, 'sa_lipg' as quelle, cast (a.lff_id as
        varchar) as daten_id ,null as daten_art
        from hot_fl.sa_lipg a
        left join hot_fl.sa_vak_arten b on cast (a.lff_id as varchar) = b.lff_id
        left join hot_fl.sa_arten c on b.vak_id = c.vak_id
        where c.art_wissenschaftl is not null
union all
        select distinct cast (a.id as varchar) as id, a.geom, c.art_wissenschaftl as
        species, null as species_joinfield, 'sa_flpg' as quelle, cast (a.lff_id as
        varchar) as daten_id ,null as daten_art
        from hot_fl.sa_flpg a
        left join hot_fl.sa_vak_arten b on cast (a.lff_id as varchar) = b.lff_id
        left join hot_fl.sa_arten c on b.vak_id = c.vak_id
        where c.art_wissenschaftl is not null
union all
        select distinct cast (a.id as varchar) as id, a.geom, b.pflanzenart as species
        , null as species_joinfield, 'rlp_lanispg2023' as quelle, a.kennung as
        daten_id ,null as daten_art
        from hot_fl.rlp_lanispg2023 a
        left join hot_fl.rlp_lanis_arten b on a.kennung = b.kennung
        where b.pflanzenart is not null and a.geom is not null
union all
        select distinct cast (a.id as varchar) as id, a.geom, b.pflanzenart as species
        , null as species_joinfield, 'rlp_ffhpg' as quelle, a.name as daten_id ,null
```

```sql
            as daten_art
            from hot_fl.rlp_ffhpg a
            left join hot_fl.rlp_lanis_arten b on a.name = b.kennung
            where b.pflanzenart is not null and a.geom is not null
union all
            select distinct cast (a.id as varchar) as id, a.geom, b.pflanzenart as species
            , null as species_joinfield, 'rlp_lrtpg' as quelle, a.name as daten_id ,null
            as daten_art
            from hot_fl.rlp_lrtpg a
            left join hot_fl.rlp_lanis_arten b on a.name = b.kennung
            where b.pflanzenart is not null and a.geom is not null
union all
            select distinct cast (a.id as varchar) as id, a.geom, b.pflanzenart as species
            , null as species_joinfield, 'rlp_lanislipg' as quelle, a.kennung as daten_id
            ,null as daten_art
            from hot_fl.rlp_lanislipg a
            left join hot_fl.rlp_lanis_arten b on a.kennung = b.kennung
            where b.pflanzenart is not null
union all
            select cast (id as varchar) as id, geom, wel_name as species, null as
            species_joinfield, 'nrw_btwelln' as quelle, kennung as daten_id , pfl_wis as
            daten_art
            from hot_fl.nrw_btwelln
union all
            select distinct cast (a.id as varchar) as id, a.geom, b.art as species,
            "Biotop/LRT-Nr." as species_joinfield, 'th_bkpg' as quelle, cast (a.btp_id as
            varchar) as daten_id, null as daten_art
            from hot_fl.th_bkpg a
            left join hot_fl.th_bk_arten b on a.biotopnr = b."Biotop/LRT-Nr."
            where b.art is not null
union all
            select distinct cast (a.id as varchar) as id, a.geom, b.art as species,
            "Biotop/LRT-Nr." as species_joinfield, 'th_lrtpg' as quelle, cast (a.btp_id as
             varchar) as daten_id, null as daten_art
            from hot_fl.th_lrtpg a
            left join hot_fl.th_lrt_arten b on a.biotopnr = b."Biotop/LRT-Nr."
            where b.art is not null
union all
            select distinct a.id, a.geom, b.art_wi as species, a.gebiets_nr as
            species_joinfield, 'ni_niwappg' as quelle, b.am_nr as daten_id ,null as
            daten_art
            from hot_fl.ni_niwappg a
            left join hot_fl.ni_niwap_arten b on a.gebiets_nr = b.geb_nr
            where b.art_wi is not null;

ALTER TABLE hot_fl."01_tab_union_all_t1"
    ADD CONSTRAINT pkey_01_tab_union PRIMARY KEY (id, species, quelle);

CREATE INDEX union_tab_idx
                ON hot_fl."01_tab_union_all_t1"
                USING GIST (geom);

ALTER TABLE hot_fl."01_tab_union_all_t2"
    ADD CONSTRAINT pkey_01_tab_union2 PRIMARY KEY (id, species, quelle);

CREATE INDEX union_tab_idx
                ON hot_fl."01_tab_union_all_t2"
                USING GIST (geom);


--B01-02: calculate area (polygon data sets) and lenght (line data sets)

create table hot_fl."01_tab_union_t1_arealen"
as
select a.*, st_area (a."geom") as area, st_length (a."geom") as len
from hot_fl."01_tab_union_all_t1" a
order by area, len;

create table hot_fl."01_tab_union_t2_arealen"
as
select a.*, st_area (a."geom") as area, st_length (a."geom") as len
from hot_fl."01_tab_union_all_t2" a
order by area, len;
```

```
--B01-03: remove too big and too long geometries

create table hot_fl."01_tab_union_t1_amax"
as
select a.*
from hot_fl."01_tab_union_t1_arealen" a
where area < 8099951.240394007  ; --squaremeters

create table hot_fl."01_tab_union_t2_amax"
as
select a.*
from hot_fl."01_tab_union_t2_arealen" a
where area < 8099951.240394007  ; --squaremeters

create table hot_fl."01_tab_union_t1"
as
select a.*
from hot_fl."01_tab_union_t1_amax" a
where len < 4027.995124283213   ; -- meters

create table hot_fl."01_tab_union_t2"
as
select a.*
from hot_fl."01_tab_union_t2_amax" a
where len < 4027.995124283213   ; -- meters

--B01-04: union both partial steps

create table hot_fl."01_tab_union_t12"
as
select * from hot_fl."01_tab_union_t1"
union all
select * from hot_fl."01_tab_union_t2";


--B02: reduce line and area geometries to a central point in the geometry (creating a point shape)

CREATE OR REPLACE VIEW hot_fl."02_union_pt"
  AS
 select a.*, st_pointonsurface(a."geom") as geom_pt
 from hot_fl."01_tab_union_t12" a;

create table hot_fl."02_tab_union_pt"
AS
select geom_pt, id, species, species_joinfield, quelle, daten_id, daten_art
from hot_fl."02_union_pt";

ALTER TABLE hot_fl."02_tab_union_pt"
    ADD CONSTRAINT pkey_02_tab_union_pt PRIMARY KEY (id, species, quelle);


--B03: separate the taxonomic name in genus and species name

CREATE OR REPLACE VIEW hot_fl."03_01_pos1"
 AS
   SELECT a.*, "position"(a.species, ' ') AS pos1
   FROM hot_fl."02_tab_union_pt" a;

CREATE OR REPLACE VIEW hot_fl."03_02_gat"
 AS
 SELECT a.*,
    "substring"(a.species::text, 1, a.pos1 - 1) AS gat,
    "substring"(a.species::text, a.pos1 + 1, 25) AS name_min_gat
   FROM hot_fl."03_01_pos1" a
   where pos1 <> '0';

CREATE OR REPLACE VIEW hot_fl."03_03_pos2"
 AS
 SELECT a.*,
    "position"(a.name_min_gat, ' ') AS pos2
   FROM hot_fl."03_02_gat" a;
```

```
CREATE OR REPLACE VIEW hot_fl."03_04_gatart"
 AS
  SELECT a.*,
        CASE
            WHEN a.pos2 = 0 THEN a.name_min_gat
            ELSE "substring"(a.name_min_gat, 1, a.pos2 - 1)
        END AS art
   FROM hot_fl."03_03_pos2" a;


--B04: join CWR information to the point shape using genus and species names as joins fields

CREATE OR REPLACE VIEW hot_fl."04_union_taxwel"
 AS
 select a.*, b.ak2, b.name as ak2_name, b.wel, b.welprio, b.form1, b.form2, b.form3, b.form4, b.form5, b.form6, b.form7, b.form8, b.form9, b.form10,
b.form11, b.form12, b.form13, b.form14, b.form15, b.form16, b.form17, b.form18, b.form19, b.form20,
b.form21, b.form22, b.form23, b.form24
from hot_fl."03_04_gatart" a
left join hot_fl.welliste b on (a.gat||' '||a.art) = (b.gat||' '||b.art)
where b.ak2 is not null;

create table hot_fl."04_tab_union_taxwel"
AS
select *
from hot_fl."04_union_taxwel"
order by ak2_name;

create table hot_fl."04_tab_union_taxwel_dist" -- reduce to distinct values
AS
select distinct *
from hot_fl."04_tab_union_taxwel";

ALTER TABLE hot_fl."04_tab_union_taxwel_dist"
    ADD CONSTRAINT pkey_04_tab_union_dist PRIMARY KEY (id, species, quelle, ak2, ak2_name);

CREATE INDEX union_tab_idx_dist
                  ON hot_fl."04_tab_union_taxwel_dist"
                  USING GIST (geom_pt);


--B05: join grid cell shape to point shape

CREATE OR REPLACE VIEW hot_fl."05_union_mtb16"
 AS
select distinct a.*, b.id as id_mtb16, b.name as name_mtb16, b.geom as geom_mtb16
from hot_fl."04_tab_union_taxwel_dist" a
left join hot_fl.mtb16 b on st_within(a.geom_pt, b.geom)
where b.geom is not null;

create table hot_fl."05_tab_union_mtb16"
AS
select distinct *
from hot_fl."05_union_mtb16";

ALTER TABLE hot_fl."05_tab_union_mtb16"
    ADD CONSTRAINT pkey_05_tab_union_mtb16 PRIMARY KEY (id, species, quelle, ak2, ak2_name, id_mtb16, name_mtb16);


--B06: create species list including the assignment between species and grid cells (long version)

CREATE OR REPLACE VIEW hot_fl."06_union_arten_lang"
 AS
select distinct a.*, c.count as anz_wel
from hot_fl."05_tab_union_mtb16" a
right join (select b.name_mtb16, b.id_mtb16, count (distinct b.ak2) from hot_fl."05_tab_union_mtb16" b group by b.name_mtb16, b.id_mtb16) c
```

```
on (a.name_mtb16||a.id_mtb16) = (c.name_mtb16||c.id_mtb16)
order by a.name_mtb16, a.id_mtb16, a.ak2_name;


--B07: short version of step B06 with distinct values per grid cell

CREATE OR REPLACE VIEW hot_fl."07_fl_arten"
 AS
select distinct (b.name_mtb16||b.id_mtb16) as mtb_id, b.ak2, b.ak2_name, b.anz_wel, b.quelle, b.wel, b.welprio,
b.form1, b.form2, b.form3, b.form4, b.form5, b.form6, b.form7, b.form8, b.form9, b.form10,
b.form11, b.form12, b.form13, b.form14, b.form15, b.form16, b.form17, b.form18, b.form19, b.form20,
b.form21, b.form22, b.form23, b.form24
from hot_fl."06_union_arten_lang" b
order by mtb_id, ak2_name;

create table hot_fl."07_tab_fl_arten"
AS
select *
from hot_fl."07_fl_arten";

ALTER TABLE hot_fl."07_tab_fl_arten"
    ADD CONSTRAINT pkey_07_tab_fl_arten PRIMARY KEY (mtb_id, ak2, ak2_name,quelle);


--C01: union species lists of the point, polygon and line data sets, harmonize the columns

create table hot_fl."09_tab_arten_pt_fl_ohne boden_less30"
as
        select
        mtb_id, ak2, ak2_name, cast (anz_wel as bigint) as anz_wel, quelle, wel, welprio, form1, form2, form3, form4, form5,
        form6, form7, form8, form9, form10, form11, form12, form13, form14, form15,
        form16, form17, form18, form19, form20, form21, form22, form23, form24
        from hot_pt."07_tab_pt_arten_ohne boden_less30"
union all
        select
        cast (mtb_id as varchar) as mtb_id, ak2, ak2_name, anz_wel, cast (quelle as varchar) as quelle, wel, welprio, form1, form2, form3, form4, form5,
        form6, form7, form8, form9, form10, form11, form12, form13, form14, form15,
        form16, form17, form18, form19, form20, form21, form22, form23, form24
        from hot_fl."07_tab_fl_arten";

ALTER TABLE hot_fl."09_tab_arten_pt_fl_ohne boden_less30"
ADD COLUMN ID SERIAL PRIMARY KEY;

create index "09_tab_arten_pt_fl_ohne boden_less30_btree" on hot_fl."09_tab_arten_pt_fl_ohne boden_less30"
( mtb_id, welprio);


--C02: export species list
-- export via QGIS as geopackage


--C03: counting species per grid cell (CWR and priority CWR per vegetation type; only focus and main occurrence)

CREATE OR REPLACE VIEW hot_fl."10_pt_fl_count_ohne boden_less30_onv"
 AS
select distinct b.mtb_id, wel.count as wel, wp.count as welprio,
f1.count as wel_f1, f2.count as wel_f2,f3.count as wel_f3, f4.count as wel_f4, f5.count as wel_f5,
f6.count as wel_f6, f7.count as wel_f7, f8.count as wel_f8, f9.count as wel_f9, f10.count as wel_f10,
f11.count as wel_f11, f12.count as wel_f12, f13.count as wel_f13, f14.count as wel_f14, f15.count as wel_f15,
f16.count as wel_f16, f17.count as wel_f17, f18.count as wel_f18, f19.count as wel_f19, f20.count as wel_f20,
f21.count as wel_f21, f22.count as wel_f22, f23.count as wel_f23, f24.count as wel_f24
```

```
,

fp1.count as prio_f1, fp2.count as prio_f2,fp3.count as prio_f3, fp4.count as prio_f4,
 fp5.count as prio_f5,
fp6.count as prio_f6, fp7.count as prio_f7, fp8.count as prio_f8, fp9.count as prio_f9
, fp10.count as prio_f10,
fp11.count as prio_f11, fp12.count as prio_f12, fp13.count as prio_f13, fp14.count as
prio_f14, fp15.count as prio_f15,
fp16.count as prio_f16, fp17.count as prio_f17, fp18.count as prio_f18, fp19.count as
prio_f19, fp20.count as prio_f20,
fp21.count as prio_f21, fp22.count as prio_f22, fp23.count as prio_f23, fp24.count as
prio_f24

from hot_fl."09_tab_arten_pt_fl_ohne boden_less30" b
left join (select c.mtb_id, count (distinct c."ak2") from hot_fl.
"09_tab_arten_pt_fl_ohne boden_less30" c group by c.mtb_id) wel on (wel.mtb_id) = (b.
mtb_id)
left join (select c.mtb_id, count (distinct c."ak2") from hot_fl.
"09_tab_arten_pt_fl_ohne boden_less30" c where c."welprio" = 'x' group by c.mtb_id) wp
 on (wp.mtb_id) = (b.mtb_id)
left join (select c.mtb_id, count (distinct c."ak2") from hot_fl.
"09_tab_arten_pt_fl_ohne boden_less30" c where c."form1" = '2' or c."form1" = '3'
group by c.mtb_id) f1 on (f1.mtb_id) = (b.mtb_id)
left join (select c.mtb_id, count (distinct c."ak2") from hot_fl.
"09_tab_arten_pt_fl_ohne boden_less30" c where c."form2" = '2' or c."form2" = '3'
group by c.mtb_id) f2 on (f2.mtb_id) = (b.mtb_id)
left join (select c.mtb_id, count (distinct c."ak2") from hot_fl.
"09_tab_arten_pt_fl_ohne boden_less30" c where c."form3" = '2' or c."form3" = '3'
group by c.mtb_id) f3 on (f3.mtb_id) = (b.mtb_id)
left join (select c.mtb_id, count (distinct c."ak2") from hot_fl.
"09_tab_arten_pt_fl_ohne boden_less30" c where c."form4" = '2' or c."form4" = '3'
group by c.mtb_id) f4 on (f4.mtb_id) = (b.mtb_id)
left join (select c.mtb_id, count (distinct c."ak2") from hot_fl.
"09_tab_arten_pt_fl_ohne boden_less30" c where c."form5" = '2' or c."form5" = '3'
group by c.mtb_id) f5 on (f5.mtb_id) = (b.mtb_id)
left join (select c.mtb_id, count (distinct c."ak2") from hot_fl.
"09_tab_arten_pt_fl_ohne boden_less30" c where c."form6" = '2' or c."form6" = '3'
group by c.mtb_id) f6 on (f6.mtb_id) = (b.mtb_id)
left join (select c.mtb_id, count (distinct c."ak2") from hot_fl.
"09_tab_arten_pt_fl_ohne boden_less30" c where c."form7" = '2' or c."form7" = '3'
group by c.mtb_id) f7 on (f7.mtb_id) = (b.mtb_id)
left join (select c.mtb_id, count (distinct c."ak2") from hot_fl.
"09_tab_arten_pt_fl_ohne boden_less30" c where c."form8" = '2' or c."form8" = '3'
group by c.mtb_id) f8 on (f8.mtb_id) = (b.mtb_id)
left join (select c.mtb_id, count (distinct c."ak2") from hot_fl.
"09_tab_arten_pt_fl_ohne boden_less30" c where c."form9" = '2' or c."form9" = '3'
group by c.mtb_id) f9 on (f9.mtb_id) = (b.mtb_id)
left join (select c.mtb_id, count (distinct c."ak2") from hot_fl.
"09_tab_arten_pt_fl_ohne boden_less30" c where c."form10" = '2' or c."form10" = '3'
group by c.mtb_id) f10 on (f10.mtb_id) = (b.mtb_id)
left join (select c.mtb_id, count (distinct c."ak2") from hot_fl.
"09_tab_arten_pt_fl_ohne boden_less30" c where c."form11" = '2' or c."form11" = '3'
group by c.mtb_id) f11 on (f11.mtb_id) = (b.mtb_id)
left join (select c.mtb_id, count (distinct c."ak2") from hot_fl.
"09_tab_arten_pt_fl_ohne boden_less30" c where c."form12" = '2' or c."form12" = '3'
group by c.mtb_id) f12 on (f12.mtb_id) = (b.mtb_id)
left join (select c.mtb_id, count (distinct c."ak2") from hot_fl.
"09_tab_arten_pt_fl_ohne boden_less30" c where c."form13" = '2' or c."form13" = '3'
group by c.mtb_id) f13 on (f13.mtb_id) = (b.mtb_id)
left join (select c.mtb_id, count (distinct c."ak2") from hot_fl.
"09_tab_arten_pt_fl_ohne boden_less30" c where c."form14" = '2' or c."form14" = '3'
group by c.mtb_id) f14 on (f14.mtb_id) = (b.mtb_id)
left join (select c.mtb_id, count (distinct c."ak2") from hot_fl.
"09_tab_arten_pt_fl_ohne boden_less30" c where c."form15" = '2' or c."form15" = '3'
group by c.mtb_id) f15 on (f15.mtb_id) = (b.mtb_id)
left join (select c.mtb_id, count (distinct c."ak2") from hot_fl.
"09_tab_arten_pt_fl_ohne boden_less30" c where c."form16" = '2' or c."form16" = '3'
group by c.mtb_id) f16 on (f16.mtb_id) = (b.mtb_id)
left join (select c.mtb_id, count (distinct c."ak2") from hot_fl.
"09_tab_arten_pt_fl_ohne boden_less30" c where c."form17" = '2' or c."form17" = '3'
group by c.mtb_id) f17 on (f17.mtb_id) = (b.mtb_id)
left join (select c.mtb_id, count (distinct c."ak2") from hot_fl.
"09_tab_arten_pt_fl_ohne boden_less30" c where c."form18" = '2' or c."form18" = '3'
```

```
group by c.mtb_id) f18 on (f18.mtb_id) = (b.mtb_id)
left join (select c.mtb_id, count (distinct c."ak2") from hot_fl.
"09_tab_arten_pt_fl_ohne boden_less30" c where c."form19" = '2' or c."form19" = '3'
group by c.mtb_id) f19 on (f19.mtb_id) = (b.mtb_id)
left join (select c.mtb_id, count (distinct c."ak2") from hot_fl.
"09_tab_arten_pt_fl_ohne boden_less30" c where c."form20" = '2' or c."form20" = '3'
group by c.mtb_id) f20 on (f20.mtb_id) = (b.mtb_id)
left join (select c.mtb_id, count (distinct c."ak2") from hot_fl.
"09_tab_arten_pt_fl_ohne boden_less30" c where c."form21" = '2' or c."form21" = '3'
group by c.mtb_id) f21 on (f21.mtb_id) = (b.mtb_id)
left join (select c.mtb_id, count (distinct c."ak2") from hot_fl.
"09_tab_arten_pt_fl_ohne boden_less30" c where c."form22" = '2' or c."form22" = '3'
group by c.mtb_id) f22 on (f22.mtb_id) = (b.mtb_id)
left join (select c.mtb_id, count (distinct c."ak2") from hot_fl.
"09_tab_arten_pt_fl_ohne boden_less30" c where c."form23" = '2' or c."form23" = '3'
group by c.mtb_id) f23 on (f23.mtb_id) = (b.mtb_id)
left join (select c.mtb_id, count (distinct c."ak2") from hot_fl.
"09_tab_arten_pt_fl_ohne boden_less30" c where c."form24" = '2' or c."form24" = '3'
group by c.mtb_id) f24 on (f24.mtb_id) = (b.mtb_id)

left join (select c.mtb_id, count (distinct c."ak2") from hot_fl.
"09_tab_arten_pt_fl_ohne boden_less30" c where (c."form1" = '2' or c."form1" = '3' )
and c.welprio = 'x' group by c.mtb_id) fp1 on (fp1.mtb_id) = (b.mtb_id)
left join (select c.mtb_id, count (distinct c."ak2") from hot_fl.
"09_tab_arten_pt_fl_ohne boden_less30" c where (c."form2" = '2' or c."form2" = '3' )
and c.welprio = 'x' group by c.mtb_id) fp2 on (fp2.mtb_id) = (b.mtb_id)
left join (select c.mtb_id, count (distinct c."ak2") from hot_fl.
"09_tab_arten_pt_fl_ohne boden_less30" c where (c."form3" = '2' or c."form3" = '3' )
and c.welprio = 'x' group by c.mtb_id) fp3 on (fp3.mtb_id) = (b.mtb_id)
left join (select c.mtb_id, count (distinct c."ak2") from hot_fl.
"09_tab_arten_pt_fl_ohne boden_less30" c where (c."form4" = '2' or c."form4" = '3' )
and c.welprio = 'x' group by c.mtb_id) fp4 on (fp4.mtb_id) = (b.mtb_id)
left join (select c.mtb_id, count (distinct c."ak2") from hot_fl.
"09_tab_arten_pt_fl_ohne boden_less30" c where (c."form5" = '2' or c."form5" = '3' )
and c.welprio = 'x' group by c.mtb_id) fp5 on (fp5.mtb_id) = (b.mtb_id)
left join (select c.mtb_id, count (distinct c."ak2") from hot_fl.
"09_tab_arten_pt_fl_ohne boden_less30" c where (c."form6" = '2' or c."form6" = '3' )
and c.welprio = 'x' group by c.mtb_id) fp6 on (fp6.mtb_id) = (b.mtb_id)
left join (select c.mtb_id, count (distinct c."ak2") from hot_fl.
"09_tab_arten_pt_fl_ohne boden_less30" c where (c."form7" = '2' or c."form7" = '3' )
and c.welprio = 'x' group by c.mtb_id) fp7 on (fp7.mtb_id) = (b.mtb_id)
left join (select c.mtb_id, count (distinct c."ak2") from hot_fl.
"09_tab_arten_pt_fl_ohne boden_less30" c where (c."form8" = '2' or c."form8" = '3' )
and c.welprio = 'x' group by c.mtb_id) fp8 on (fp8.mtb_id) = (b.mtb_id)
left join (select c.mtb_id, count (distinct c."ak2") from hot_fl.
"09_tab_arten_pt_fl_ohne boden_less30" c where (c."form9" = '2' or c."form9" = '3' )
and c.welprio = 'x' group by c.mtb_id) fp9 on (fp9.mtb_id) = (b.mtb_id)
left join (select c.mtb_id, count (distinct c."ak2") from hot_fl.
"09_tab_arten_pt_fl_ohne boden_less30" c where (c."form10" = '2' or c."form10" = '3' )
 and c.welprio = 'x' group by c.mtb_id) fp10 on (fp10.mtb_id) = (b.mtb_id)
left join (select c.mtb_id, count (distinct c."ak2") from hot_fl.
"09_tab_arten_pt_fl_ohne boden_less30" c where (c."form11" = '2' or c."form11" = '3' )
 and c.welprio = 'x' group by c.mtb_id) fp11 on (fp11.mtb_id) = (b.mtb_id)
left join (select c.mtb_id, count (distinct c."ak2") from hot_fl.
"09_tab_arten_pt_fl_ohne boden_less30" c where (c."form12" = '2' or c."form12" = '3' )
 and c.welprio = 'x' group by c.mtb_id) fp12 on (fp12.mtb_id) = (b.mtb_id)
left join (select c.mtb_id, count (distinct c."ak2") from hot_fl.
"09_tab_arten_pt_fl_ohne boden_less30" c where (c."form13" = '2' or c."form13" = '3' )
 and c.welprio = 'x' group by c.mtb_id) fp13 on (fp13.mtb_id) = (b.mtb_id)
left join (select c.mtb_id, count (distinct c."ak2") from hot_fl.
"09_tab_arten_pt_fl_ohne boden_less30" c where (c."form14" = '2' or c."form14" = '3' )
 and c.welprio = 'x' group by c.mtb_id) fp14 on (fp14.mtb_id) = (b.mtb_id)
left join (select c.mtb_id, count (distinct c."ak2") from hot_fl.
"09_tab_arten_pt_fl_ohne boden_less30" c where (c."form15" = '2' or c."form15" = '3' )
 and c.welprio = 'x' group by c.mtb_id) fp15 on (fp15.mtb_id) = (b.mtb_id)
left join (select c.mtb_id, count (distinct c."ak2") from hot_fl.
"09_tab_arten_pt_fl_ohne boden_less30" c where (c."form16" = '2' or c."form16" = '3' )
 and c.welprio = 'x' group by c.mtb_id) fp16 on (fp16.mtb_id) = (b.mtb_id)
left join (select c.mtb_id, count (distinct c."ak2") from hot_fl.
"09_tab_arten_pt_fl_ohne boden_less30" c where (c."form17" = '2' or c."form17" = '3' )
 and c.welprio = 'x' group by c.mtb_id) fp17 on (fp17.mtb_id) = (b.mtb_id)
left join (select c.mtb_id, count (distinct c."ak2") from hot_fl.
"09_tab_arten_pt_fl_ohne boden_less30" c where (c."form18" = '2' or c."form18" = '3' )
```

```sql
 and c.welprio = 'x' group by c.mtb_id) fp18 on (fp18.mtb_id) = (b.mtb_id)
left join (select c.mtb_id, count (distinct c."ak2") from hot_fl.
"09_tab_arten_pt_fl_ohne boden_less30" c where (c."form19" = '2' or c."form19" = '3' )
 and c.welprio = 'x' group by c.mtb_id) fp19 on (fp19.mtb_id) = (b.mtb_id)
left join (select c.mtb_id, count (distinct c."ak2") from hot_fl.
"09_tab_arten_pt_fl_ohne boden_less30" c where (c."form20" = '2' or c."form20" = '3' )
 and c.welprio = 'x' group by c.mtb_id) fp20 on (fp20.mtb_id) = (b.mtb_id)
left join (select c.mtb_id, count (distinct c."ak2") from hot_fl.
"09_tab_arten_pt_fl_ohne boden_less30" c where (c."form21" = '2' or c."form21" = '3' )
 and c.welprio = 'x' group by c.mtb_id) fp21 on (fp21.mtb_id) = (b.mtb_id)
left join (select c.mtb_id, count (distinct c."ak2") from hot_fl.
"09_tab_arten_pt_fl_ohne boden_less30" c where (c."form22" = '2' or c."form22" = '3' )
 and c.welprio = 'x' group by c.mtb_id) fp22 on (fp22.mtb_id) = (b.mtb_id)
left join (select c.mtb_id, count (distinct c."ak2") from hot_fl.
"09_tab_arten_pt_fl_ohne boden_less30" c where (c."form23" = '2' or c."form23" = '3' )
 and c.welprio = 'x' group by c.mtb_id) fp23 on (fp23.mtb_id) = (b.mtb_id)
left join (select c.mtb_id, count (distinct c."ak2") from hot_fl.
"09_tab_arten_pt_fl_ohne boden_less30" c where (c."form24" = '2' or c."form24" = '3' )
 and c.welprio = 'x' group by c.mtb_id) fp24 on (fp24.mtb_id) = (b.mtb_id)
order by mtb_id;


--C04: add geometries of the grid cells

CREATE OR REPLACE VIEW hot_fl."11_ptfl_count_mtb16_ohne boden_less30_onv"
 AS
select a.geom, a.id, a.name, b.*
from hot_fl.mtb16 a
left join hot_fl."10_pt_fl_count_ohne boden_less30_onv" b on (a.name||a.id) = b.mtb_id
;

create table hot_fl."11_tab_ptfl_count_mtb16_ohne boden_less30_onv"
as
select *
from hot_fl."11_ptfl_count_mtb16_ohne boden_less30_onv";

ALTER TABLE hot_fl."11_tab_ptfl_count_mtb16_ohne boden_less30_onv"
    ALTER COLUMN geom TYPE geometry(MultiPolygon,25832) USING ST_Multi(ST_Force2d(geom
    ));
```
