# Supplementary material for: Identification of hotspots of crop wild relatives in Germany to promote their in situ conservation in a network of genetic reserves
Source: Bot Stud. 2025 Sep 2;66:27. doi: 10.1186/s40529-025-00473-z (PMC12405085; doi:10.1186/s40529-025-00473-z)

Supplementary information 5

This document includes supplementary information on the article

"Identification of hotspots of crop wild relatives in Germany to promote their *in situ* conservation in a network of genetic reserves"

Maria Bönisch[1], Vera Senße[2], Thomas Engst[2], Alica Sander[2], Diethart Matthies[3], Eckhard Jedicke[4], Nadine Bernhardt[1]

[1]Julius Kühn-Institut (JKI) - Federal Research Centre for Cultivated Plants, Institute for Resistance Research and Stress Tolerance, Quedlinburg, Germany

[2]Anhalt University of Applied Sciences, Department of Agriculture, Ecotrophology and Landscape Development, Bernburg, Germany

[3] Philipps-Universität Marburg, Plant Ecology and Geobotany, Marburg, Germany

[4] Hochschule Geisenheim University, Department of Landscape Planning & Nature Conservation, Geisenheim, Germany

Corresponding authors: Maria Bönisch (maria.boenisch@julius-kuehn.de), Nadine Bernhardt (nadine.bernhardt@julius-kuehn.de)

This document visualises the location of the 27 GR candidates. The abbreviation, the name of the respective grid cell and the map resolution are given for each map.

Source of basemap: Map data © OpenStreetMap contributors. Data available under the Open Database License (ODbL). See https://www.openstreetmap.org/copyright, accessed on 2025-08-03

BA - Bamberg Süd34554 (1:72194)

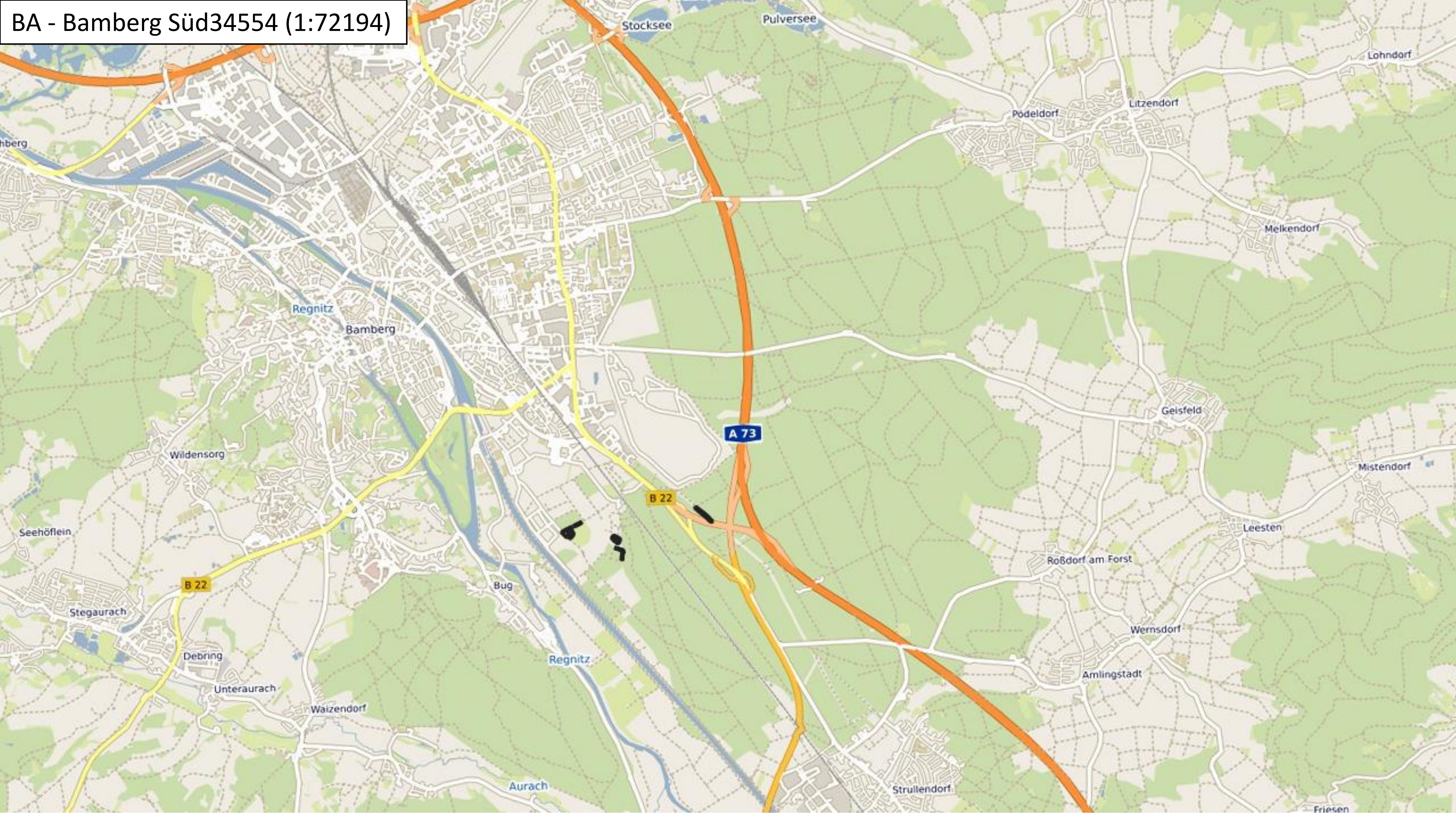

BR - Bayrischzell47339 (1:52337)

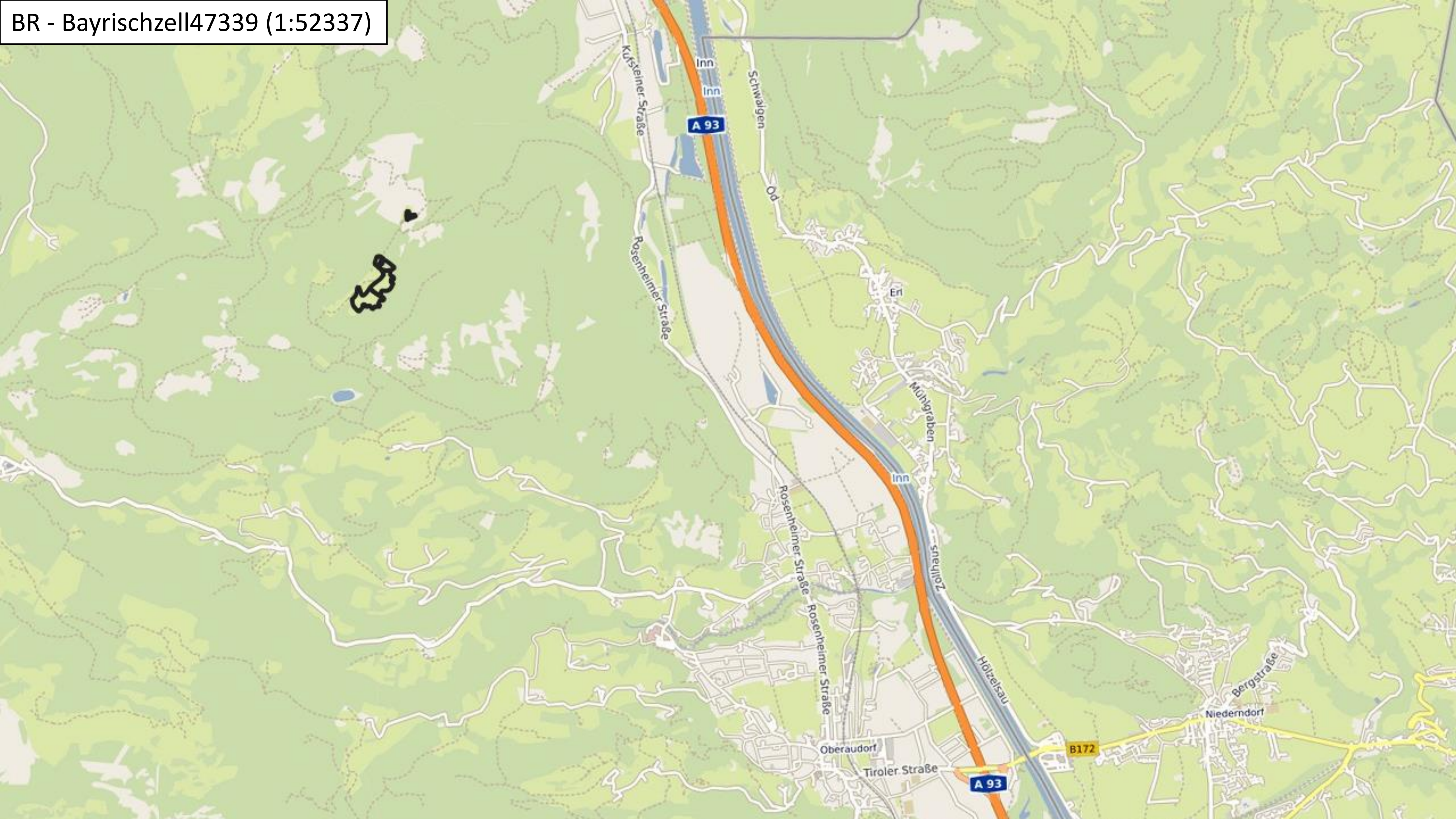

BS - Bischofsheim an der Rhön30685 (1:55954)

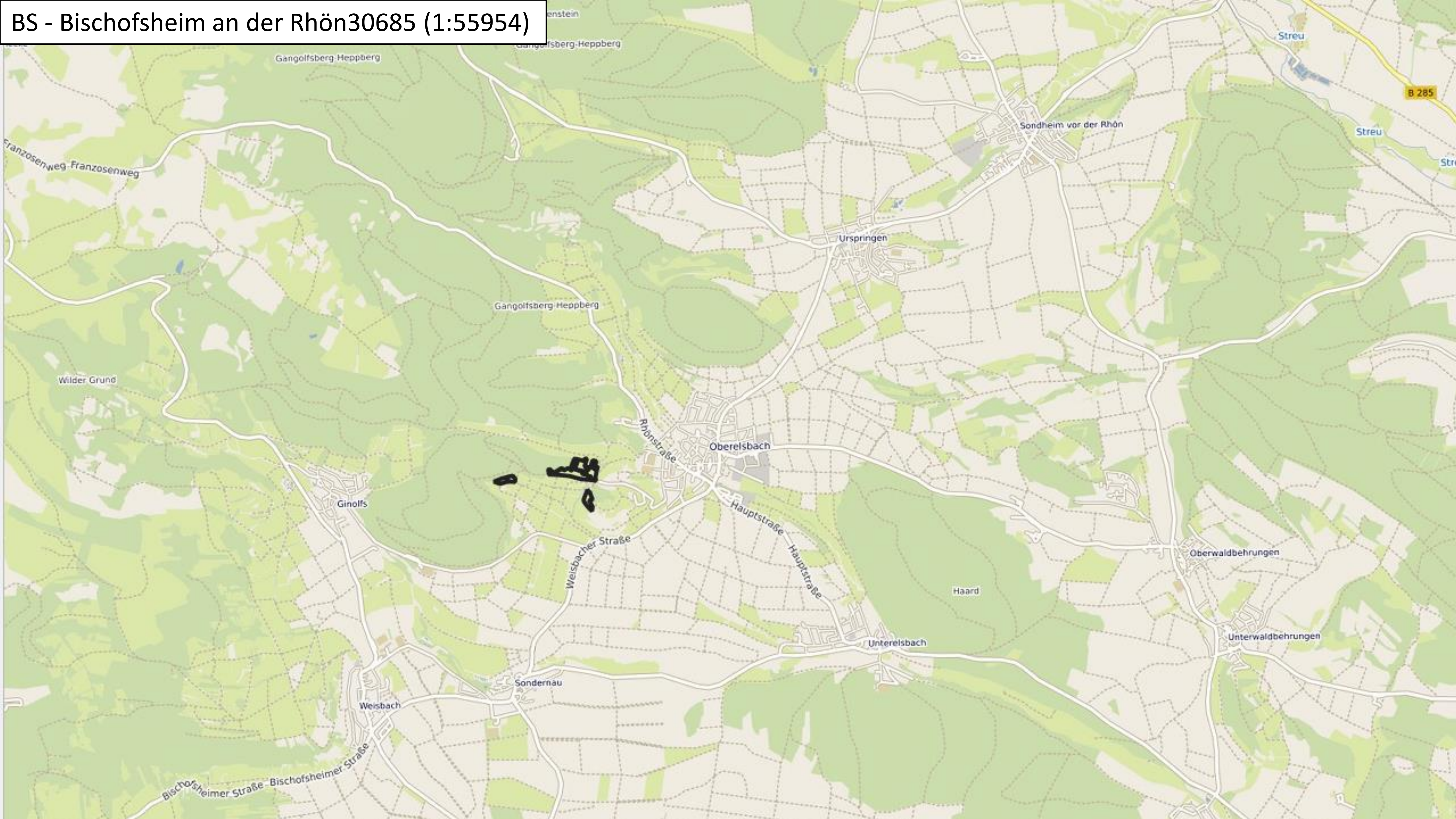

BT - Bitterfeld (West)20860 (1:49483)

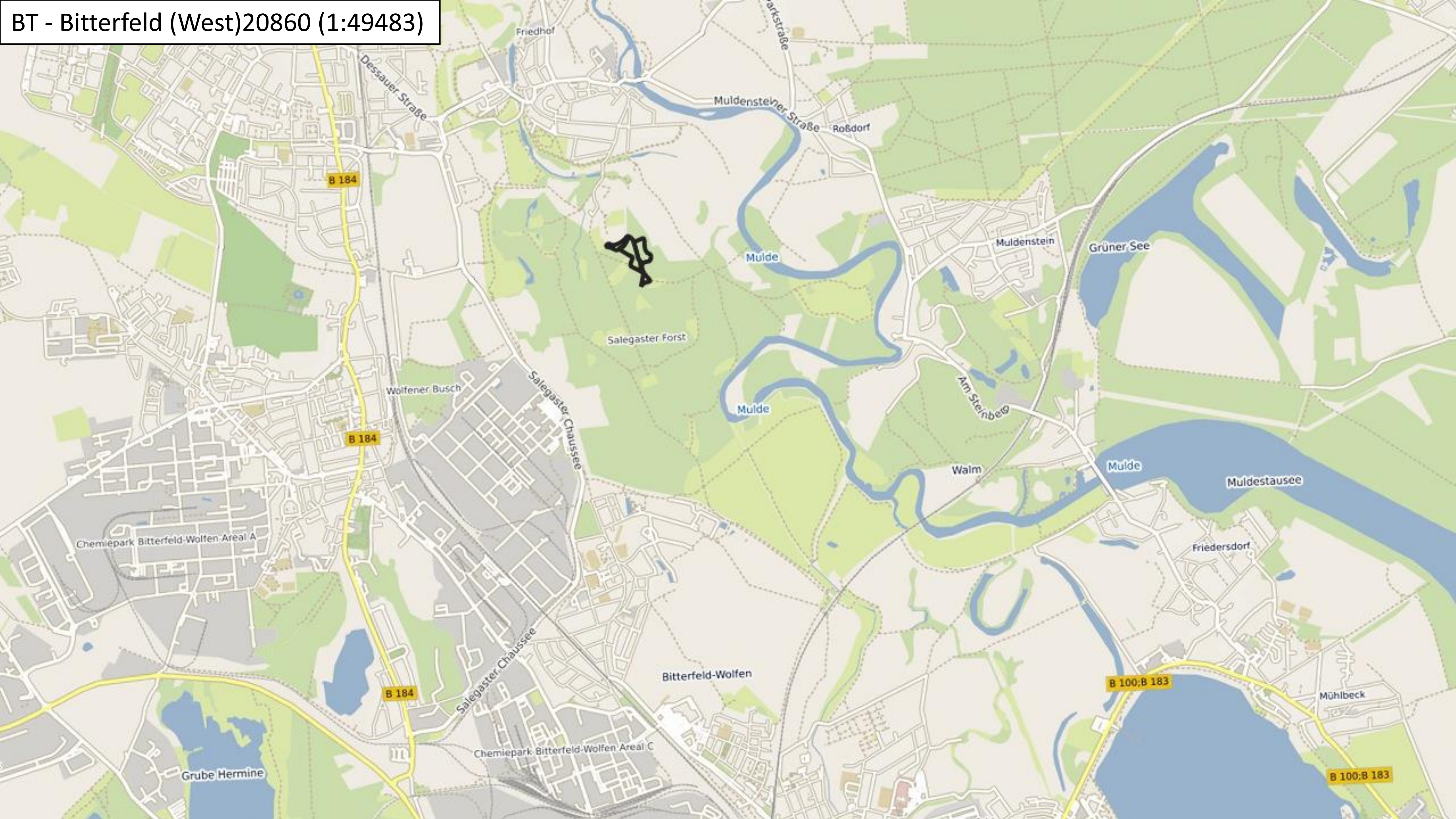

BL - Blankenburg19885 (1:70849)

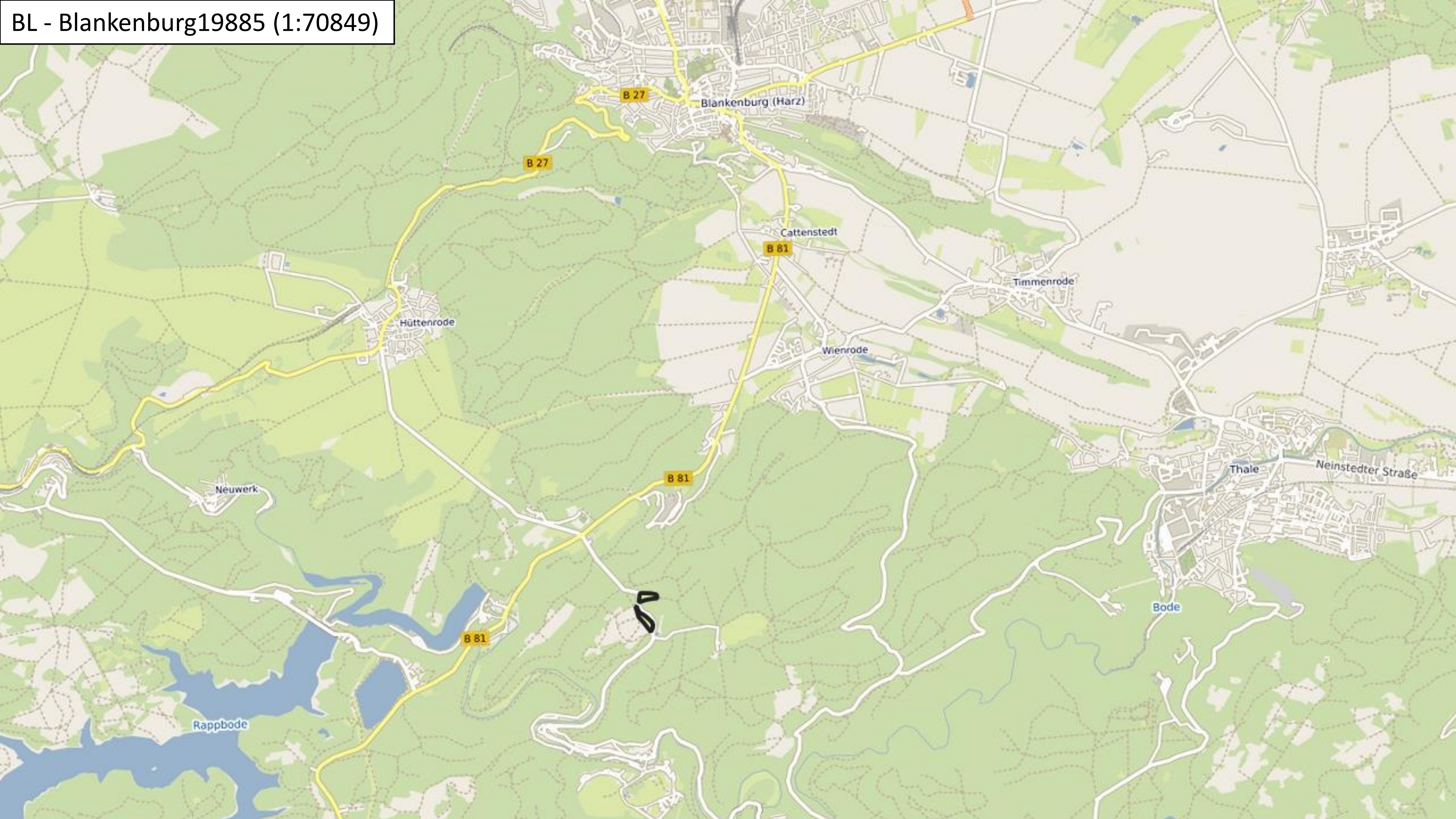

FN - Fünfeichen16896 (1:45631)

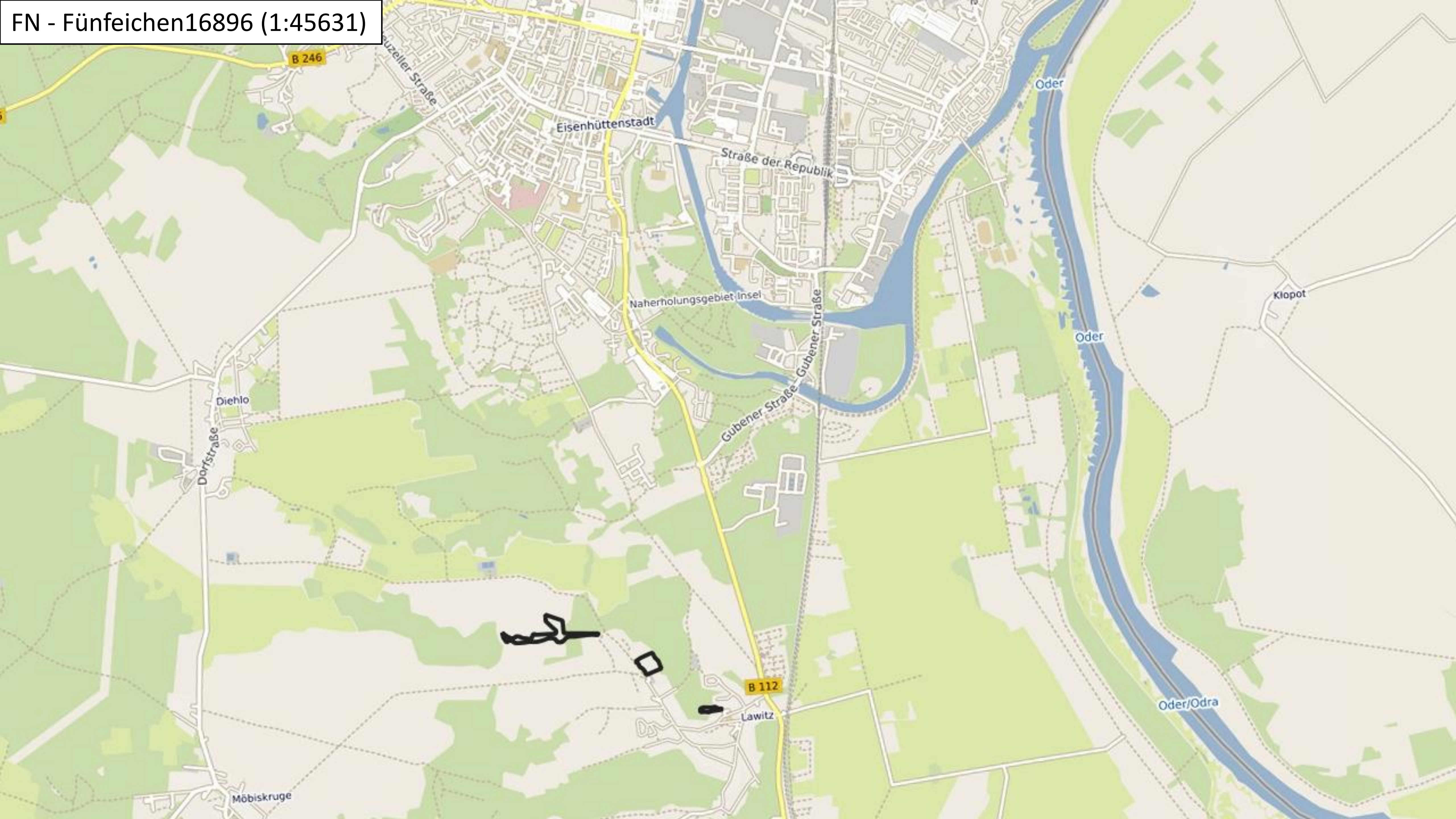

FR - Fürstenberg i. Mecklenburg9352 (1:37270)

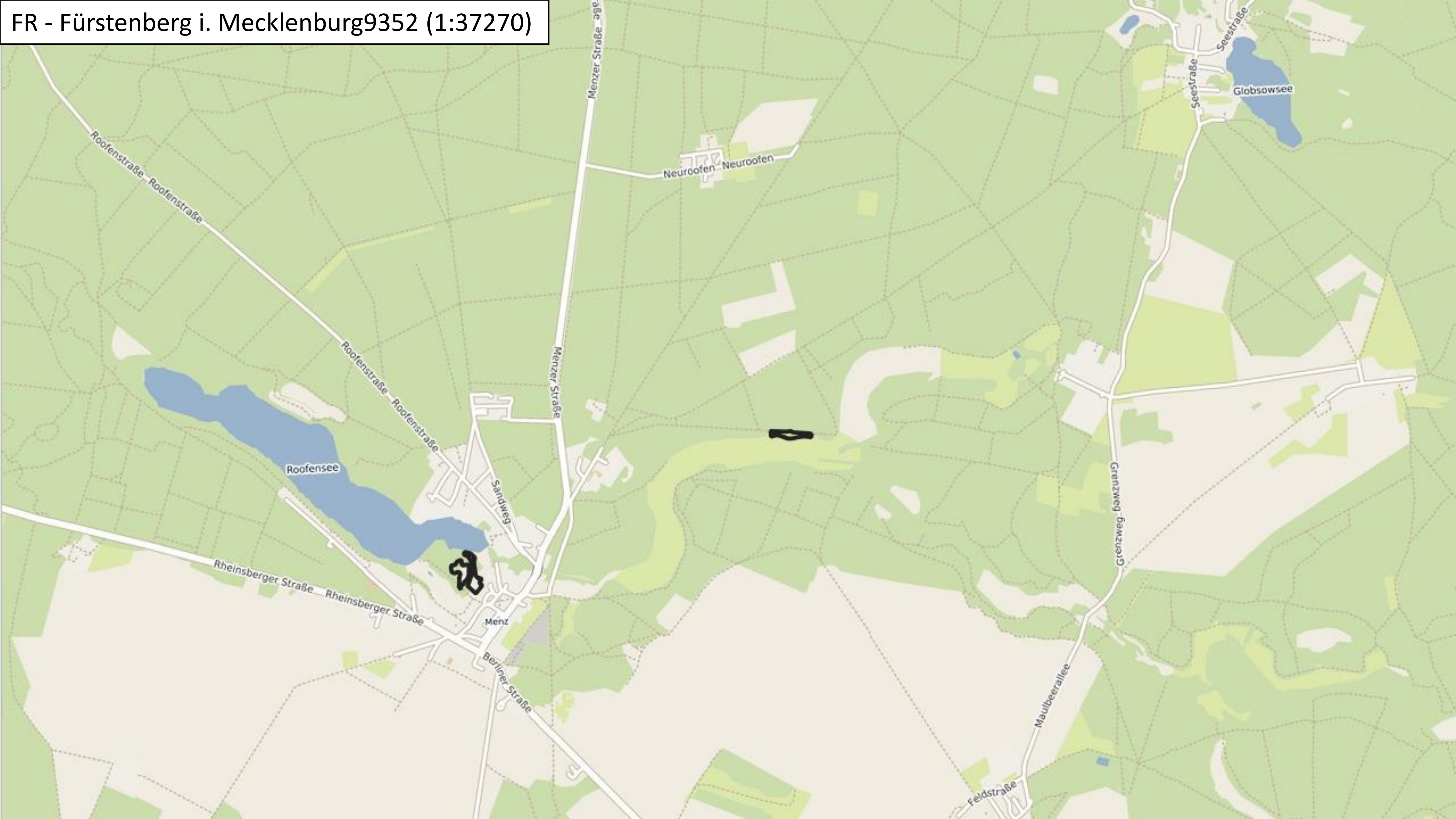

G - Güsten19942 (1:71144)

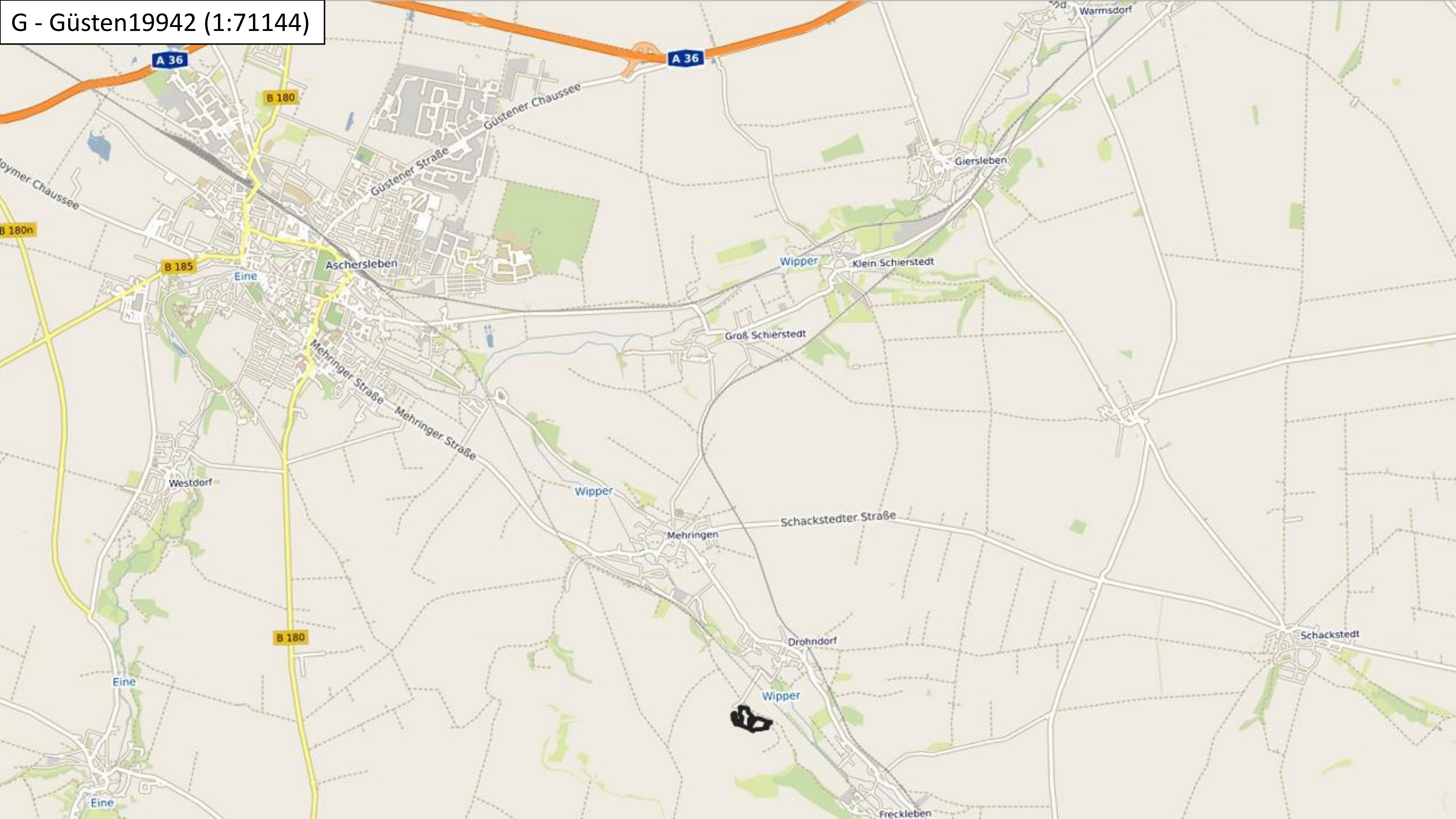

Hb - Halberstadt19038 (1:40595)

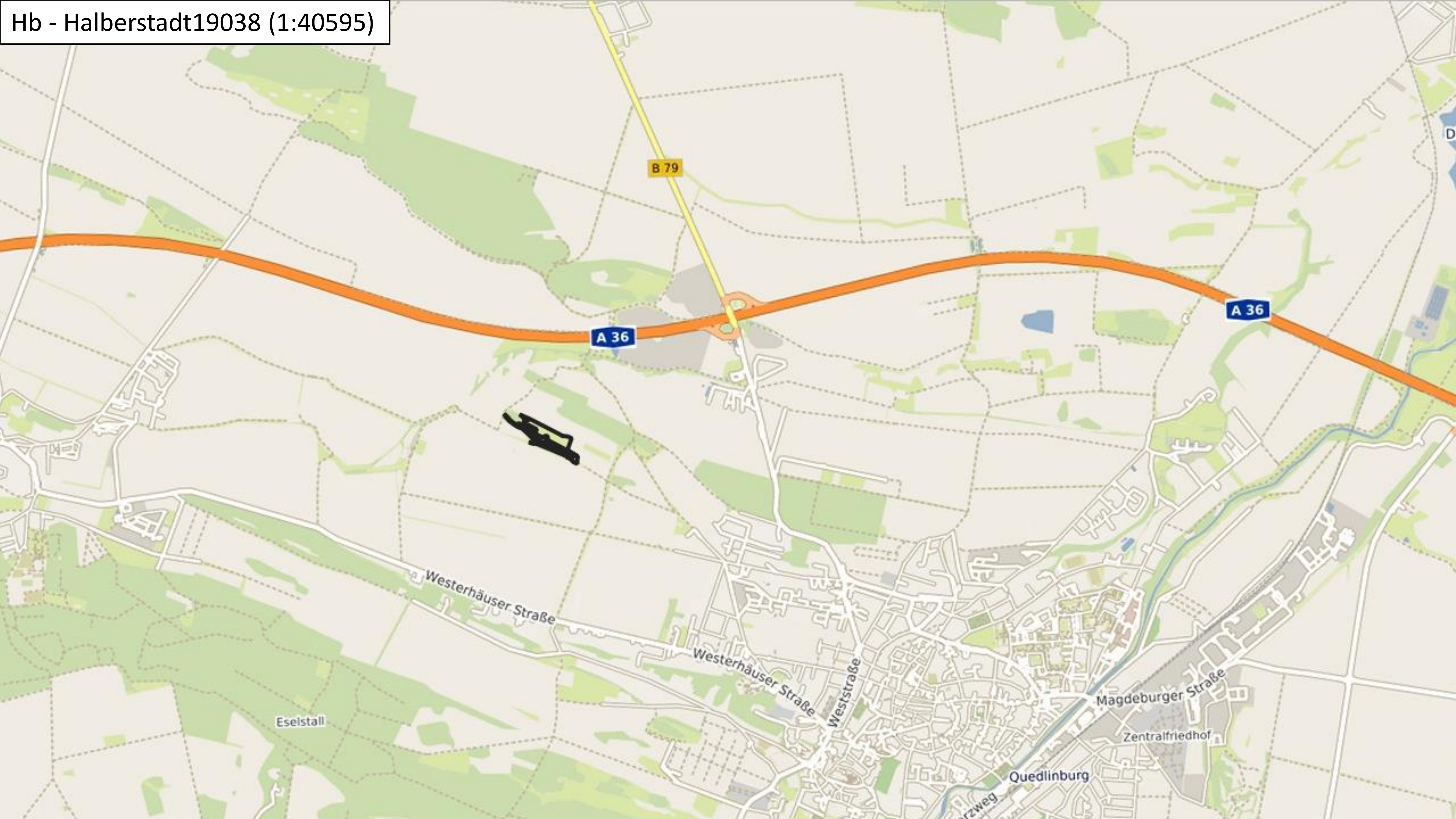

HL - Halle (Westfalen)17079 (1:39483)

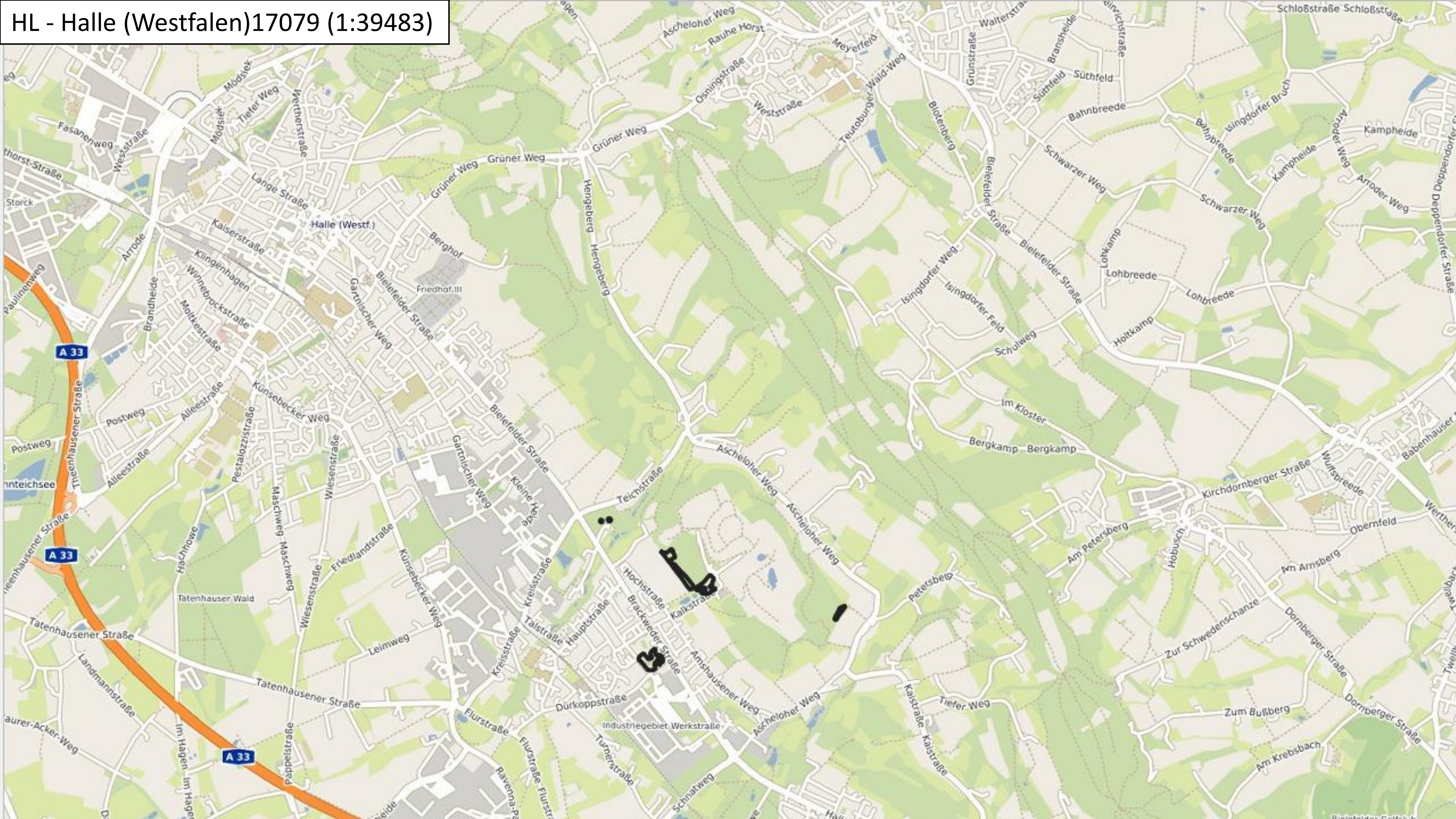

HI - Hildburghausen30748 (1:31994)

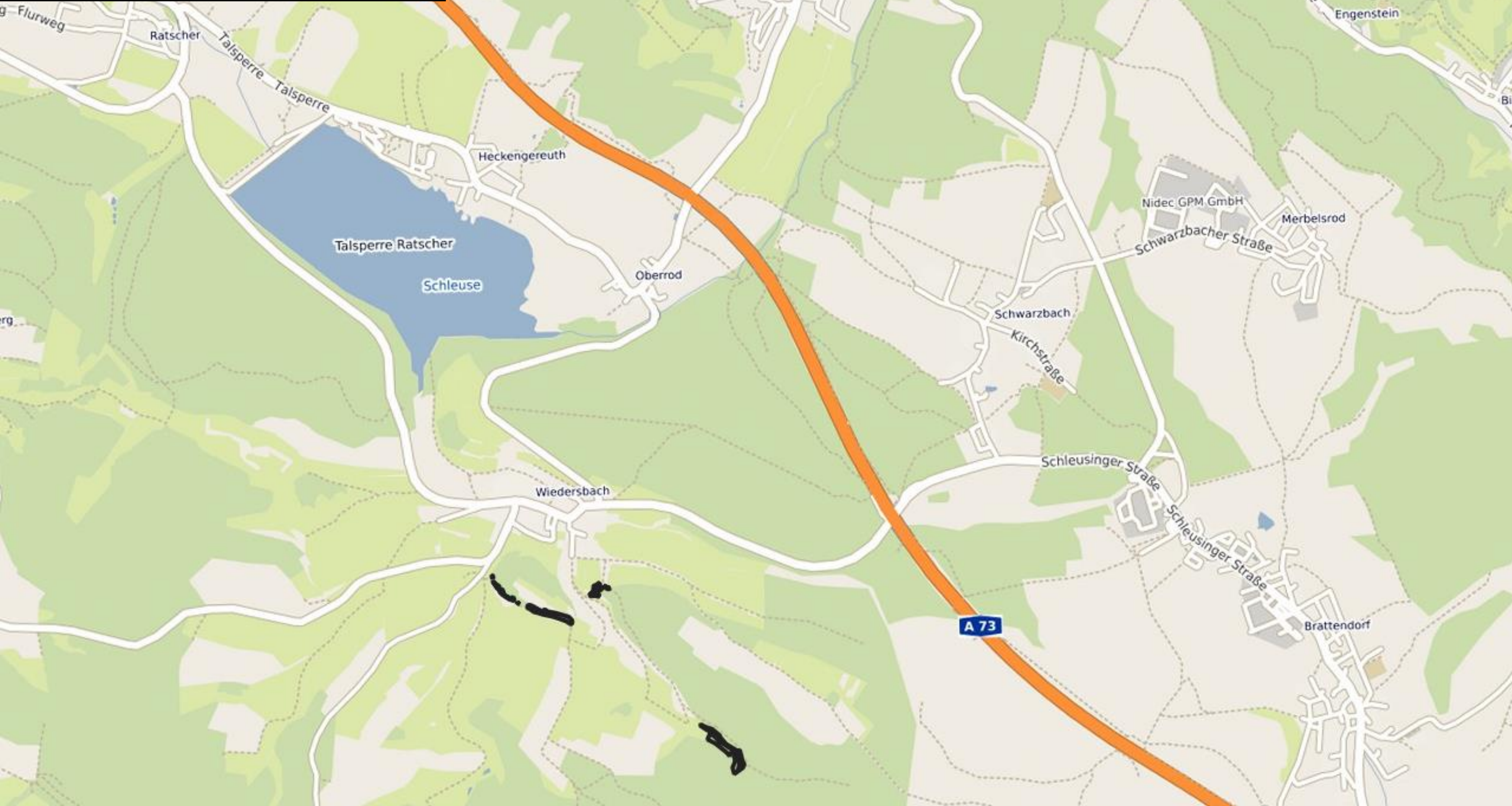

K - Kelbra22443 (1:56534)

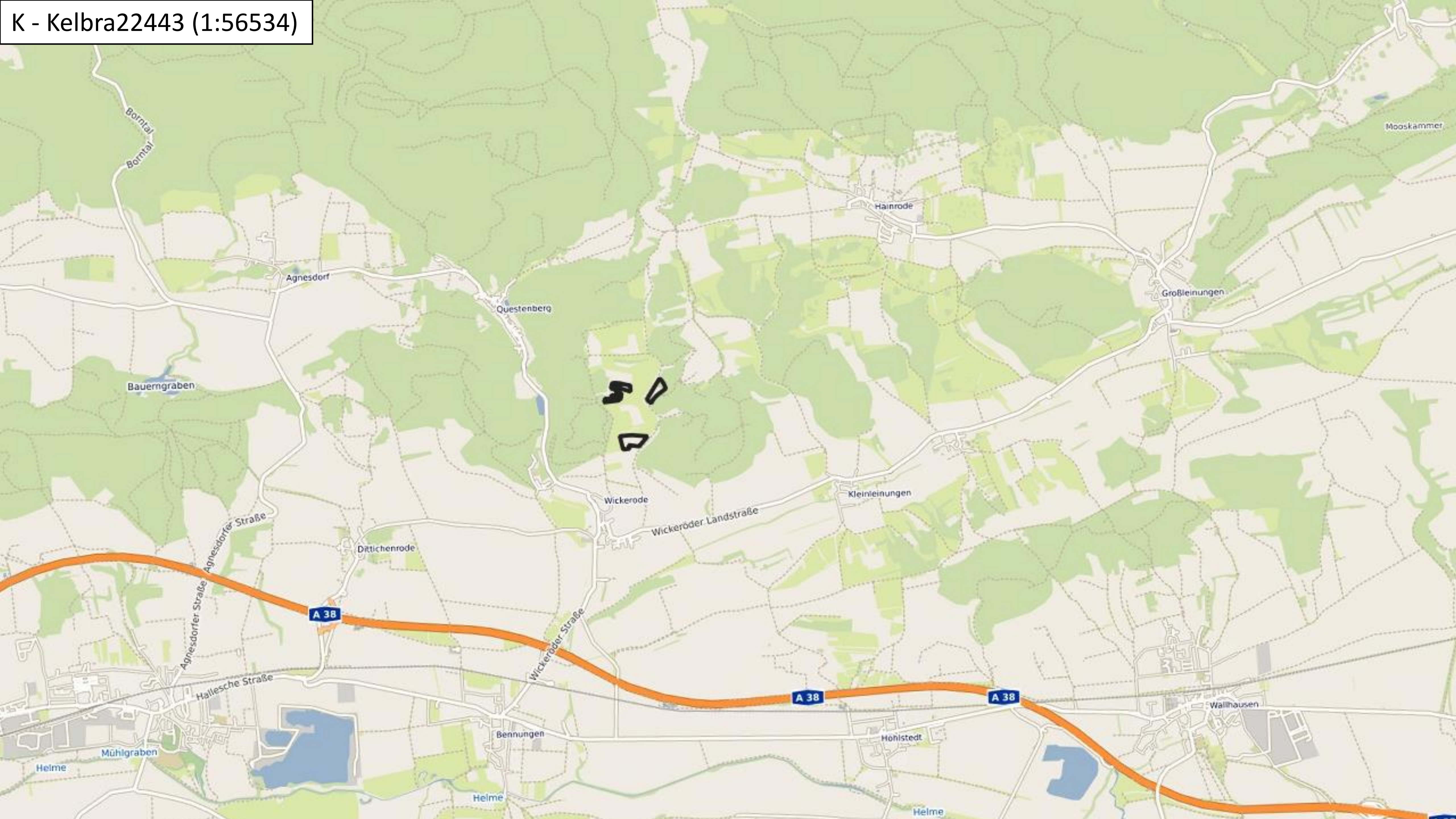

L - Libbenichen14604 (1:51179)

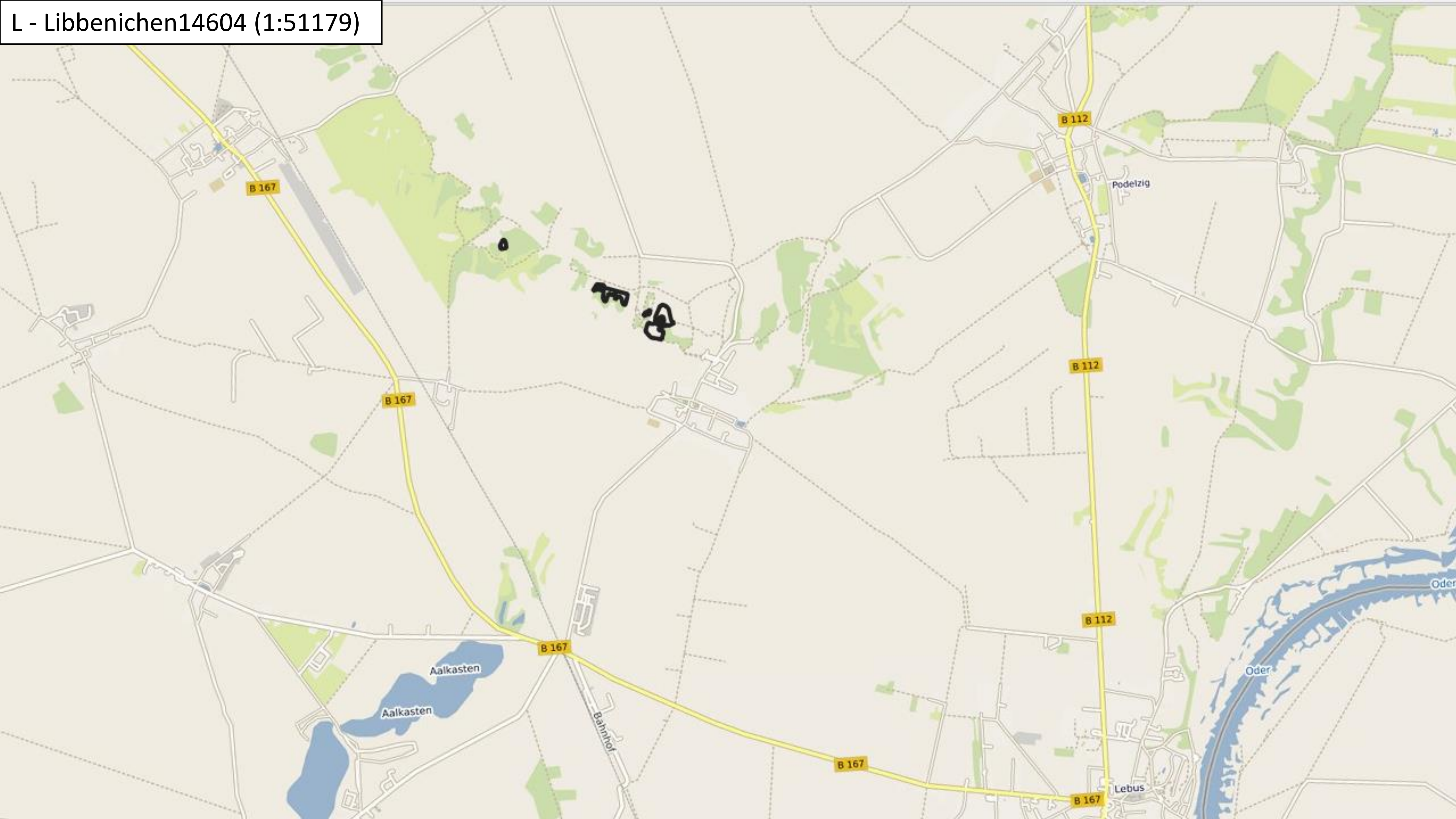

MN - Mansfeld21621 (1:38145)

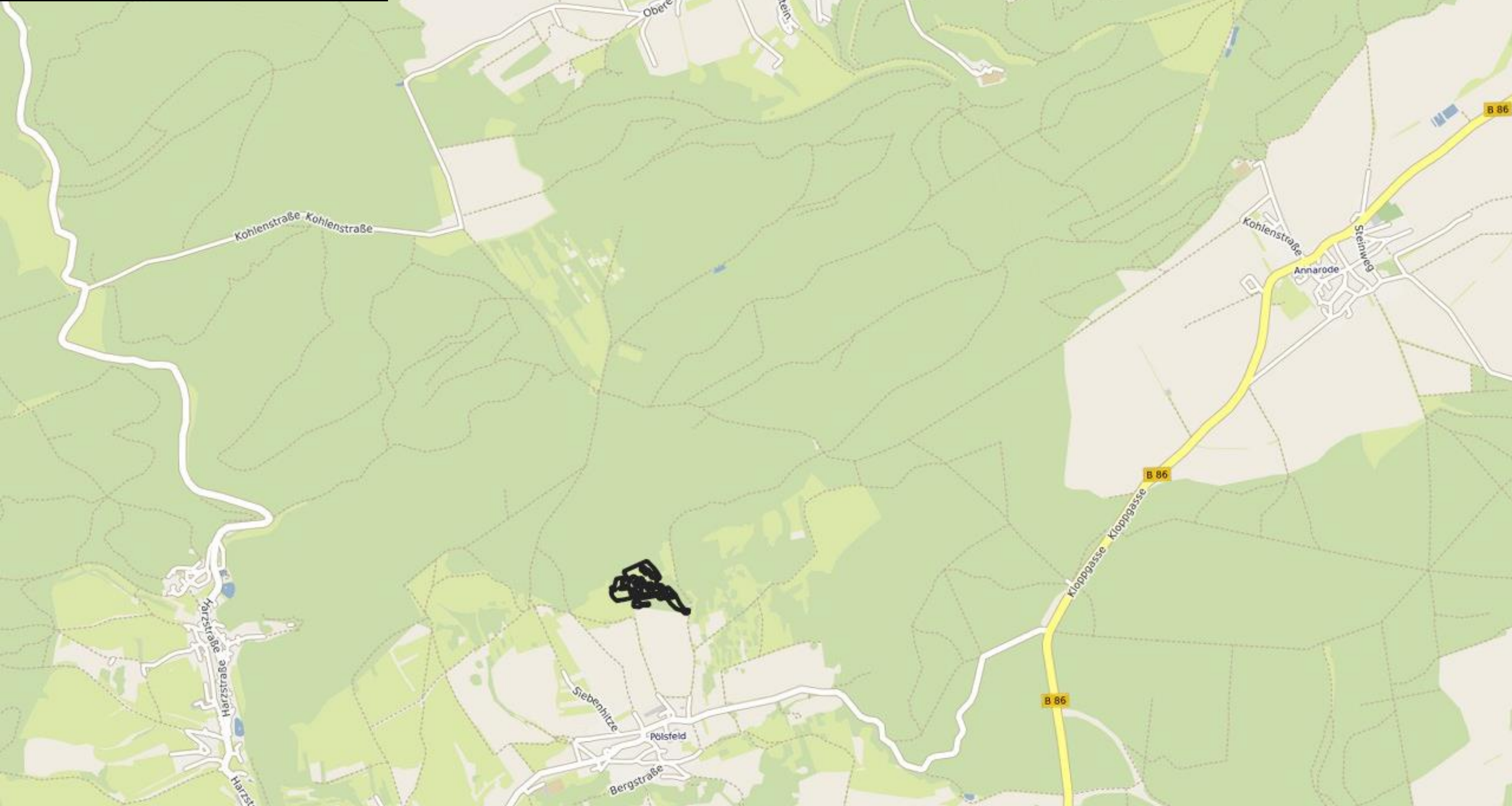

MR - Marsberg22229 (1:45689)

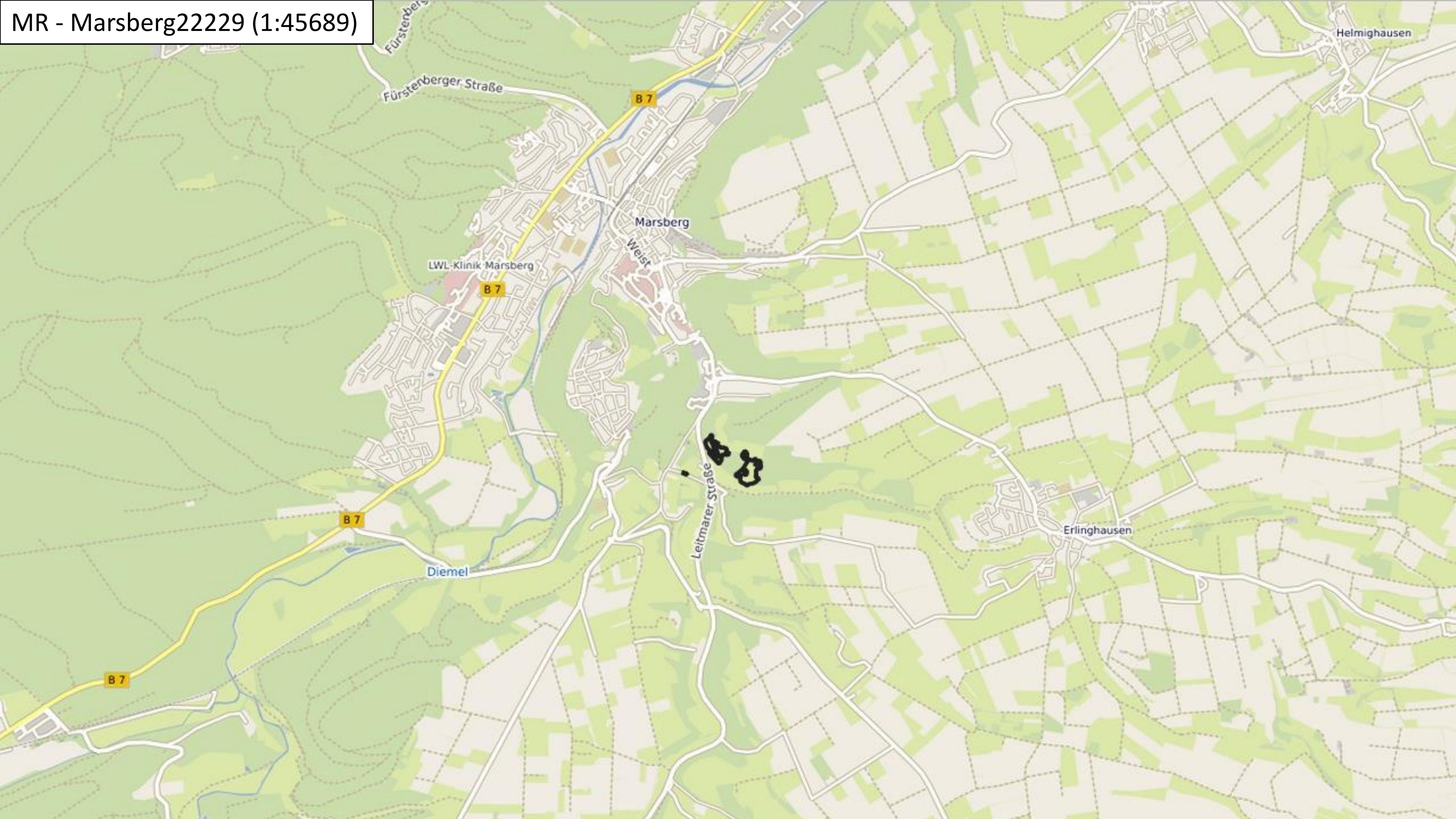

NB - Neubrandenburg6467 (1:56827)

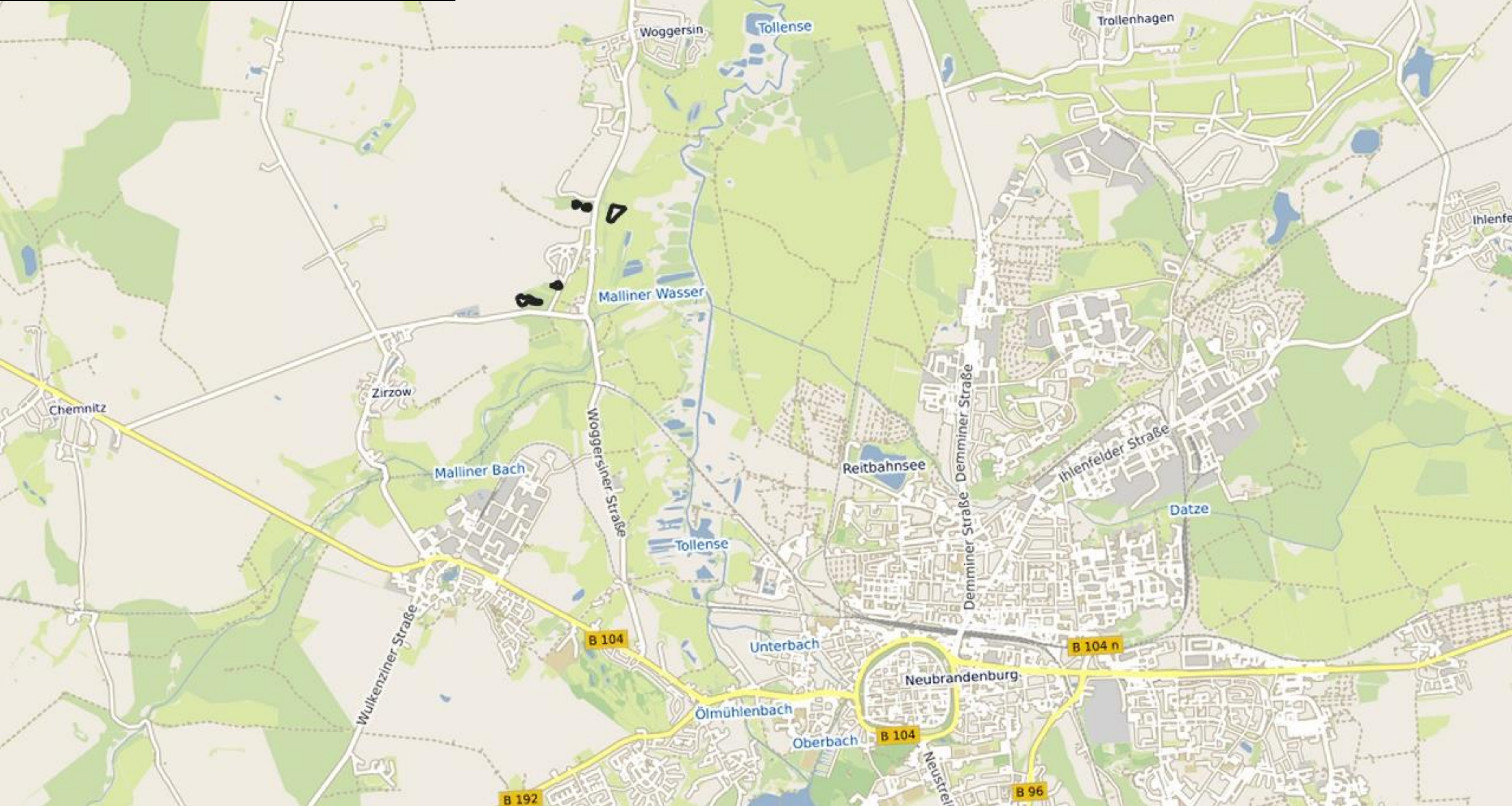

O - Osterwieck18131 (1:40595)

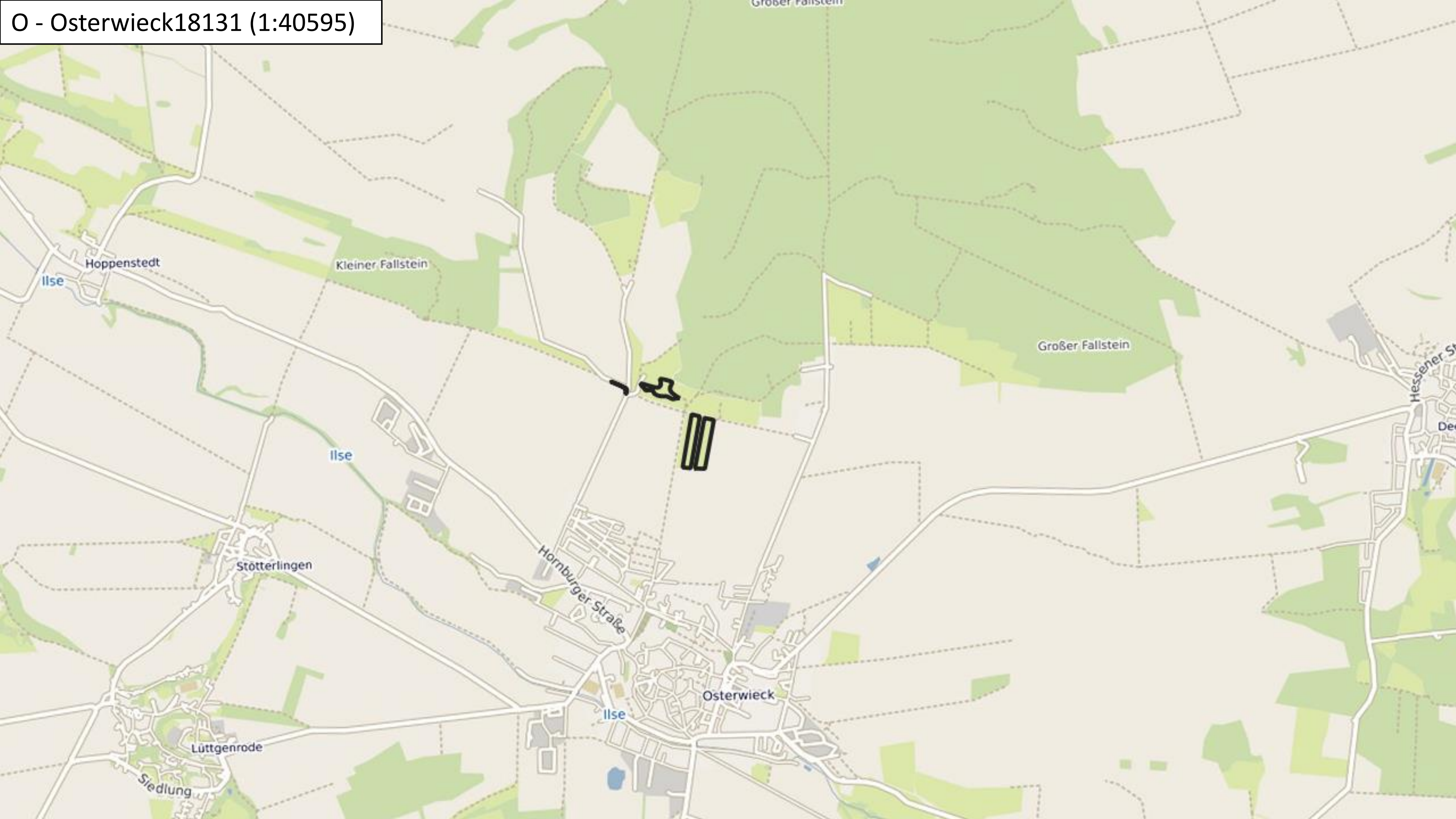

RA - Rätzlingen14285 (1:81548)

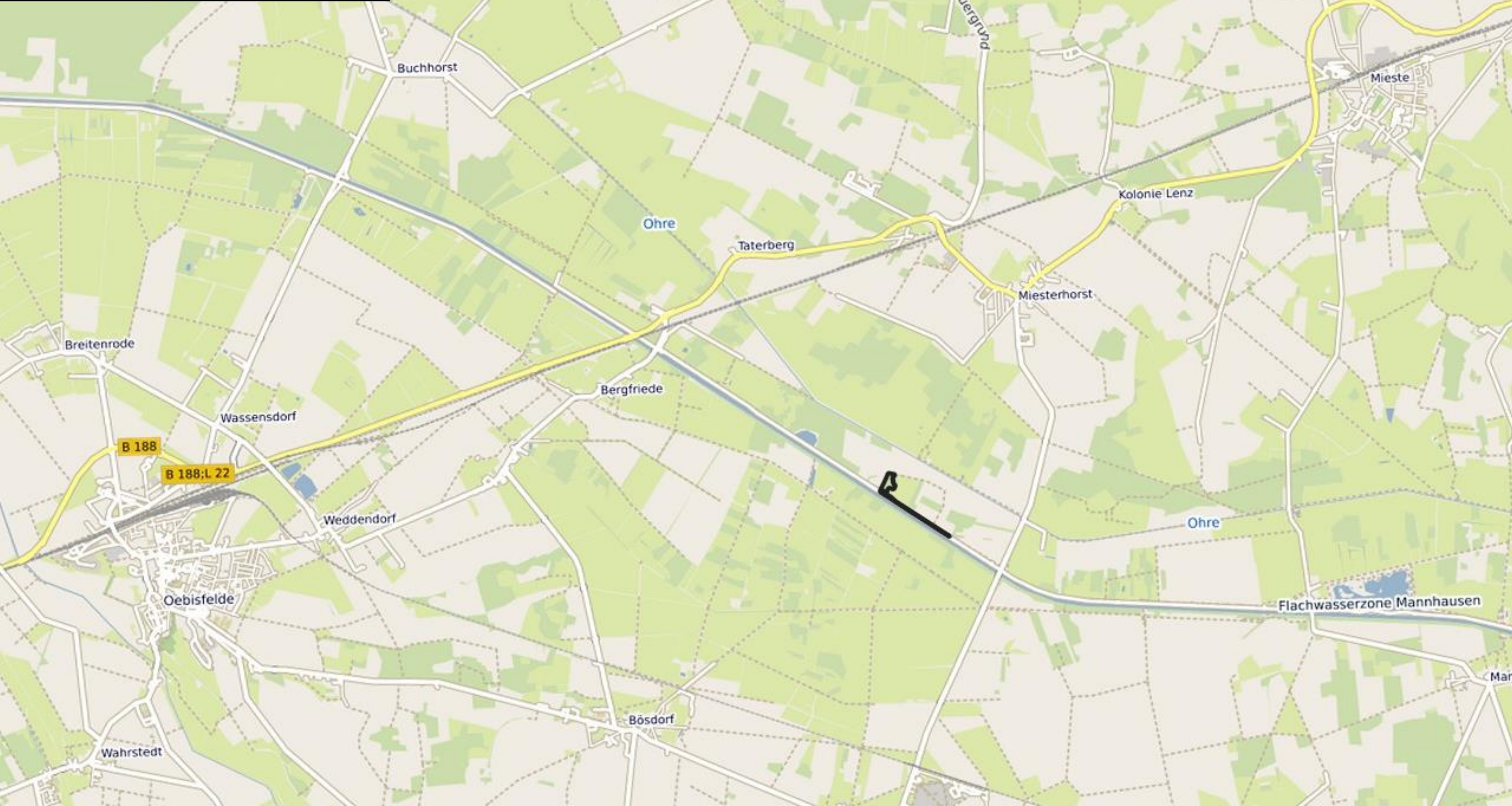

RO - Rogätz15086 (1:57163)

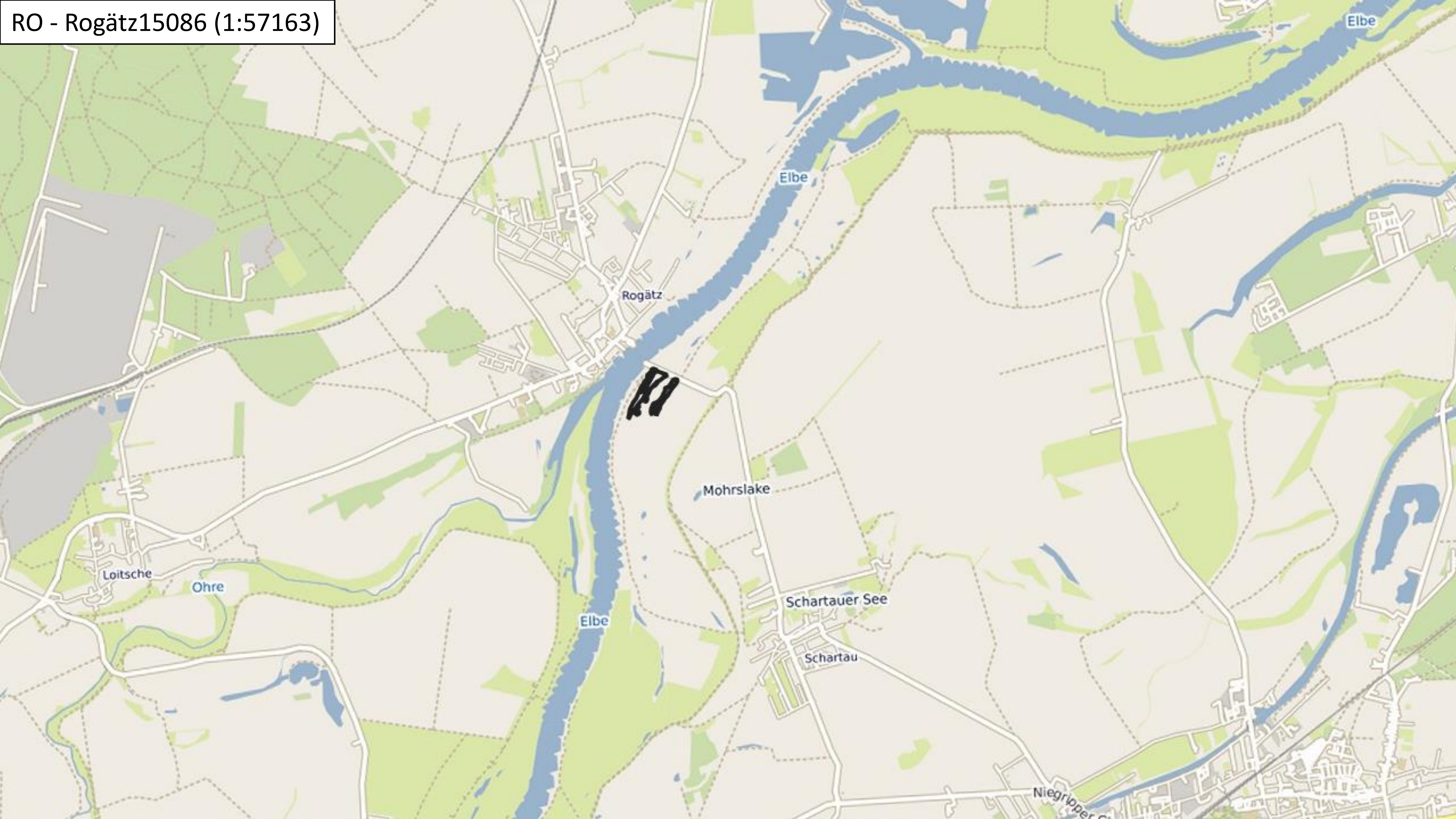

SC - Schnackenburg9908 (1:46578)

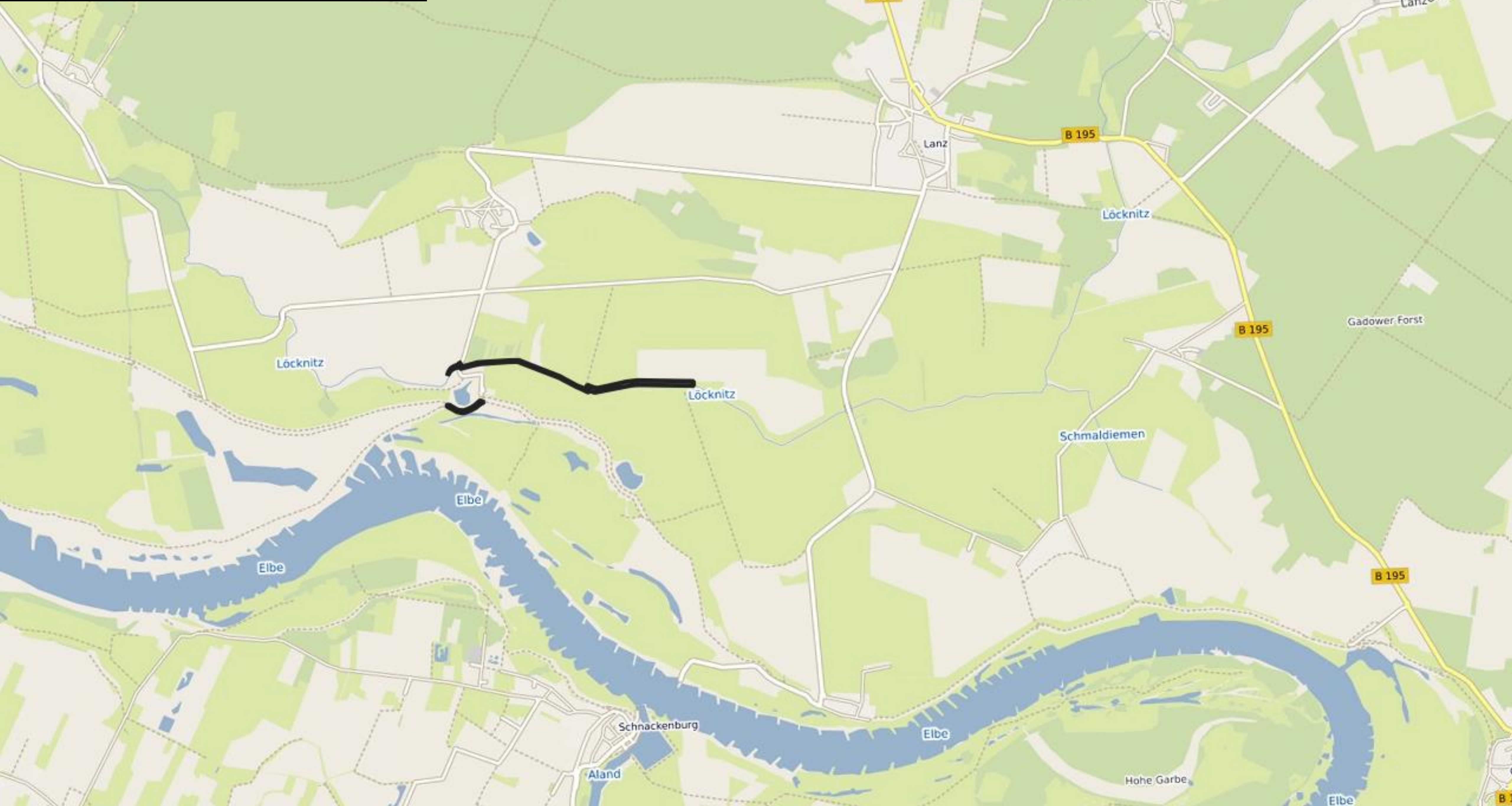

SE - Seyda19206 (1:35517)

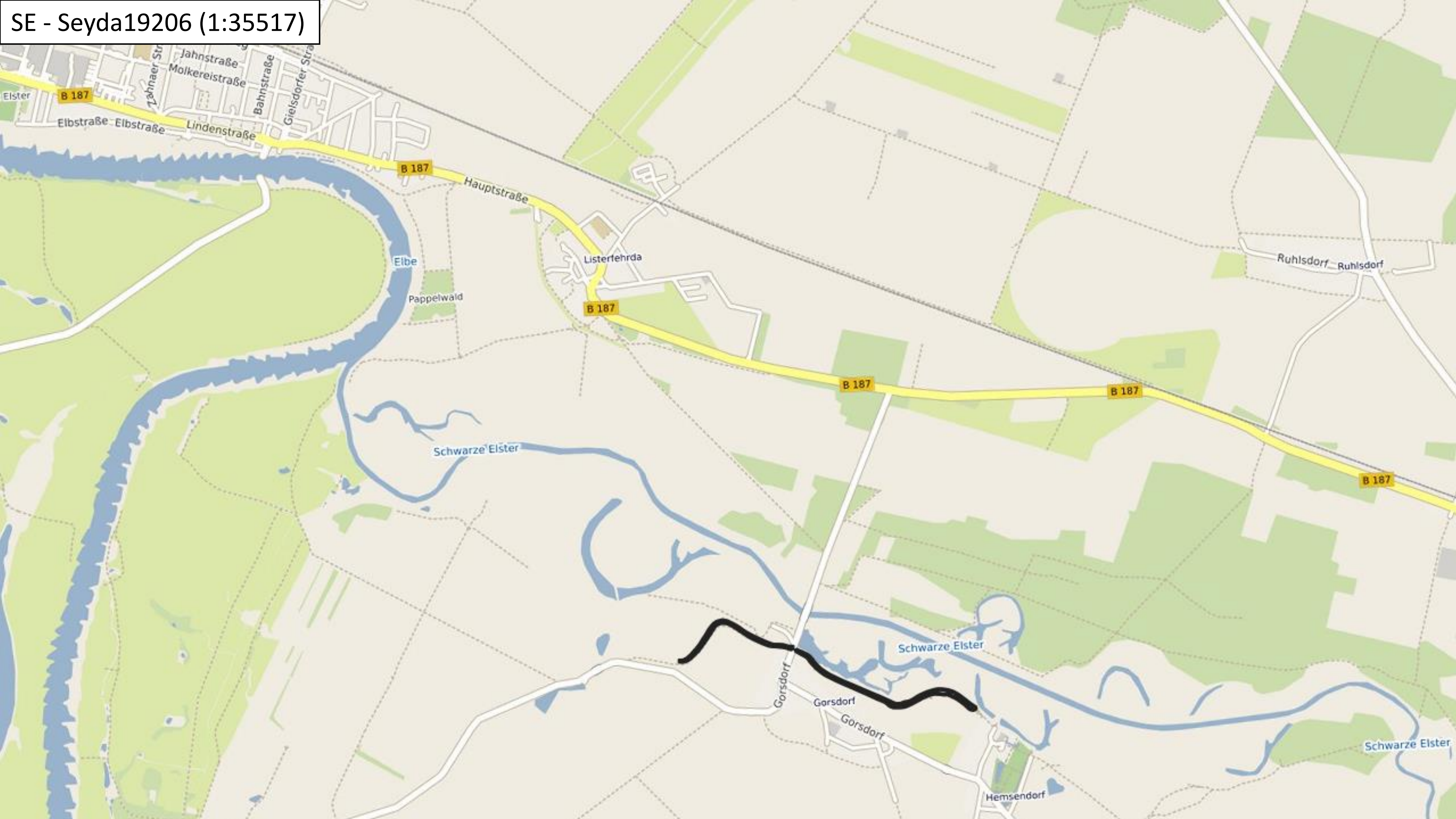

ST - Stolpe i.d. Mark10873 (1:52507)

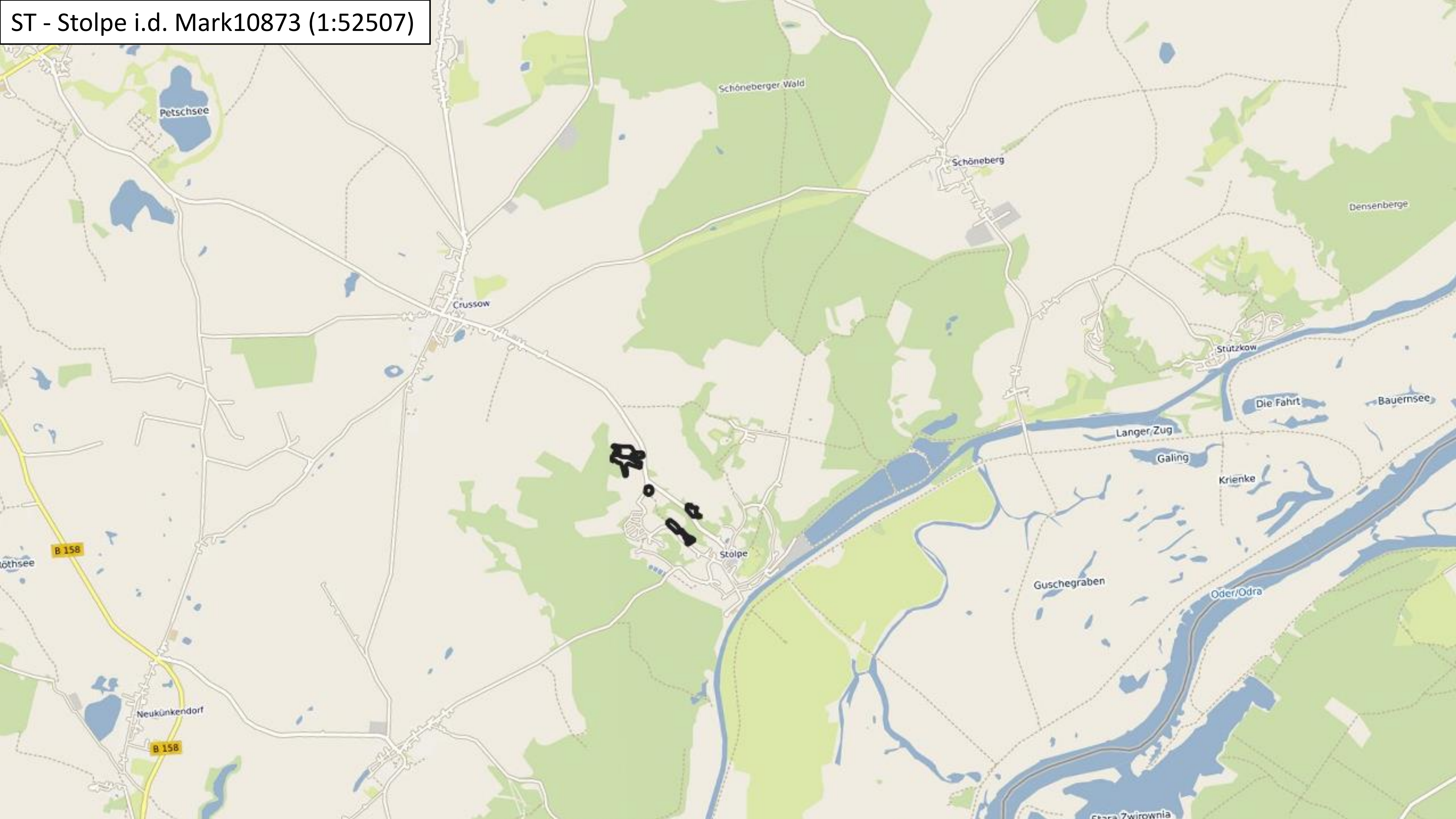

WA - Wallmow8048 (1:56372)

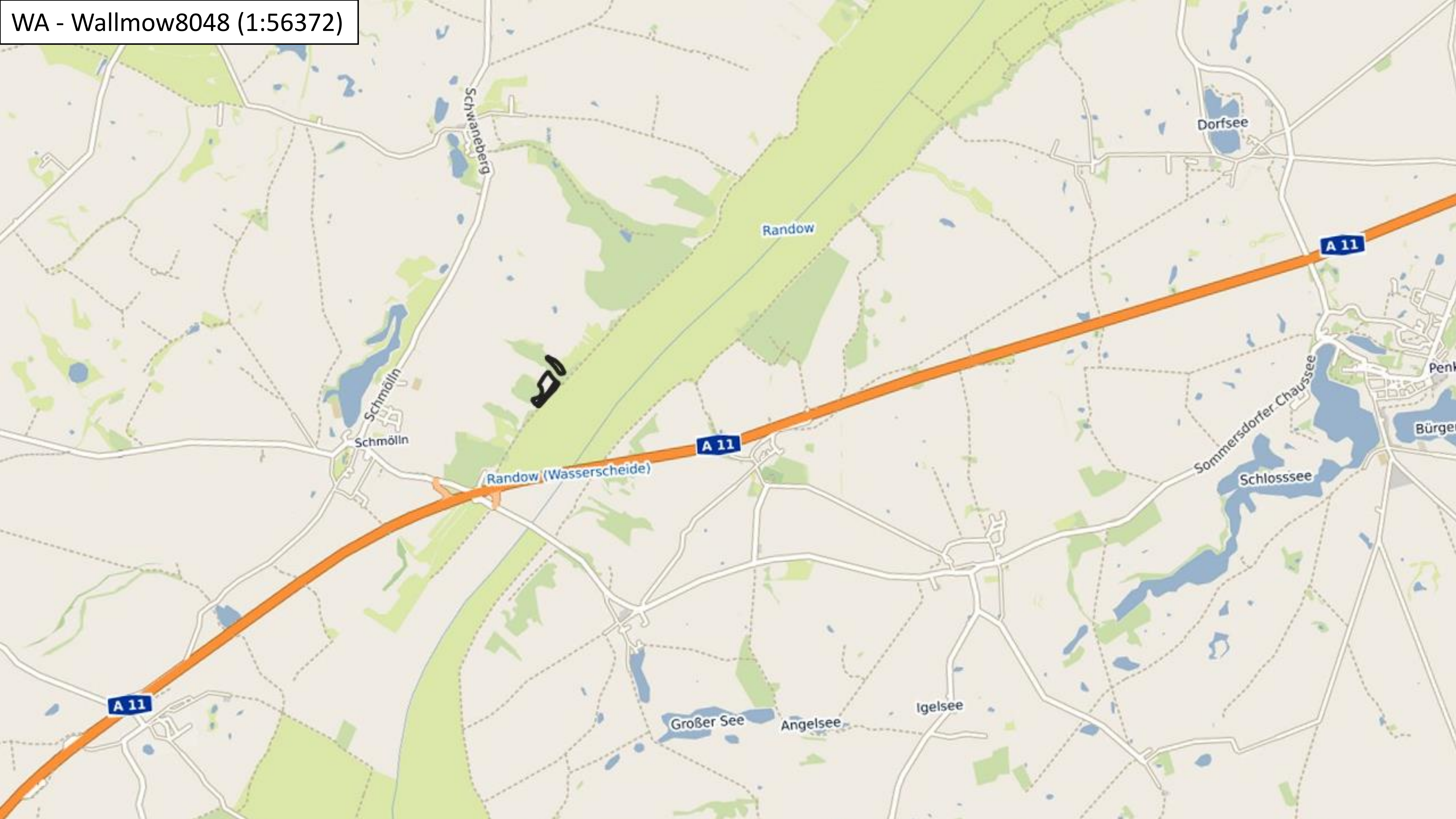

WI - Weißewarte14363 (1:49525)

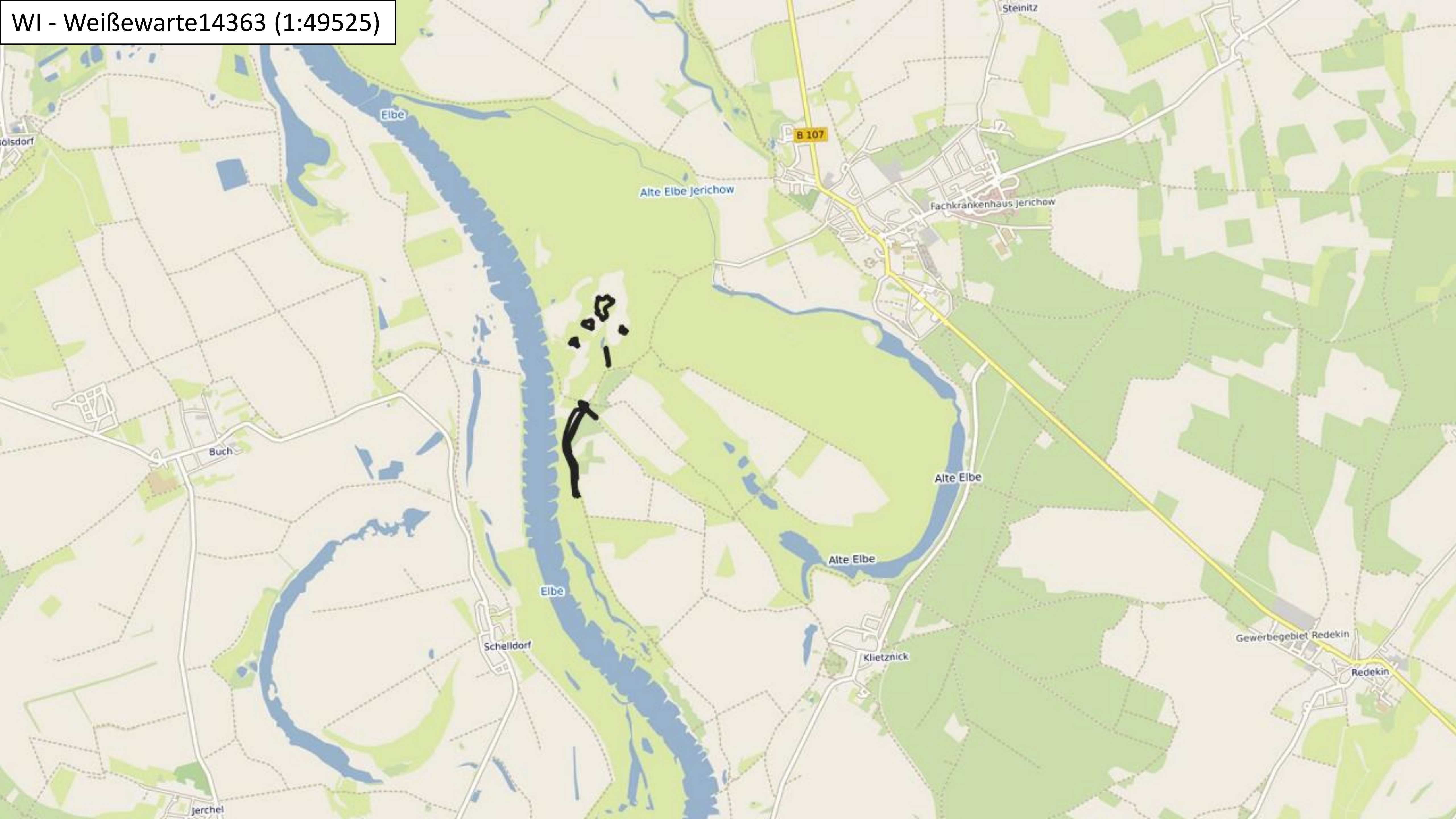

WR - Werben -Elbe11371 (1:33077)

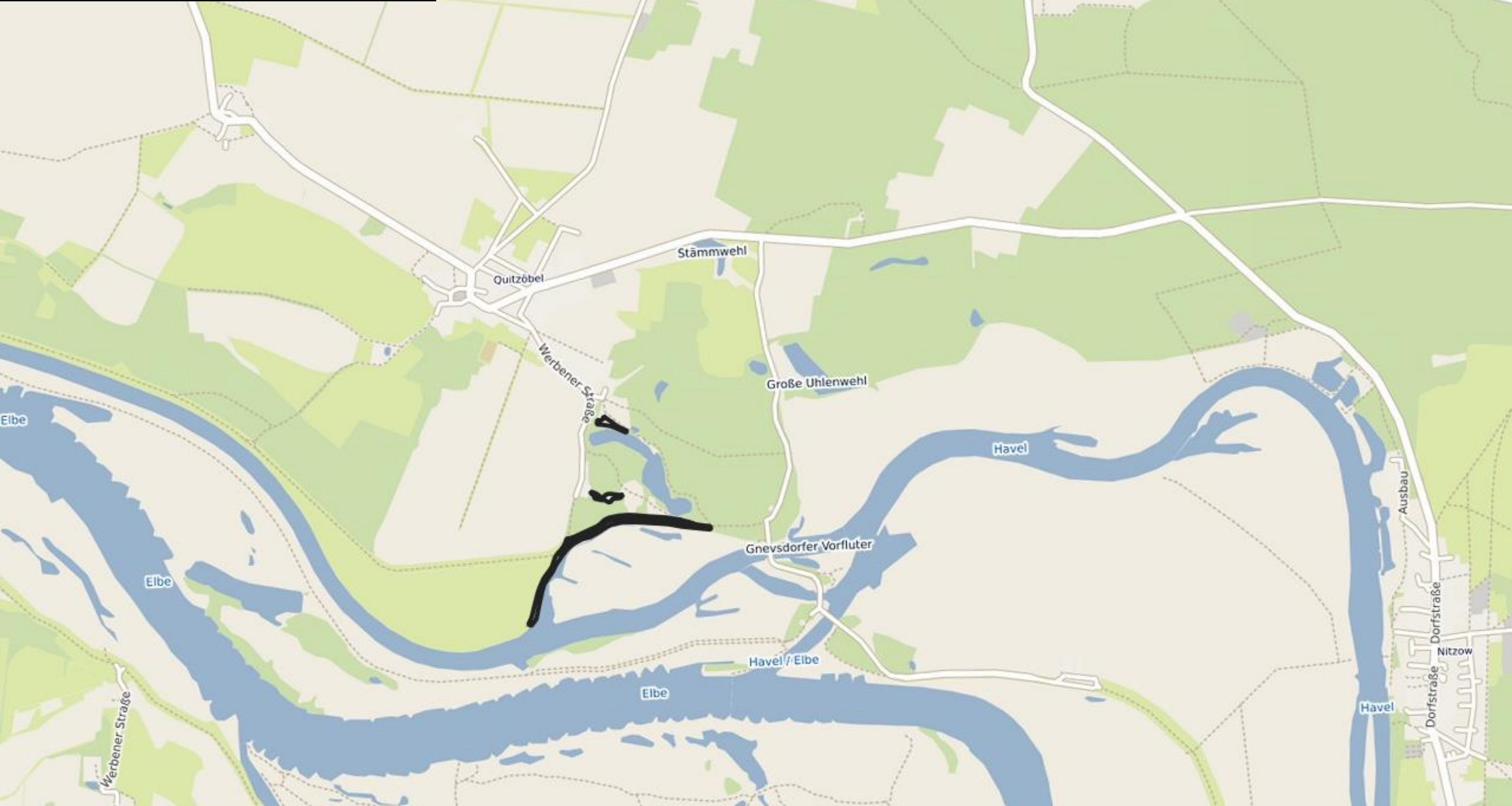

WL - Wildenbruch i.d. Mark15956 (1:51311)

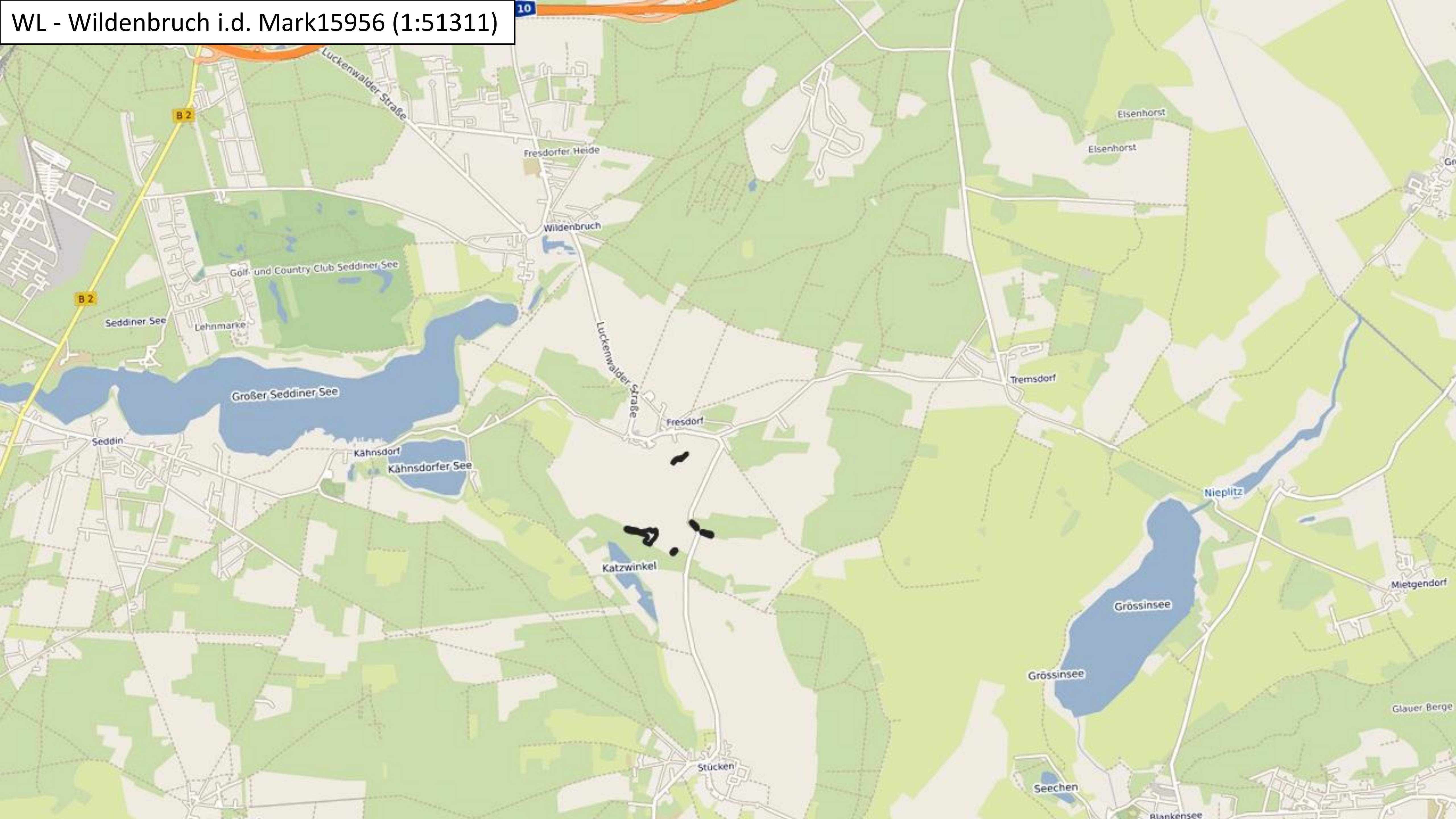

Z - Zittau (Nord)27192 (1:21556)

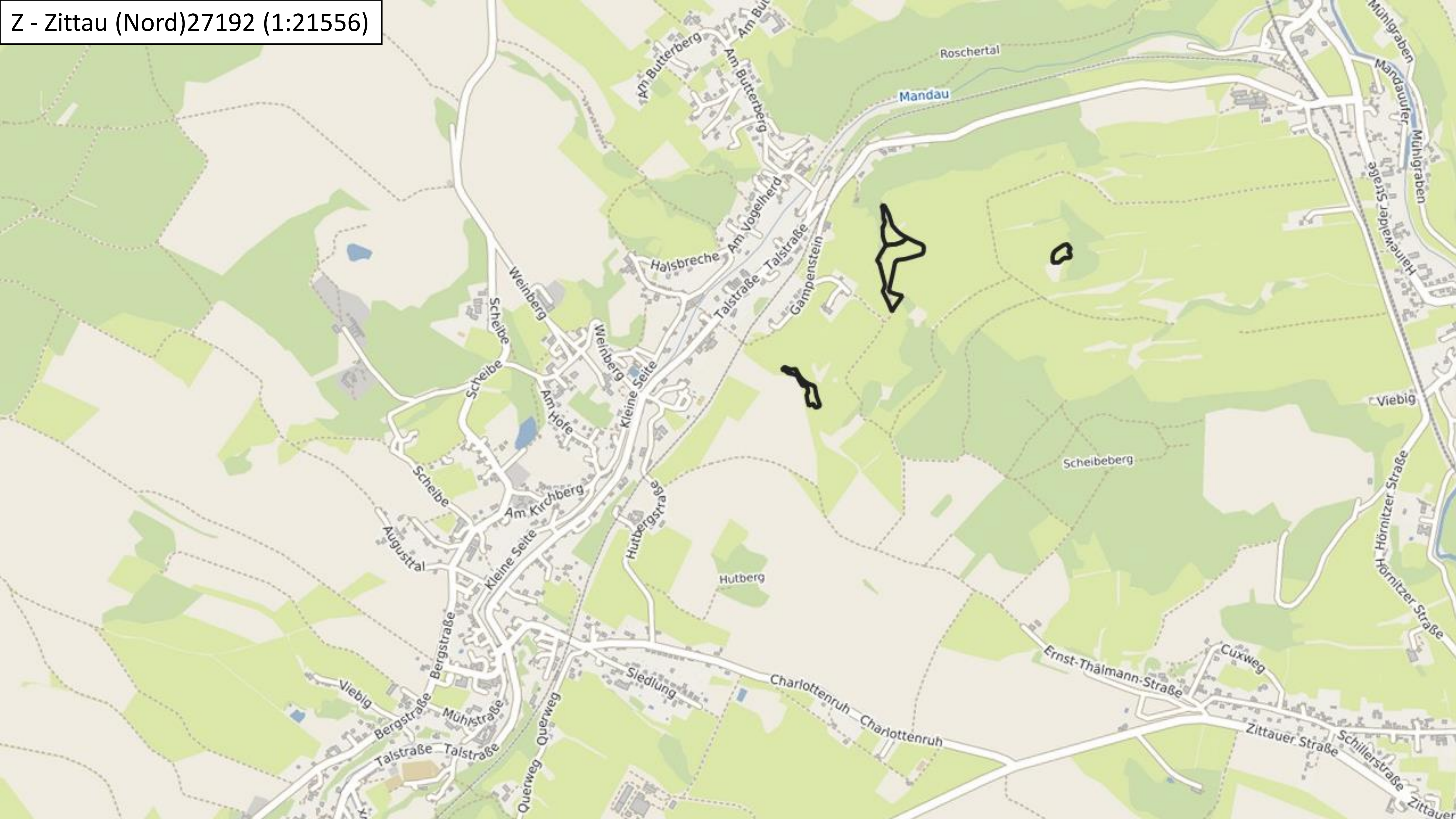

Supplement: Supplementary file 5 — Supplementary Material 5 [file 40529_2025_473_MOESM5_ESM.pdf]
